# Computational validation of clonal and subclonal copy number alterations from bulk tumor sequencing using CNAqc

**DOI:** 10.1186/s13059-024-03170-5

**Published:** 2024-01-31

**Authors:** Alice Antonello, Riccardo Bergamin, Nicola Calonaci, Jacob Househam, Salvatore Milite, Marc J. Williams, Fabio Anselmi, Alberto d’Onofrio, Vasavi Sundaram, Alona Sosinsky, William C. H. Cross, Giulio Caravagna

**Affiliations:** 1 Evolution and Cancer Lab, Centre for Genomics and Computational Biology, Barts Cancer Institute, Barts and the London School of Medicine and Dentistry, Queen Mary University of London, London, UK; 2 Department of Mathematics, Informatics and Geosciences (MIGe), University of Trieste, Trieste, Italy; 3 Centre for Computational Biology, Human Technopole, Milan, Italy; 4 Department of Computational Oncology, Memorial Sloan Kettering, New York, USA; 5 Department of Research Pathology, UCL Cancer Institute, University College London, London, UK; 6 Genomics England, London, UK; 7 Evolutionary Genomics and Modelling Team, Centre for Evolution and Cancer, Institute of Cancer Research, London, UK

## Abstract

Copy number alterations (CNAs) are among the most important genetic events in cancer, but their detection from sequencing data is challenging because of unknown sample purity, tumor ploidy, and general intra-tumor heterogeneity. Here, we present CNAqc, an evolution-inspired method to perform the computational validation of clonal and subclonal CNAs detected from bulk DNA sequencing. CNAqc is validated using single-cell data and simulations, is applied to over 4000 TCGA and PCAWG samples, and is incorporated into the validation process for the clinically accredited bioinformatics pipeline at Genomics England. CNAqc is designed to support automated quality control procedures for tumor somatic data validation.

**Supplementary Information:**

The online version contains supplementary material available at 10.1186/s13059-024-03170-5.

## Background

Modern cancer genomics studies leverage a combination of tissue bulk sampling and genome sequencing [1–3]. This permits the identification of somatic single-nucleotide variants (SNVs), insertions and deletions (indels), copy number alterations (CNAs) [4, 5], driver mutations [6, 7], mutational signatures [8–11], and intra-tumor heterogeneity as part of clonal deconvolution [12–18]. Whole-genome sequencing (WGS) and whole-exome sequencing (WES) have entered the clinic [19], and the number of public databases of tumor genomes is continuously increasing, which presents distinct challenges to cancer genomic analyses. While SNVs have well-established detection tools [4], CNAs, which are a particularly important aspect of the cancer genome [17, 20], are challenging to assess since the baseline ploidy of the tumor (the total chromosome copy number) as well as the percentage of tumor DNA in the assay (i.e., tumor purity), have to be jointly inferred [21–26]. Of particular difficulty is the detection of CNAs occurring in a subset of tumor cells (subclonal CNAs), since this is limited by the current resolution of bulk assays [27] and the available software to infer clonal compositions. While single-cell approaches can identify small sets of cells with shared CNAs, the resolution and quality of that data is still too low to be adopted in the clinic, meaning bulk approaches are favored even if they can only detect “large” subclones [27]. Unless the issue of calling CNAs in the context of variable tumor purity and intra-tumor heterogeneity are overcome, the efficiency and quality of tumor molecular profiles will continue to be affected.

**Fig. 1:**
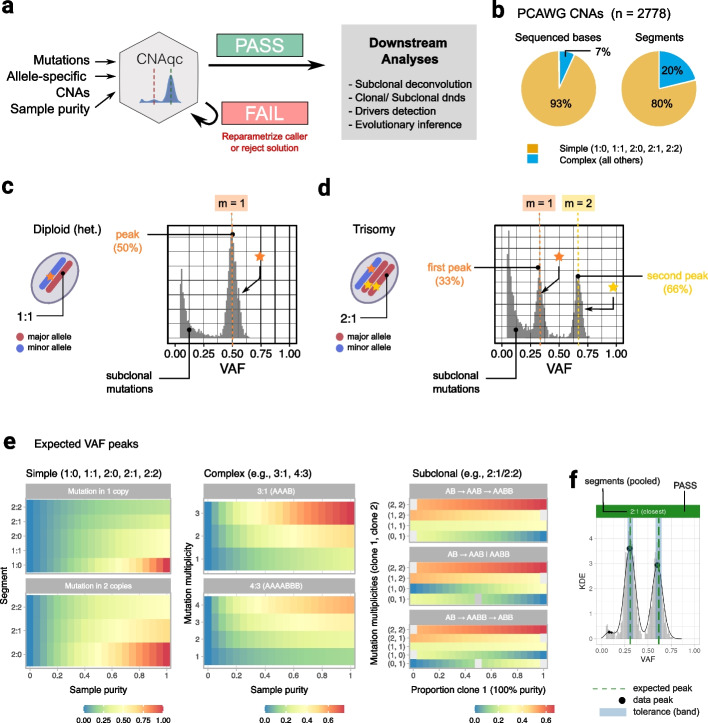
The CNAqc framework for clonal and subclonal CNAs. **a** CNAqc integrates mutations, allele-specific CNAs, and tumor purity $\pi$ to quality control (QC) clonal and subclonal tumor aneuploidy. Given an input purity error tolerance $\epsilon >0$, CNAqc computes a score $\lambda \in \mathfrak{R}$ that leads to a pass ($|\lambda |\le \epsilon$) or fail status. The score magnitude $\lambda$ can be used to (i) adjust $\pi$ and re-parametrise the copy number caller or (ii) to select among alternative ploidy and purity estimates (e.g., a diploid versus a tetraploid solution). **b** CNAqc considers clonal simple CNAs, defined as 1:0 (LOH), 1:1 (heterozygous diploid), 2:0 (copy-neutral LOH), 2:1 (triploid), and 2:2 (tetraploid) states, the most prevalent segments in PCAWG, a large pan-cancer cohort of primary tumors. All other CNAs are called complex. **c,d** VAFs for mutations sitting on 1:1 and 2:1 segments, when $\pi =1$. For 2:1, there are two peaks of clonal mutations (33% and 66% VAF). Multiplicities determine whether a mutation sits on the amplified segment. **e** CNAqc equations predict VAF peaks for any CNA, the distance between data and expected peaks is at the core of our approach. The color scale ranges from 0 to 1 (low to high VAF) for the expected peaks. For complex CNAs, multiplicities range from 1 to the copies of each allele; for subclonal CNAs, peaks depend also on clone size, and the evolutionary (linear/ branching) model of evolution. **f** QC for mutations in a 2:1 segment with ~ 90% sample purity. The vertical dashed lines are expected VAF peaks matched in the shaded area, obtained normalizing $\epsilon$ for segment ploidy, mutation multiplicity, and purity. Black dots are peaks detected by CNAqc; if they fall within the bandwidths, the QC result is pass (green status bar). QC results are computed per peak, per type of segment, and at the sample level

To address the issue of inaccurate CNA calling, we developed CNAqc, the first quantitative framework to integrate somatic mutations, allele-specific CNAs and estimates of tumor purity to quality control (QC) CNA calls generated from WGS and WES assays (Fig. 1a). CNAqc maps SNVs and indels to CNA segments and computes the expected variant allele frequency (VAF) profile based on the particular copy number state called, and tumor purity. Here, the expected VAF of a given mutation varies depending on allele copy state, tumor purity, and clonality, peaking at a theoretical value affected by observational noise [27]. This means that all three genomic features can be assessed simultaneously. We apply CNAqc to several different datasets representing different resolutions and cancer types. These include 2788 WGS samples from the Pan-Cancer Analysis of Whole Genomes (PCAWG) cohort [28], with median coverage 45 × and somatic data generated by more than six tools plus a consensus algorithm, and 235 WGS samples from the Genomics England 100,000 Genomes Project [19], with median coverage 100 × and data generated by Illumina’s DRAGEN latest pipeline (v3). Moreover, we tested our tool on 1464 WES samples from The Cancer Genome Atlas (TCGA) cohort [29] and, finally, with 10 WGS samples from two multi-region colorectal cancers at median coverage 80 × . Results show that CNAqc can achieve excellent performances with little computational costs. Moreover, the tool is flexible to work with data from many different callers, and we find it capable of improving even over pipelines that develop consensus-calling strategies, often adopted in large cohort studies.

## Results

### The CNAqc framework

#### QC of sample purity and copy number segments

CNAqc performs QC of allele-specific CNAs and sample purity estimates prior (Fig. 1a). It adopts different algorithms depending on a classification of the CNA, based on the complexity and clonality of the segment. The complexity involves the number of allelic modifications required to generate a certain CNA from a wildtype diploid heterozygous reference, the clonality captures the proportion of cancer cells harboring a CNA. CNAqc considers simple segments to be diploid heterozygous (1:1), monosomy (1:0), copy-neutral loss of heterozygosity (CNLOH, 2:0), trisomy (2:1), or tetrasomy (2:2), all of which can be acquired through one copy number aberration event (Fig. 1b; Additional file 1: Fig. S1a). These are the most frequently observed CNAs in PCAWG [28] and allow CNAqc to make inferences with precision (simple CNAs in PCAWG: ~ 80% of ~ 600,000 total segments, covering ~ 93% sequenced bases; Additional file 1: Fig. S1b). Simple CNAs are also detectable in bulk sampling datasets, where they also account for the majority of subclonal segments (~ 70% in PCAWG; Additional file 1: Fig. S1c-f). Other less frequent CNAs (3,0, 4:1, 5:2, etc.) are more complex to model and QC, as they are acquired via multiple mutation steps, e.g., to achieve a 3:0 one single-copy gain is required on top of a CNLOH.

Bulk sampling makes the true extent of subclonal CNA heterogeneity difficult to estimate, as only major subclones can be detected with current sequencing resolution. CNAqc supports both clonal or subclonal CNAs, and all QC algorithms were designed using the same logic: given a set of major (${n}_{A}\ge 1$) and minor (${n}_{B}\ge 0$) allele states, with ${n}_{A}\ge {n}_{B}$ and a tumor purity of $0<\pi \le 1,$ and SNVs that overlap the CNAs, the VAF distribution will contain peaks at expected intervals which depend on the percentage of cells harboring the CNA (Fig. 1c,d and Additional file 1: Fig. S2 for two examples). Said differently, CNAqc mathematically links ${n}_{A}$ and ${n}_{B}$ with the VAF of overlapping SNVs given clonality and tumor content. Deviations from expected VAF peaks indicate errors, which can be quantified and used to suggest adjustment of the input parameters or data. It is possible to QC clonal and simple CNAs using this logic and simple algorithms, though subclonal and complex CNAs require more exhaustive assessments because they depend on the percentage of cells associated to a segment, and the evolutionary steps a CNA has taken. Overall, by combining many segments from the tumor genome, CNAqc determines a pass or fail QC result per sample, which can be used to (i) re-parametrise the copy number caller, or to (ii) select among alternative copy number pro file s returned by an algorithm (e.g., a 100% pure diploid tumor versus a 50% pure tetraploid).

The key CNAqc equation (Fig. 1e; Online Methods) predicts a VAF peak for a mutation as: $1\le m\le {n}_{A}$ alleles out of ${n}_{A}+{n}_{B}$ total and is present in a proportion $0<c\le 1$ of tumor cells ($c=1$ for a clonal mutation). The expected peak is a function of the mutation multiplicity $m$ and $c$

1 $${v}_{m}\left(\pi ,c\right)=\frac{mc\pi }{2\left(1-\pi \right)+\pi \left({n}_{A}+{n}_{B}\right)}$$

In real data ${v}_{m}(\pi , c)$ is observed with noise that, for sequencing, is well captured by binomial or beta-binomial distributions [30]. For CNA segments and at least two alleles (${n}_{A}>{1\ge n}_{B}$), the multiplicity $m$ phases mutation mapping on amplified and non-amplified segments. For example, for a 2:1 trisomy segment (${n}_{A}=2$, ${n}_{B}=1$), mutations on the amplified allele have $m=2$ and have been acquired before trisomy. Equation (1) shows that, for a CNA segment, there could be multiple expected VAF peaks as a function of $m$ (Fig. 1d). As presented below, this equation can be generalized for subclonal CNAs, assuming two subclones and given type of evolutionary relationship. CNAqc is the only method we are aware of that can consider both linear and branching evolutionary models: the two subclones B and C emerge linearly (i.e., nested, A $\to$ B $\to$ C) from an unobserved ancestor A, or a branching event (A $\to$ B | C) of a common ancestor (Fig. 1e, Additional file 1: Fig. S3).

CNAqc detects VAF peaks using fast peak detection algorithms (Additional file 1: Fig. S4) that adopt both nonparametric kernel density estimation and binomial mixtures to measure ${v}_{m}(\pi ,c)$ against data. These algorithms compute an error between data peaks and expected peaks and require a threshold $\epsilon >0$ (in Euclidean space) on error magnitude to determine if a peak is matched. To make $\epsilon$ interpretable, however, CNAqc formalizes its link to tumor purity $\pi$ and implements a non-linear error propagation to link $\pi$ with $\epsilon$ (Fig. 1f; Online Methods). This allows the user to input $\epsilon$ in terms of purity units, which are interpretable and intuitive. The overall QC of a bulk sample is determined from all clonal simple CNAs, and a sample score $\lambda \in \mathfrak{R}$ that is a linear combination of the errors accumulated across segments. Because of its interpretability, the score reflects corrections to purity $\pi$ (e.g., $+3\%$, $-7\%$) that are useful for automatic decision making. For example, for heterozygous diploid mutations and 2.5% purity tolerance ($\epsilon =0.025)$ in a sample with $\pi =0.6$ (input purity $60\%$, clonal VAF peak $30\%$), CNAqc will accept data peaks in $[27.5\%;32.5\%]$, and a purity estimate in $[55\%;65\%]$.

#### QC of cancer cell fraction (CCF) estimates

In cancer genomics, detecting CNAs along with estimating sample purity and ploidy is performed as part of cancer cell fraction (CCFs) analyses. Many pipelines that interpret tumor evolutionary trajectories utilize CCFs which, at their core, rely on the estimation of $m$ from the data. CNAqc implements the first algorithm to assess the quality of multiplicity estimates and, in turn, of CCF estimates, from VAFs.

For a mutation with observed VAF $v$, CNAqc defines the CCF (Online methods) by solving Eq. (1) for $c$ [17]

2 $${c}_{m}\left(\pi ,v\right)=\frac{v\left[\left({n}_{A}+{n}_{B}-2\right)\pi +2\right]}{m\pi }$$

Note that the binomial noise affecting $v$ propagates to ${c}_{m}(\pi ,v)$; therefore, for a diploid clonal mutation and pure tumor, the CCF ${c}_{1}(1,v)=2v$ spreads around 1 because $v$ spreads around 0.5. After QC, subclonal deconvolution algorithms can denoise $c$ by clustering. The real challenge to applying Eq. (2) is therefore phasing $m$ from VAFs. For simple CNAs, this restricts to estimating if *m* = 1 or *m* = 2. CNAqc uses a binomial mixture to phase $m$ from VAFs, and the entropy $H(z)$ of the mixture latent variables $z$ to identify a VAF range where $m$ cannot be phased reliably because both *m* = 1 and *m* = 2 seem likely. This information-theoretic approach allows CNAqc to provide a confidence measure over $m$, and therefore ${c}_{m}(\pi ,v)$.

As for general CNA/purity-based QC, a final status (pass or fail) can also be determined for CCFs, which help discriminate for which mutations a CCF score cannot be unequivocally computed from VAFs. For this task, other algorithms are also present to aid CCF identification regardless of entropy (Online Methods).

#### Other features of CNAqc

**Fig. 2:**
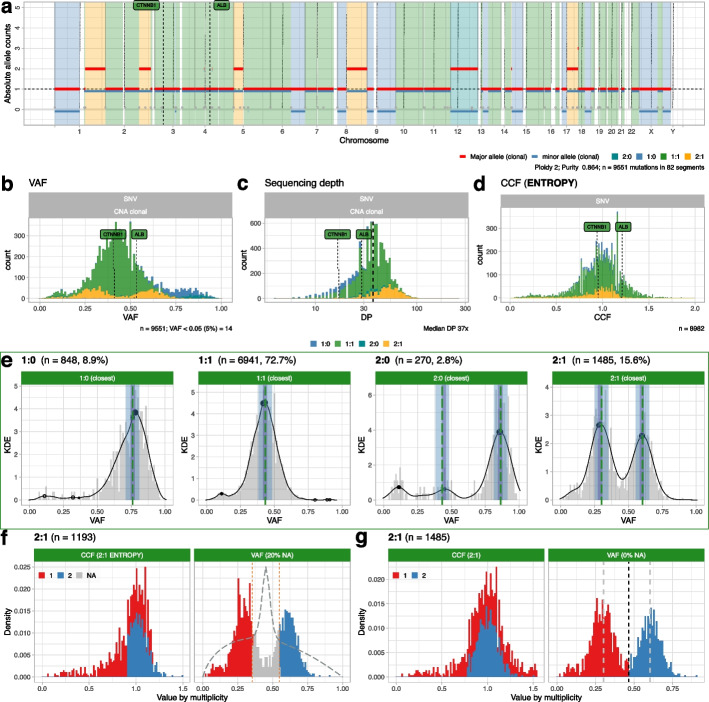
Analysis of a hepatocellular carcinoma sample with PCAWG consensus calls.** a** CNAqc visualization: allele-specific CNAs (ploidy 2, purity ~ 85%) with major and minor allele counts per segment. This sample harbors two driver SNVs hitting genes CTNNB1 and ALB, sitting in diploid heterozygous segments (1:1). **b–d** Read counts for SNVs visualized as variant allele frequencies (VAFs) and depth of sequencing (DP). Cancer cell fractions (CCF) obtained by CNAqc suggest that the two drivers are clonal (CCF spread around 1). **e** Peak detection QC for simple clonal CNAs, as in Fig. 1f. Peaks are checked independently, and the final QC depends on the number of mutations per peak, and whether the peak is matched. The sample-level QC is a linear combination of results from each CNA; here calls are passed (green plot; numbers represent mutational burden). **f,g** CCF estimation for mutations mapping to triploid 2:1 segments, obtained using the entropy-based and the rough methods. CCF values of clonal mutations spread around 1, CCFs and VAFs are colored by mutation multiplicity. The entropy profile (dashed line) delineates crossings of binomial densities where CNAqc detects multiplicity uncertainty from VAFs; the entropy method detects uncertainty in 20% of the SNVs. The alternative method in panel **g** assigns multiplicities regardless of entropy. In both cases, the CNAqc CCF estimates pass QC with default parameters

**Fig. 3:**
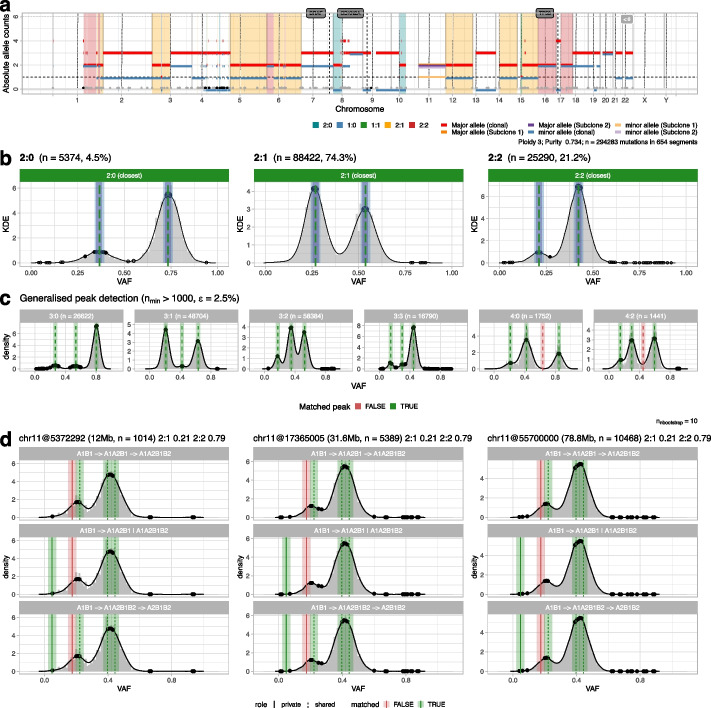
Analysis of a skin melanoma sample with PCAWG consensus calls.** a** CNAqc visualization as in panel **a** of Fig. 2. This sample presents high aneuploidy (mean ploidy 3.69), with most of the genome in triploid 2:1 segments, a very large mutational burden (~ 300,000 mutations) and large subclonal CNAs on chromosome 11. The CNAqc visualization shows the relative subclone proportions as shifts of the *y*-axis for the segment, as in Battemberg [15]. **b** CNAqc validates the calls by peak detection. Note that most of the signal is due to ~ 80,000 mutations mapping to 2:1 segments (~ 75% of total). **c** CNAqc validates 18 out of 20 expected peaks in complex CNAs (3:0, 3:1, 3:2, 3:3, 4:0, and 4:2). **d** CNAqc validates subclonal CNAs on chromosome 11, where two subclones with 2:1 genome (21% of cells) and 2:2 genome (79% of cells) are detected, using both linear and branching models of evolution

CNAqc provides functions to visualize copy number segments, read counts, CCFs, and peak analysis for clonal and subclonal CNAs (Figs. 2 and 3). For example, in Fig. 3c we show results from QC of complex CNAs for a sample with > 3 allele copies, and a large subclonal CNA on chromosome 11, intermixing a 2:1 (21% of cells) and a 2:2 subclone (79% of cells), compatible with both linear and branching evolution. Moreover, the tool contains auxiliary algorithms to smooth segments and detect patterns of over-fragmentation from breakpoints distributions, helping to prioritize additional analysis to determine events of chromothripsis, kataegis or chromoplexy [5, 17].

An important feature of CNAqc is its flexibility and speed. The tool has been designed and tested in a variety of settings and pipelines, also against alternative subclonal deconvolution methods which can also detect peaks from VAF data. Tests with hyper-mutant tumors with thousands of mutations (e.g., Fig. 3) were used to measure the wall-time performance of our method. Notably, CNAqc was able to load and process ~ 500,000 mutations in ~ 60 s (peaks-based QC) on a standard laptop, which was orders of magnitude faster than alternative methods. For example (Additional file 1: Fig. S5), for our range of tests, variational inference and Monte Carlo methods were from 4/16 times to 100 times slower than CNAqc.

### Simulations, single-cell validation and parameters calibration

CNAqc algorithms and parameters were validated by synthetic simulations, and controlled bioinformatics experiments with single-cell data (Online methods).

#### Validation of error metrics and automatic tool parameterization

We used synthetic data to show that the error metrics implemented in CNAqc work as expected for both purity/CNA and CCF QC algorithms, and to parametrise the algorithm to work best considering coverage and purity of the input assay (sequencing parameters).

From ~ 20,000 synthetic tumors with variable coverage (30 × to 120 ×) and known purity (0.4 to 0.95), we ran CNAqc with input purity corrupted by a known error, and observed that the proportion of rejected (fail QC) samples approached 100% when the error exceeded $\epsilon$. These tests showed that the performance of CNAqc is affected by sample purity and simulated coverage (Additional file 1: Fig. S6 and S7). Therefore, from > 350,000 other synthetic tumors, we regressed false positive rates (FPR), i.e., the probability of passing a sample that should fail, against coverage and purity. In this way, CNAqc can suggest, for a desired upper bound on FPR (e.g., maximum 10% of false positives), the best $\epsilon$ considering the coverage and purity of the input dataset (Additional file 1: Fig. S8).

#### Validation of purity adjustments with single-cell data

**Fig. 4:**
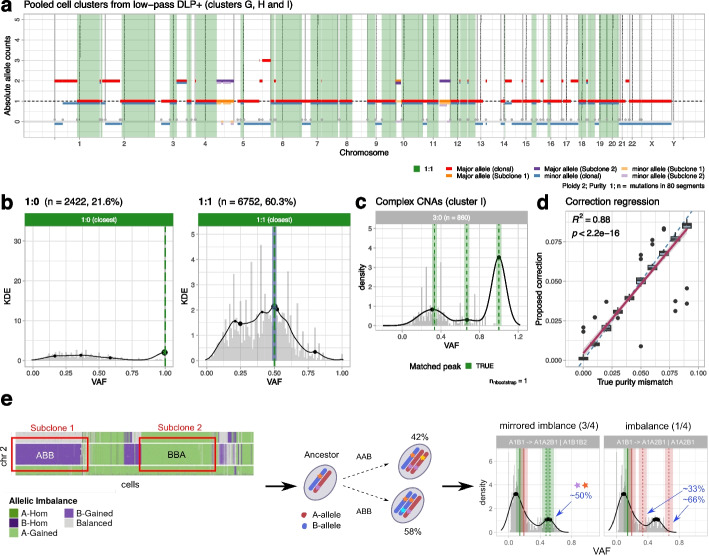
Validation of CNAqc with single-cell low-pass DNA sequencing. **a** Pseudo-bulk CNAs from single-cell low-pass data of an ovarian cell line [31]. This profile is obtained from pooling CNAs across several diploid tumor subclones. This artificial bulk with known ground truth is then used to validate CNAqc (Additional File 1: Fig. S9). **b** QC of LOH and diploid heterozygous segments from panel **a**, using true tumor purity (100%). **c** Quality control of complex 3:0 segments (cluster I; Additional File 1: Fig. S9). **d** Correlation between purity adjustments suggested by CNAqc and errors created artificially for single-cell data. By construction, the desired correction sits on the diagonal because the true purity is 100%; the tool achieves ${R}^{2}=0.88$, correlation test *p*-value $p<1{0}^{-16}$. **e** Advanced QC of subclonal CNAs from admixing of tumor subclones with mirrored allelic imbalance, as originally detected in [32]. In this test, two triploid subclones with mirrored alleles (AAB versus ABB) are admixed. CNAqc can validates these calls and identify the true branching patterns of evolution (AB $\to$ AAB | ABB), which is characterized by a peak of shared mutations at around 50% VAF

We artificially created pseudo-bulk datasets from single-cell datasets with associated low-pass data (Fig. 4a) and used that to validate purity-adjustment metrics implemented in CNAqc.

We collected mutations and CNAs from single-cell low-pass whole-genome data of ovarian cancer cell lines [31, 33]. From 3 tumor clones with distinct CNAs (Additional file 1: Fig. S9), we created a pseudo-bulk dataset and consensus clone-level CNAs (Online Methods), which we validated using CNAqc with true 100% purity (Fig. 4b,c, Additional file 1: Fig. S10). We then pooled clonal segments across the 3 subclones to create a larger population and observed that the purity adjustment proposed by CNAqc with miscalled input purity follows $\epsilon$ linearly ($0.88\le {R}^{2}\le 0.99$; $p<1{0}^{-16}$; Fig. 4d, Additional file 1: Fig. S11).

#### Validation of subclonal CNAs and CCFs

CNAqc validated subclonal CNAs and CCFs in artificial datasets from mixed single-cell data and real data, showing its ability to retrieve the tumor subclonal evolution and implement QC accordingly.

We validated two subclones with trisomy and tetrasomy artificially mixed from low-pass data (Additional file 1: Fig. S12). Then, using 10 × data [32] and a pseudo-bulk mixture of 2 subclones with a trisomy and mirrored allelic imbalance (Additional file 1: Fig. S13), we tested the evolution-based QC of complex subclonal CNAs. This test was particularly interesting because the two clones have the same segment ploidy (3), but the joint presence of AAB and ABB genotypes (mirrored allelic imbalance) can only be explained by branching from an AB ancestor (Online Methods). CNAqc validated these subclonal CNAs identifying the expected AB $\to$ AAB | ABB model for the clones (Fig. 4e, Additional file 1: Fig. S14).

Moreover, we computed CCFs from VAFs in pseudo-bulks and, from a cluster of cells with a triploid amplification, CNAqc did flag as uncertain the same mutations for which we could not compute multiplicity from single-cell data (Additional file 1: Fig. S15). Finally, we compared CCFs computed by CNAqc to standard subclonal deconvolution tools. On real data, CCFs by CNAqc were consistent with standard methods (Additional file 1: Fig. S16) but, importantly, the uncertainty metrics from CNAqc did identify spurious subclonal clusters explained by miscalled CCFs, showing the importance of using QC metrics to avoid propagating errors in downstream analyses (Additional file 1: Fig. S17).

### Large-scale WGS pan-cancer PCAWG calls

The PCAWG cohort ($n=2778$ samples, 40 tumor types) contains WGS samples at median depth 45 × and purity ~ 65%, comparable to our simulations. This cohort comes with copy number and mutation data generated from 6 state-of-the-art algorithms, as part of a curated consensus [16]. Excluding samples with lack of data or too few mutations (Methods), we ran CNAqc on 2589 samples in less than 1 h with a standard computer, confirming the speed of our QC.

**Fig. 5:**
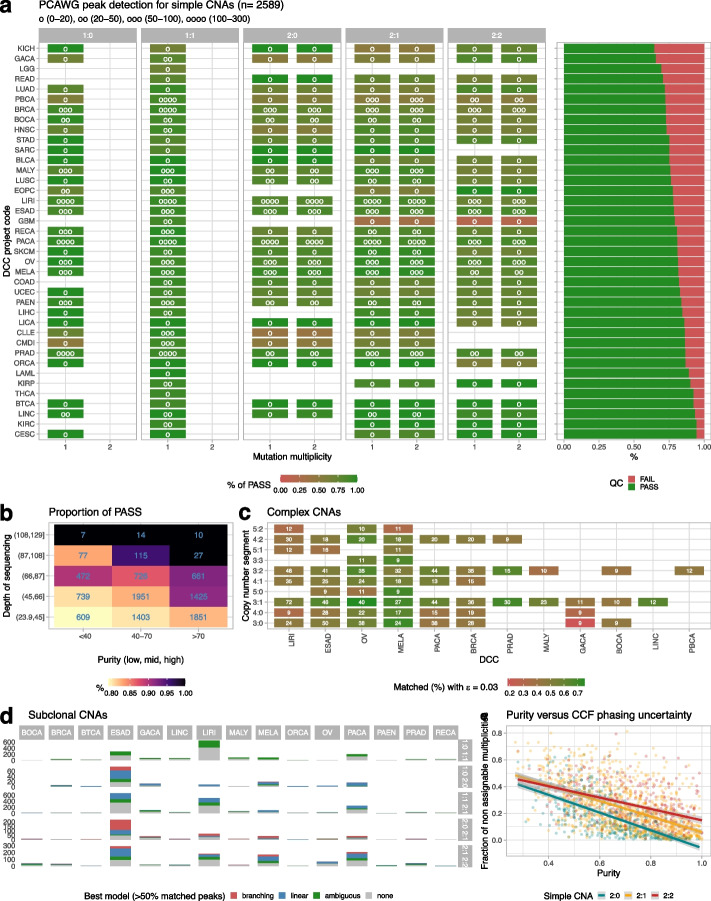
Analysis of the PCAWG consensus calls with CNAqc.** a** QC for simple CNAs in $n=2589$ PCAWG samples (median coverage 45 ×), using consensus CNAs and mutation calls. The plot shows the percentage of cases with pass status, split by segment type, multiplicity, and tumor type, with sample size shown with the asterisks. **b** Proportion of cases that pass QC, split by purity (low, mid and high) and median depth of sequencing (removing outliers with depth < 24 × or > 129 ×). **c** QC for $n=570$ PCAWG samples with complex CNAs and > 150 mutations per segment, with tumor types ranked by number of cases. Numbers report the absolute number of cases. **d** Best evolution model used to analyze $n=538$ PCAWG samples with subclonal CNAs and > 150 mutations per segment, with tumor types ranked by number of cases.** e** Regression of tumor purity against the proportion of segments with unassigned CCF values using the entropy method in CNAqc

Overall PCAWG consensus calls for clonal simple CNAs (*n* = X segments) were passed in 2339 out of 2589 samples (~ 90%) with 3% error purity tolerance ($\epsilon =0.03$), confirming the quality of the consensus copy number data (Fig. 5a). As with simulations, the QC pass rate was determined by tumor purity and depth of coverage (Fig. 5b). We observed paradigmatic examples with low mutational burden that failed QC (Additional file 1: Fig. S18), or rare cases with excessively high purity (~ 100%) that, upon re-analysis, were better fit with very low tumor content (Additional file 1: Fig. S19). Similarly, we validated cases with very high purity > 95% (Additional file 1: Fig. S20). We also examined complex clonal CNA segments with at least 150 mutations (610 samples from esophageal, liver, melanoma, ovarian, pancreatic and breast cancers; Fig. 5c). The most prevalent CNAs were 3:1, 3:2, and 3:0 (15, 13, and 10%), for which CNAqc matched > 60% of peaks on average (Online Methods).

We applied CNAqc to 538 cases with subclonal CNAs called by Battenberg (Fig. 5d) with at least 150 mutations per segment, which were found mostly across esophageal, liver, melanoma, pancreatic, and gastric cancers. Interestingly, some of these tumor types also carried complex CNAs, suggesting pervasive chromosomal instability. The most frequent clonal compositions (listed as clone 1–clone 2) were 1:0–1:1 (~ 33%), 1:1–2:1 (~ 31%); 2:1–2:2 (19%), 2:0–2:1 (~ 9%), and 1:0–2:0 (~ 7% of cases). For each segment, we determined the best fitting model by assessing the percentage of matched peaks, avoiding assigning a model if less than 50% of the expected peaks were matched. Overall, CNAqc assigned a model to ~ 87% of the subclonal CNAs (Additional file 1: Fig. S21 and S22). Interestingly, subclones 1:0–2:0, 1:1–2:1, and 2:1–2:2 were generally better explained by linear (A $\to$ B $\to$ C) evolution implying a temporal ordering among the subclones (in 39, 48, and 52% of cases, including for subclones 2:1–2:2 in triploid 2:1 or tetraploid 2:2 tumors). This inference can be explained biologically. For example, 1:0–2:0 subclones could emerge as a CNLOH gain (2:0), after a loss (1:0) from a diploid ancestor (1:1). Similarly, 2:1–2:2 subclones can follow a linear amplification path where alleles are progressively gained over time. Conversely, subclones 2:0–2:1 were better explained by branching models (38%), implying the independent formation from a common ancestor. This is intuitive, because, for instance, the evolution from CNLOH to trisomy cannot be linear. In general, these statistics also reflected in tumor types, with 2:0–2:1 subclones in esophageal adenocarcinomas explained by both models, while 2:1–2:2 subclones are better explained by linear models for liver and pancreatic cancers, and melanoma.

Finally, we computed CCFs on the entire PCAWG cohort. As with our simulations, the percentage of unassigned CCFs negatively correlated with sample purity (Fig. 5e). The CCFs produced by CNAqc (Additional file 1: Fig. S16) were comparable to those computed by Ccube, the official PCAWG tool to compute CCFs [34]. Comparing CCFs and peak-based analyses, we could conclude that, while peaks could be detected for all PCAWG samples, multiplicity phasing would have required higher coverage and purity to reduce uncertainty.

### High-resolution WGS calls at Genomics England

The Cancer Programme of the 100,000 Genomes Project was a transformational UK government project designed to incorporate WGS into NHS clinical service. Genomics England, in partnership with NHS England, generated whole-genome analysis for over 16,000 fresh frozen tumor samples, with a median coverage of 100 × . These data provide an ideal retrospective test set for CNAqc, which is now being routinely applied in the validation process of the clinically accredited bioinformatics pipeline at Genomics England.

We gathered a subset of the WGS data (*n* = 235 samples from [35]) with mutation and CNA calls generated by the Illumina DRAGEN (Dynamic Read Analysis for GENomics; > v3.9) platform [36]. These tumors split into groups of distinct subtypes, with the largest groups being pediatric tumors (PT, *n* = 17), acute lymphoblastic leukemia (ALL, *n* = 104), acute myeloid leukemia (LAML, *n* = 29), breast cancers (BRCA, *n* = 41) and sarcomas (SARC, *n* = 44). This test is of particular importance because these samples are used to optimize the CNA calling pipelines implemented at Genomics England, which serve both clinical reporting and research.

**Fig. 6:**
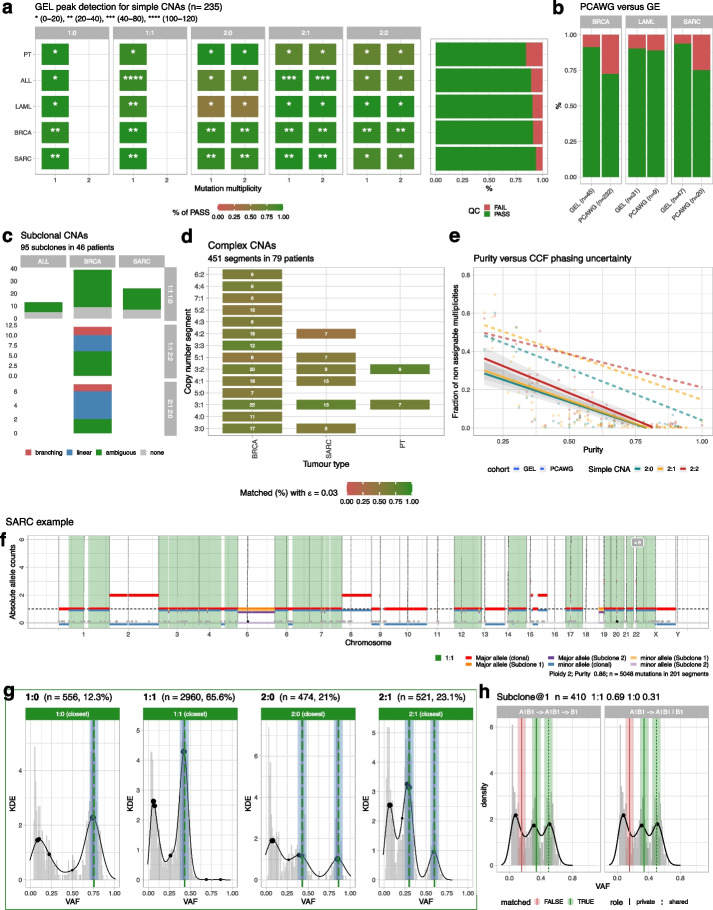
Analysis of the Genomics England DRAGEN calls with CNAqc.** a** QC from simple CNAs in $n=235$ Genomics England samples (median coverage 100 ×), using DRAGEN data. The plot is like panel **a** in Fig. 5 for PCAWG. **b** Proportion of cases that pass QC, comparing Genomics England and PCAWG, for three tumor types: BRCA (breast cancer), LAML (acute myeloid leukemia), and SARC (sarcoma). **c–e** QC for subclonal CNAs in ALL (acute lymphocytic leukemia), BRCA, and SARC (**c**), complex CNAs in BRCA, SARC, and PT (pediatric tumors) (**d**), and proportion of segments with unassigned CCF values against purity. Plots are as in panels **c–e** in Fig. 5 for PCAWG. **f–h** Example QC of clonal simple (**g**) and subclonal CNAs (**h**) for a SARC sample with associated segmentation (**f**), where DRAGEN detects aneuploidy as well as a subclonal CNA involving an LOH event associated with 410 distinct mutations (in 31% of the tumor cells). These estimates are validated by CNAqc which detects, in the data, VAF peaks

Results from CNAqc (run using the same parameters as for PCAWG) show a high-quality variant call set with pass rates in all cancer types above 90% (Fig. 6a). In comparison, in lower-coverage PCAWG data some tumor types reached only ~ 70% pass rate (even if consensus calling was used). For tumor types with large numbers of samples and CNAs, the pass rates for Genomics England data with variant calling done by DRAGEN are much higher than the PCAWG consensus (Fig. 6b). For instance, for breast tumors (BRCA), we achieved a pass rate of > 95% (*n* = 45 segments) with the Genomics England dataset whereas in PCAWG only ~ 75% of segments passed QC (*n* = 232 segments). Similarly, for sarcoma tumors (SARC), we achieved a pass rate of > 90% (*n* = 47 segments) with the Genomics England dataset whereas in PCAWG only ~ 75% (*n* = 20 segments). In general, with 100 × data we could also achieve a high pass rate for subclonal CNAs (Fig. 6c), as well as complex clonal CNAs (Fig. 6d). In the case of breast cancers, we could identify subclonal LOH events validated in 35 out of 36 cases, and the same happened for 32 cases among sarcomas. Overall, all the QC computations reported higher success rates with the Genomics England dataset as compared to PCAWG (example fits in Fig. 6g,h). This trend was confirmed also when we computed CCFs, where we reached ~ 15% of unknown estimates when tumor purity in Genomics England samples was ~ 50% while in the PCAWG dataset at ~ 50% purity ~ 40% of the mutations could not be assigned a reliable CCF. The increased coverage in the Genomics England cohort allowed better estimates of tumor CNAs and tumor purity, providing a strong motivation for considering the depth of coverage of a sequencing assay as a key aspect when designing specific analyses.

### Large-scale WES pan-cancer TCGA calls

The statistical signals used by CNAqc are spread through the whole tumor genome, but many assays are limited to sequence, upon capture, only the whole-exome (WES). We used data from TCGA to show (i) that the performance of CNAqc is robust also with WES data and that (ii) consensus procedures for purity estimation were imprecise in many TCGA samples.

First, we collected data for $n=48$ lung TCGA adenocarcinomas [29], a tumor type with high aneuploidy, for which the sample purity and segments are available from popular bioinformatic tools (ESTIMATE [37], LUMP [38], and ABSOLUTE [39]), as well from a TCGA consensus purity estimation (CPE) obtained by immunohistochemistry analysis and the joined tools. We selected the lowest and highest-purity cases to capture different levels of data quality (Online Methods) and applied CNAqc successfully to rank fits from all callers (Additional file 1: Fig. S23). Strikingly, we found some cases where CNAqc failed all purity estimates, including CPE, but passed the one by ABSOLUTE (see Additional file 1: Fig. S24 for an example with 80% CPE, failed, and 69% ABSOLUTE, passed), which is known to use a mutation-based heuristic to improve fits quality (Methods).

To investigate the frequency of this type of errors, we extended this test to $n=1464$ TCGA samples from multiple tumor types, retaining cases with at least 200 mutations (Online Methods; Additional file 1: Fig. S25 and S26). We generalized our finding with 901 cases (60% of 1464) where CPE purity was failed by CNAqc, while the purity proposed by ABSOLUTE often passed QC. We assessed that, had we used CNAqc to select the best purity instead of consensus, 785 out of 901 cases (~ 88%) would have passed QC, obtaining a purity estimate more precise than the TCGA consensus. This shows convincingly that CNAqc can recover a good purity/CNA estimate, even when a consensus approach is invariably confused, i.e., most consensus inputs misscall purity.

### QC-powered automatic copy number calling pipeline

We used CNAqc to assemble the first automatic copy number calling pipeline (Additional file 1: Fig. S27) to iterate CNA calling until QC is passed (or for a maximum number of steps). This pipeline leverages the Sequenza clonal CNA caller [22] and combines CNAqc purity-adjustment scores together with ranking of alternative solutions determined by the Sequenza algorithm. By the generality of CNAqc, this approach could be extended trivially to other CNA calling algorithms.

We used the Sequenza-CNAqc pipeline to generate clonal copy number data for two colorectal cancer patients with multi-region WGS data associated. From a total of 10 samples with median coverage ~ 80 × and purity ~ 80% (Additional file 1: Fig. S29), the pipeline automatically generated calls that passed QC. Moreover, the tool was also able to identify the true CNA profile when artificially miscalled copy number profiles were given in input.

## Discussion

Cancer precision medicine, boosted by the large-scale adoption of bulk sequencing in the clinic, will increasingly rely on landmark cancer genomics programs [19, 28, 40]. This poses data quality under the spotlight, advising against manual curation and consensus approaches that either do not scale or bring substantial bioinformatic overheads. Therefore, automatic procedures to quality control (QC) mutation calling pipelines are highly desired [4, 41, 42].

To the best of our knowledge, CNAqc is the first framework to formalize QC algorithms for bulk assay, leveraging SNVs and indel mutations, along with allele-specific CNAs, tumor purity, and estimations of clonality. CNAqc can be used to process and QC the most common CNAs found in human cancers, using distinct algorithms based on clonality and the complexity of CNAs under scrutiny. In particular, CNAqc can delineate the evolutionary history of subclonal CNAs and quantify the likelihoods of the underlying evolutionary processes, implementing a QC algorithm inspired by tumor evolution principles. The evolutionary model implemented is however simple, and in future releases, it could be improved to support multiple observations from the same tumor (multi-region data), or longitudinal data. Importantly, all algorithms presented in our framework have been validated in controlled single-cell experiments and synthetic simulations. CNAqc can also support downstream analysis using innovative algorithms. In fact, CNAqc is the first model to compute per-mutation CCFs within an information-theoretic uncertainty model for the estimation of mutation multiplicities. This is biologically very relevant for downstream analyses that rely on CCFs, which are at the cornerstone of all copy number timing [17] and tumor evolutionary inference analyses [12–18].

CNAqc was used to analyze bulk WGS and WES data from TCGA, PCAWG, and Genomics England. Notably, this included copy number and mutation data generated from more than 10 widely used bioinformatic pipelines, as well as with consensus calls in TCGA and PCAWG. Our analysis proved that sequencing coverage impacts the rate of successful copy number calling, with a clear advantage observed when comparing ~ 100 × WGS from Genomics England against earlier PCAWG cohorts, both in terms of clonal and subclonal copy number detection, as well as CCF computation. Particularly interesting results emerged comparing consensus approaches that combine predictions of distinct algorithms. Consensus-based methods are popular in bioinformatics and rely on the assumption that by gathering data from many tools we can improve the quality of our predictions. While these strategies have obvious computational overhead, the sensitivity of such procedures is largely dependent on the quality of its input methods. Strikingly, we found that in ~ 60% of TCGA samples (from a scrutiny of ~ 1500 samples), the consensus was not robust to confounders impacting individual tools, and CNAqc was required to identify and fix imprecise consensus purity estimates. In general, the possibility that QC-based methods could substitute or at least augment consensus approaches would make somatic calling pipelines faster and simpler to organize or maintain. In this respect, we released the first automatic CNA calling pipeline that joins a popular copy number calling algorithm with CNAqc.

The quality of QC by CNAqc depends on the capacity to detect VAF peaks, which creates issues when a sample has low purity (e.g., below 20%), low coverage (e.g., below 30 ×), low mutational burden or when there is significant tumor contamination in a germline sample [43, 44]. Most of these limitations are “technological” and can be overcome with higher depth of sequencing and upfront assessment of tumor content. That said, there is an intrinsic limit to how well bulk sequencing designs can capture subclonal copy numbers, especially at the scale of single cells [45–48]. This limitation of bulk assays may place focus on alternative emerging technologies such as tissue laser capture, though in the short to long term this challenge does not reduce the importance of assessing data quality from widespread bulk sampling.

## Conclusions

Generating high-quality copy numbers and mutation data is a necessity for successfully interpreting cancer genomes [12–14, 17, 49–56]. CNAqc can help to assess whether the quality of the sequencing data is sufficient to ask specific research questions related to tumor aneuploidy, evolution, and general molecular profiling.

With the ongoing implementation of large-scale sequencing efforts, CNAqc offers a modular solution to augment established pipelines, aiding the self-tuning of bioinformatics parameters based on quality scores. To our knowledge, this is the first stand-alone tool which combines a tumor-evolution perspective with common types of cancer mutations to automatically control the quality of a sequencing assay.

## Methods

### CNAqc

#### Taxonomy

CNAqc supports the two most popular human reference genome assemblies GRCh38 and hg19 and distinguishes between different types of allele-specific CNAs:

- “Simple clonal CNAs”: clonal segments including states of heterozygous diploid (AB or 1:1), monosomy loss of heterozygosity (LOH) (A or 1:0), copy-neutral loss of heterozygosity (LOH) (AA or 2:0), triploid amplification (AAB or 2:1), and tetraploid amplification (AABB or 2:2);
- “Complex clonal CNAs”: clonal segments with any combination of major and minor allele copies (e.g., 3:2, 4:0, 6:1). These are complex because, in the most general case, they are acquired with multiple subsequent events of acquisition or loss of alleles, from a baseline wildtype 1:1 state (normal);
- “Subclonal simple CNAs”: simple segments in 2 subclones, with known proportions.

This taxonomy is inspired by the frequency that these events have in the PCAWG cohort (Additional file 1: Fig. S1a.), where 36% of clonal CNAs are 1:1, 15% are 2:1, 11% are 1:0, 8% are 2:2, and 8% are 2:0. Note that for sex chromosomes we expect, in absence of aneuploidy, chromosome X to be 1:1 for females and 1:0 for males. Simple CNAs in PCAWG are > 75% of the whole set of > 600,000 segments, and span 93% of all CNA-covered bases (Additional file 1: Fig. S1b.). In the same cohort, most CNAs are clonal, and on average, simple CNAs cover ~ 80% of the overall genome per sample (Additional file 1: Fig. S1c-d.). At the subclonal level, Battenberg segments with simple CNAs are ~ 70% of the overall subclonal ones (Additional file 1: Fig. S1e-f.). We note that the CNAqc limitation of handling 2 subclones is consistent with the capacity of popular subclonal callers such as Battenberg, ReMixT, and CloneHD, which can only detect these configurations of subclonal copy numbers [15, 26, 57].

These assumptions serve to limit the computational complexity of the problems approached in CNAqc [27]. Simple CNAs are evolutionarily acquired in one step from a heterozygous germline diploid state. For instance, from a wildtype 1:1 a tumor cell might either amplify one allele and reach a 2:1 trisomy, or lose an allele and reach a 1:0 LOH. With slightly more complex biological mechanisms, states such as 2:0 or 2:2 can also be reached in a single evolutionary step. Complex CNAs require instead articulate modelling; for instance, a 4:0 might be the final endpoint of a tumor undergoing whole-genome doubling, then further amplifying to 4:2 and eventually reaching 4:0. CNAqc attempts some degree of evolutionary inference at the level of subclonal CNAs, where it uses simple CNAs to determine whether the subclones have evolved linearly or branching out of a common ancestor. For instance, if the subclones are 1:0 and 2:0, CNAqc will try to determine whether the tumor branched from 1:1 with 1:0 and 2:0 siblings 0 (1:1 → 1:0 | 2:0), or if the tumor jumped from 1:1 to 1:0 and then from 1:0 to 2:0 0 (1:1 → 1:0 → 2:0), modelling 1:0 and 2:0 as nested, or vice versa (1:1 → 2:0 → 1:0). In this formulation, there is also some degree of simplification, at least as far as what CNAs are supported to make reasonable inferences.

CNAqc is conceptualized to work with high-resolution—i.e., high purity and coverage—whole-genome and whole-exome sequencing (WGS) data (see “Analysis of patient data”). For clonal CNAs, the method pools mutations from segments with the same copy numbers (e.g., all 2:1 segments), either across the whole genome or per chromosome. Subclonal segments are instead analyzed without any pooling to support algorithms (e.g., Battenberg) that compute segment-specific CCFs. CNAqc uses the signal of mutations to analyze the data; therefore, a challenge with WES or low-resolution WGS data is the reduced mutational burden and noise in the VAF, which decreases the signal strength. The key determinant to detect VAF peaks is therefore the number of mutations per copy state, which can be affected by genomic or microsatellite instability, or by the presence of endogenous mutant factors such as smoking or UV light. These conditions can facilitate the application of WES data. At the level of subclonal CNAs, the problem is also present since the number of mutations per segment is smaller compared to the clonal counterpart.

In general low-purity or low-coverage data impact the QC performance with false positives and negatives rates. In terms of how this impacts the automatization of the QC step, in our best practices we randomly check samples if the median coverage is lower than 50 × and the purity is below 50%. These cutoffs have been determined by cross-referencing simulation results, as well as by the large-scale application of our tool to both WGS and WES data.

### Simple clonal CNAs

#### Expected VAF peaks for clonal CNAs

A bulk is a mixture of tumor and normal cells present in proportion $\pi >0$ and $(1-\pi )$, respectively. Each mutation is present in a percentage $0<c\le 1$ of tumor cells, the cancer cell fraction (CCF) of the mutation; to introduce our framework, we consider clonal mutations (with $c=1$). We use a model to describe the position of a clonal VAF peak in the data, assuming the input clonal segments and purity are correct. This equation links all segments with the same allele-specific CNA.

In this manuscript, we denote ${n}_{A}:{n}_{B}$ segments with ${n}_{A}$ and ${n}_{B}$ copies of the major and minor alleles (e.g., 1:1 has ${n}_{A}={n}_{B}=1$; 1:0 has ${n}_{A}=1,{ n}_{B}=0$). With $m\ge 1$ we denote the multiplicity (or copies) of a mutation in the tumor genome: for simple CNAs $1\le m\le 2$, whereas for complex $1\le m\le {n}_{B}\le {n}_{A}$. As in ASCAT [21], $m\pi$ is the expected proportion of reads attributed to a mutation with multiplicity $m$. However, while ASCAT uses germline single-nucleotide polymorphisms (SNPs), our approach uses somatic mutations. While the conceptualization is similar and already appears in [27] for reasons other than QC, using mutations has two advantages. First, VAFs are orthogonal to B-allele frequencies (BAFs) often used by CNA callers. Second, certain genome configurations cannot be detected from BAFs while they can be clearly detected from VAFs. As an example, consider a whole-genome duplication (WGD) where the BAF is the same as a 1:1 heterozygous diploid state because WGD duplicates both A and B alleles (see below). Conversely, the VAF distribution is bimodal, with one mode for mutations accumulated before WGD (e.g., in a 1:1), and one for those subsequent to WGD.

For segments ${n}_{A}:{n}_{B}$, the proportion of all tumor reads is ${\pi (n}_{A}+{n}_{B})$, where ${n}_{A}+{n}_{B}$ is the ploidy of the segments (to be distinguished from the ploidy of the overall tumor). For a healthy diploid normal and tumor clonal mutations sitting on ${n}_{A}:{n}_{B}$ segments,

1 $${v}_{m}(\pi ,c)=m\pi c/[2(1-\pi )+\pi ({n}_{A}+{n}_{B})]$$

is our expectation for VAF peak. To simplify notation, we sometimes use ${v}_{m}(\pi )$ to represent peaks of clonal mutations (where $c=1$). Equation (1), which we use below to compute also CCFs, describes our belief about VAF peaks assuming the segmentation and purity $\pi$ are correct, and “phasing” mutations on the amplified or non-amplified alleles via $m$. As an example, for $\pi =1$ and a clonal heterozygous diploid segment ($1={n}_{A}={n}_{B}$), since $m=1$ then VAF peaks at ${v}_{m}(1)=0.5$. Instead, for WGD ($2={n}_{A}={n}_{B}$), clonal mutations in single ($m=1$) or double ($m=2$) copy give ${v}_{1}(1)=0.25$ and ${v}_{2}(1)=0.5$. We note that Eq. (1) holds for both SNVs and indels but, as best practice, we often apply it to SNVs to avoid noisy VAF measurements of indels, which are prone to well-known alignment issues.

#### Peaks detection algorithm (Additional file 1: Fig. S4)

CNAqc implements a kernel-based and a mixture-based strategy to detect VAF peaks. The former first smooths VAFs via kernel density estimation with fixed bandwidth, and then uses external R packages for peak detection to determine density peaks above 1/20 (empirical cut) of the maximum observed peak. The latter uses a finite Dirichlet mixture with binomial likelihood, as implemented in the BMix [30] package (https://caravagn.github.io/BMix/). The latter strategy is inspired by subclonal deconvolution methods, and computes the density for $w$ clusters (default $w<5$), with model-selection to optimize $w$ using the Integrated Classification Likelihood score [30]; the likelihood is

1.1 $$f\left(X\left|\pi ,p\right.\right)=\prod_{x\in X}\sum_{i=1}^{w}{\pi }_{i}Bin\left({r}_{x}\left|{n}_{x},{p}_{i}\right.\right)$$

where ${\pi }_{i}$ are the mixing proportions of the mixture (not to be confused with sample purity). Here we use a binomial likelihood for ${r}_{x}$ successes determined as the number of reads with the mutant covering mutation $x$, ${n}_{x}$ is the total trials given by sequencing depth at the locus, and ${p}_{i}$ the binomial probability. If data was perfect, ${p}_{i}$ should match expected VAF peaks from Eq. (1). A key advantage of BMix over other deconvolution tools is the fast maximum likelihood implementation, with full access to the model parameters.

#### Peak-based score metrics

The peak-matching algorithm (Additional file 1: Fig. S4) pools at the genome or chromosome-level all mutations from segments with the same ${n}_{A}:{n}_{B}$. As output, it determines a score per-mutation multiplicity, which is propagated to the sample with a linear combination weighted by the number of mutations in a segment. Note that by mapping scores along the genome, CNAqc can QC even specific portions of the tumor genome. The scores have a sign to reflect purity adjustments to fit Eq. (1) better, and the sample-level score is compared with a user-defined $\epsilon >0$ to determine an overall pass or fail status. $\epsilon$ is in units of sample purity and represents the maximum error we tolerate. For example, for $\epsilon =0.05$, if the true purity was 60%, CNAqc would pass estimates in [55%, 65%], and fail others. Mathematically, the range associated to $\epsilon$, as well as $\epsilon$ itself are adjusted to account for ploidy and mutation multiplicity, converting error measures from VAFs to purity units (see below).

In the most general formulation we detect $n$ peaks ${d}_{1},...,{d}_{n}$ from VAF, which we need to match to peaks predicted by Eq. (1). The mapping strategy is subject to some degree of freedom, and in CNAqc we decided to match every expected peak ${v}_{m}(\pi )$ to a data-peak ${d}_{*}$ by minimizing the geometric distance ${d}_{*}={arg}\mathsf{m}\mathsf{i}{\mathsf{n}}_{D} |{d}_{i}-{v}_{m}(\pi )|$ where ${d}_{i}\in D$. The choice of the peaks to consider (set $D$) has two options: we might either consider all data peaks (i.e., ${D=\{d}_{1},...,{d}_{n}\}$), or only peaks to the right of ${v}_{m}(\pi )$ (i.e., $D=\{{d}_{i}>{v}_{m}(\pi )| i =1,..,n\}$)*.* The most general and default strategy is the first, but the second is of particular help when we search for miscalled breakpoints. For instance, if the caller has returned a diploid segment that is actually stretching over a miscalled LOH segment, then the second strategy will detect the VAF peak of the miscalled LOH and inform us of the error.

Whatever is the peak-matching strategy, distances in VAF space need to be propagated to purity space, because the input tolerance $\epsilon$ is in units of purity. This VAF-purity mapping depends on the particular CNA segment we consider, and mutation multiplicity. Considering clonal mutations ($c=1$), we are interested in the error propagation, in VAF space, on expected peaks given an expected error $\epsilon$ of the input purity. In order to do this, we have to linearize the function ${v}_{m}(\pi )$ that maps purity $\pi$ to the expected peaks in VAF space. This is achieved by performing a first-order Taylor expansion of the finite increment $\Delta {v}_{m}(\pi ,\epsilon ) = {v}_{m}(\pi + \epsilon )-{v}_{m}(\pi )$, which requires to compute the first derivative of ${v}_{m}(\pi )$ with respect to $\pi$. We obtain, by denoting $p={n}_{A}+{n}_{B}$, the following

1.2 $$\Delta {v}_{m}(\pi ,\epsilon ) \approx \frac{\partial {v}_{m}}{\partial \pi }\epsilon =2m\epsilon /\{[2(1-\pi )+\pi p{]}^{2}\}$$

This means that, for a given purity error $\epsilon$, the error on VAFs $\Delta {v}_{m}(\cdot )$ depends on $\pi$ and $m$. Consider, for instance, a 2:1 segment for a tumor with purity 90%, $\epsilon =0.05$ (5%) corresponds to an error in the VAF of approximately $\Delta {v}_{1}(\pi =0.9, \epsilon =0.05) = 0.01 (1\%)$ and $\Delta {v}_{2}(\pi =0.9,\epsilon = 0.05) = 0.02 (2\%)$ for the VAF peaks with multiplicity $m=\{\mathrm{1,2}\}$ respectively.

By inverting Eq. (1), one can express the purity as a function of the VAF, ploidy, and multiplicity and derive the error propagation formula from the VAF to the purity space. Using the same approach as above, we can treat the purity as a function of the VAF by inverting Eq. (1) with respect to $\pi$ and setting $c=1$. We treat now ${v}_{m}$ as a variable, and $\pi$ as a function of ${v}_{m}$ for a given *m*. The formula for $\pi$ is

1.3 $$\pi ({v}_{m})=2{v}_{m}/[m+(2-p){v}_{m}]$$

Then, to derive a purity variation function $\Delta \pi ({v}_{m},\Delta {v}_{m})=\pi ({v}_{m}+\Delta {v}_{m})-\pi ({v}_{m})$, we assume an error $\Delta {v}_{m}$ on peaks position and truncate the Taylor expansion of ${\pi (v}_{m})$ at the first order to get the error propagation formula

1.4 $$\Delta \pi ({v}_{m},\Delta {v}_{m})\approx \frac{\partial \pi }{\partial {v}_{m}}\Delta {v}_{m}=2m\Delta {v}_{m}/[m+{v}_{m}(2-p){]}^{2}$$

This makes sense, and If we replace in this formula Eq. (1) and (1.2) of ${v}_{m}$ and $\Delta {v}_{m}$ we obtain the original error $\epsilon$ in purity space

1.5 $$\frac{2m\Delta {v}_{m}}{[m+{v}_{m}(2-p){]}^{2}}=\frac{2m\frac{2m\epsilon }{[2(1-\pi )+\pi p{]}^{2}}}{[m+\frac{m\pi (2-p)}{2(1-\pi )+\pi p}{]}^{2}}=\frac{4{m}^{2}\epsilon }{[m[2(1-\pi )+\pi p]+m\pi (2-p){]}^{2}}=\epsilon$$

Peaks are matched by including a VAF tolerance $\sigma$ (e.g., 2%, in units of purity), which helps ameliorate the fact that we do not explicitly model noise affecting peak detection. The tolerance is used so that a comparison of whether a point is inside an interval, becomes a problem of measuring the overlap among the intervals. The intervals

1.6 $${{I}_{m}^{VAF}} =\left[{d}_{*}-\sigma , {d}_{*}+\sigma \right] {\text{and}} {I}_{m}=[{v}_{m}-\Delta {v}_{m},{v}_{m}+\Delta {v}_{m}]$$

are created with centre at ${d}_{*}$ with size 2 $\sigma$, and tested for overlap with the interval. The clonal peak for multiplicity ${m}$ is matched by ${d}_{*}$ only if the intervals overlap, i.e., $|{{I}_{m}^{VAF}}\cap {I}_{m}| >0$.

The QC status per copy state with two possible multiplicity values is defined by taking the status of the peak associated with the largest number of mutations ${n}_{m}$, i.e., as majority voting weighted by mutation counts. The value of ${n}_{m}$ is determined by binning the VAF distribution with 100 bins from 0 to 1 (size 0.01), and counting the number of mutations per bin of the matched peak. In this way, CNAqc passes a copy state if the tallest of its peaks is a pass, and is associated with more mutations than any failed peak. The sample-level QC status is based on an error metric that uses the actual distance between the centers of the intervals, ${d}_{*}$ and ${v}_{m}$, which is given by ${d}_{*}-{v}_{m}$ and described below.

An error metric is assembled across simple clonal CNAs to determine a sample-level score. Consider ${{w}_{k}}$ the normalized number of mutations mapped to copy state $k$, rescaled by 2 if the CNA is supposed to have two peaks. For every copy state and multiplicity, we have a pass or fail status from peak detection. We split pass (${P}_{k}$) from fail (${F}_{k}$) peaks, and define two scores by linear combination

1.7 $${\lambda }_{k}^{PASS}={\sum }_{{d}_{*}^{m} \in {P}_{k}}{w}_{k}\left({d}_{*}^{m}-{v}_{m}^{k}\right), {\lambda }_{k}^{FAIL}={\sum }_{{d}_{*}^{m} \in {F}_{k}}{w}_{k}({d}_{*}^{m}-{v}_{m}^{k})$$

where the subscript denotes the copy state (i.e., 1:0), and ${{d}_{*}}^{k}$ denotes the peak matched for multiplicity $m$ in state $k$. We define the CNAqc overall sample score $\lambda$

1.8 $$\lambda ={\sum }_{k \in {P}_{k}}{\lambda }_{k}^{PASS}+{\sum }_{k \in {F}_{k}}{\lambda }_{k}^{FAIL}$$

as a linear combination of terms that can be either positive or negative, depending on whether the matched peaks are on the right or left of the expected peaks. The sample score ${\lambda }$ is a weighted mean since by construction all the ${w}_{k}$ sum to one, and the terms constitute a partition. The overall sample status is finally taken by comparing ${{\lambda }_{k}^{PASS}}$ and ${{\lambda }_{k}^{FAIL}}$ and selecting the status corresponding to the largest.

### Complex clonal CNAs

Complex clonal CNAs are also QCed by a peak detection algorithm, but with a procedure that is simpler than the one proposed for simple clonal CNAs. So, while the sample-level QC is determined by simple CNAs, this procedure helps understanding if more complex states of aneuploidy inferred by a copy number caller are supported by data.

The procedure implemented in CNAqc works by matching VAF peaks, using a subset of the algorithms discussed above. Expected peaks are considered by applying Eq. (1) with mutation multiplicity ranging from 1 to the maximum between the major and minor allele counts of the considered segment (e.g., for a 4:2 copy state multiplicities from 1 to 4 are tested). Somatic mutations, as in the previous analysis, are pooled among segments with the same CNA, either across the whole genome or at the chromosome level.

To make this analysis faster, peaks in complex CNAs are inferred solely by the KDE heuristic, matching with the “closest” modality adopted for simple CNAs. Moreover, for every expected peak, the input parameter (purity tolerance) is used without performing any conversion from purity to VAF space. For complex CNAs, a table is compiled with every expected data peak, which depends on the mutation multiplicity, and a pass or fail status. No segments-level or sample-level scores are assembled in this case.

### Subclonal CNAs

CNAqc can QC subclonal CNAs for 2 subclones with relative proportions ${\rho }_{1}$ and ${\rho }_{2}$ satisfying ${\rho }_{1}+{\rho }_{2}=1$, and with simple CNAs. Compared to clonal segments, the QC of subclonal CNAs requires one to elicit the evolutionary trajectory of the subclones, because VAF peaks depend on the particular phylogenetic relationship between the subclones. In particular, VAF peaks depend on whether subclones originate upon branching from an unobserved ancestral state, or whether they evolve linearly. If they branch, they are siblings; otherwise, only one descends directly from the ancestral state. For instance, if the subclones are 1:0 and 2:0 with ancestral 1:1 it could either be that an heterozygous diploid cell, upon division, originated two distinct types of LOH (branching model 1:1 $\to$ 1:0 | 2:0), or could be that the diploid ancestor first loses one allele (generating 1:0), and then amplifies the remaining allele (linear model 1:1 $\to$ 1:0 $\to$ 2:0). One might also think that longer paths (e.g., 1:1 $\to$ 2:1 $\to$ 2:0 $\to$ 1:0) are less likely and assume that the shortest one is followed in most situations.

Fixing the time in which one subclone separates from the other is crucial for determining the multiplicity of the mutations accumulated before the split, which are shared across the subclones. Suppose, for instance, that subclones originated independently from a 1:1 cell: only mutations present in the original clone (ancestral mutations) will be shared, and subsequent CNAs will alter their multiplicity independently in each subclone. Conversely, if subclones evolved together up to a certain configuration before one of the two acquired a further alteration and expanded independently, the multiplicity of shared mutations will depend on that of the last shared copy state.

In order to account for the multiplicity of both shared and private mutations, CNAqc implements a recursive tree-generation algorithm that first (i) reconstructs the evolutionary tree (linear versus branching) that led a starting cell to develop into two subclones, and then (ii) computes multiplicities based on the tree. The starting state is a 1:1 diploid cell (default), but can be changed. The algorithm simulates progression from the initial to the final states by performing single-allele duplication, deletion, and mutation accumulation. Branching and linear scenarios are considered separately, and for every progression from X to Y only the shortest path is retained. Given paths, shared and private mutation multiplicities are determined based on the ordering of the amplifications and deletions, and the expected peaks are determined.

Consider two subclones ${n}_{A,1}{:n}_{B,1}$ and ${n}_{A,2}{:n}_{B,2}$, the expected peak for a shared mutation with multiplicity ${m}_{1}/{m}_{2}$ is

1.8 $${v}_{\{{m}_{1},{m}_{2}\}}= \frac{({m}_{1}{\rho }_{1} +{m}_{2}{\rho }_{2})\pi }{2(1-\pi ) + \pi ({\rho }_{1}{(n}_{A,1}{+ n}_{B,1}) +{\rho }_{2} ({n}_{A,2}{+n}_{B,2}))}$$

The total multiplicity of mutations shared is the sum of the total multiplicities. Private mutations found solely on subclone $i\in$ $\{\mathrm{1,2}\}$ have multiplicity $m\in$ $\{\mathrm{1,2}\}$, and the expected peaks are

1.9 $${v}_{{{m}_{i}}}= \frac{{{m}_{i}}{\rho }_{i} \pi }{2(1-\pi ) + \pi ({\rho }_{1}{(n}_{A,1}{+ n}_{B,1}) +{\rho }_{2} ({n}_{A,2}{+n}_{B,2}))}$$

Additional file 1: Fig. S3 shows the application of Eqs. (1.8) and (1.9) to an example of a bulk sample containing two subclones (Additional file 1: Fig. S3a), and the result of linear and branching models (Additional file 1: Fig. S3b) are compared. The two subclones have proportions ${\rho }_{1}=0.75$ and${\rho }_{2}=0.25$, and the tumor purity is$\pi =0.8$. In the example of Additional file 1: Fig. S3c, the subclones have CNAs ${n}_{A,1}{:n}_{B,1} = 1:1$ and${n}_{A,2}{:n}_{B,2 }= 1:0$, and have three expected peaks:

- ${{v}_{\{{m}_{1}=1,{m}_{2}=1\}}}$, for shared mutations;
- ${v}_{{m}_{1}=1}$, for private mutations on the first subclone and;
- ${v}_{{m}_{2}=1}$, for private mutations on the second subclone.

Since the multiplicities are either 1 or 0, expected peaks for the linear and the branching models are equivalent, though they correspond to different mutation groups. For example, a shared peak at 37% VAF comes from

$${v}_{\{{m}_{1}=1,{m}_{2}=1\}}= \frac{({m}_{1}{\rho }_{1} +{m}_{2}{\rho }_{2})\pi }{2(1-\pi ) + \pi ({\rho }_{1}{(n}_{A,1}{+ n}_{B,1}) +{\rho }_{2} ({n}_{A,2}{+n}_{B,2}))} = \frac{(0.75 +0.25)0.8}{2(1-0.8) + 0.8((2)0.75 +0.25))}=0.37,$$

and two private peaks instead come at ${v}_{{m}_{1}=1}=0.28$ and ${v}_{{m}_{2}=1}=0.09$.

More interesting is the case of two 2:1 and 2:0 subclones (Additional file 1: Fig. S3d), where the tumor purity is again $\pi =0.8$ and the subclone proportions ${\rho }_{1}=0.75$ and ${\rho }_{2}=0.25$, but here the branching and linear models have different peaks. The branching model (AB $\to$ AAB | BB) has the amplification and the deletion of the same allele (A) for the two subclones, and the amplification of the other (B) in one of the subclones. In this way, the major allele of one subclone (B) is the minor allele in the other. Five peaks are expected, of which only ${v}_{\{{m}_{1}=1,{m}_{2}=2\}}=0.38$ is shared and corresponding to mutations present on the ancestral allele, while ${v}_{{m}_{1}=2}=0.46$, ${v}_{{m}_{1}=1}=0.23$, and ${v}_{{m}_{2}=2}=0.15$ and ${v}_{{m}_{2}=1}=0.08$, correspond to private peaks for the subclones.

Instead, the second branching model (AB $\to$ ABB | BB) sees the independent amplification of the same allele (B) in the two subclones. Private peaks remain as in the above model, but shared peaks have a different position with ${v}_{\{{m}_{1}=2,{m}_{2}=2\}}=0.61$. Finally, in the only possible linear model (AB $\to$ AAB $\to$ AA) the 2:0 subclone arises from the 2:1 upon loss of the minor allele. This scenario is quite different from the previous two, as the number of expected peaks becomes four, two of which (${v}_{\{{m}_{1}=2,{m}_{2}=2\}}=0.61$, ${v}_{\{{m}_{1}=1,{m}_{2}=1\}}=0.31$) are shared and two (${v}_{{m}_{1}=1}=0.23$, ${v}_{{m}_{2}=1}=0.08$) are private.

### CCF estimation

In the field, many algorithms are available to compute CCFs [21, 27] and, some of the most effective ones (e.g., PyClone, Ccube), carry out simultaneous subclonal deconvolution along with CCF computation [34, 58]. The actual CCF value of a mutation should be obtained from denoised VAFs, i.e., VAFs removed of the binomial observational noise, which is the principle of tools like PyClone [13, 58]

However, Roth et al. and Gillis and Roth [13, 58] reported per-mutation CCFs, which are CCF estimates that retain the observational noise originating from VAFs. These values are more complicated to be interpreted because they are more prone to misinterpretation of the actual denoised CCF, as we discussed in section “Simulations, validation, and comparison to deconvolution tools”. For this reason, we developed in CNAqc a per-mutation CCF computation strategy that is able to QC these types of estimates. This functionality should be used as a source of orthogonal validation for deconvolution-based CCF computation algorithms that report this type of metric.

#### From VAFs to per-mutation CCFs

The equation to compute CCFs in CNAq converts the VAF (observed) $v>0$ of a mutation with multiplicity $m$ (to be estimated) into the CCF $c$ as follows

2 $${c}_{m}(\pi ,v)=v[{(n}_{A}+{n}_{B}-2)\pi +2]/m\pi$$

Per-mutation CCFs derive from VAFs and for this reason harbor the same observational noise. For example, an heterozygous clonal diploid mutation in a pure tumor has 50% theoretical VAF and CCF $c=1$, because 100% of cells harbor the mutation. If it sits on a 2:1 segment, its theoretical VAFs are either 33%—$m=1$ out of 3 copies—or 66%—$m=2$ out of 3 copies—but still $c=1$. However, since VAFs are observed with binomial noise, their observed values spread around the theoretical ones (e.g., around 33%), then CCFs are subject to the same noise rescaled by Eq. (2). This leads to a contradiction with the term “fraction” which by definition cannot exceed 1 but, for per-mutations CCFs converted by Eq. (2), VAF-associated noise spreads CCF values around their theoretical estimate. In this sense, the CCF of a clonal mutation spreads around $c=1$ using Eq. (2), as already reported in other popular papers—see, e.g., Figs. 12 and 13 in Methods of [16]. Nonetheless, subclonal deconvolution methods, e.g., [13] can filter out binomial noise and return values that range in [0,1]. However, this computation is carried out after CNAqc, as it goes beyond the idea of performing QC.

#### CCF computation algorithms in CNAqc

Tumor subclonal deconvolution algorithms, e.g., PyClone [13], denoise CCFs by computing cluster-level rather than per-mutation CCFs (i.e., per-clone/per-cluster CCFs). Instead, CNAqc determines per-mutation CCFs for mutations mapped to simple clonal CNAs, and a pass or fail status for the CCFs, determined by a metric to filter out mutations with uncertain estimates. In this way, per-mutation CCFs by CNAqc can be used to QC tumor evolution inferences that leverage CCF clusters, for instance [30, 59]. A limitation of CNAqc compared to other methods is to consider a subset of CNAs; this is motivated by the difficulty in phasing, from VAFs, mutation multiplicities from any CNA segment (complex and subclonal). Notably, the computations by CNAqc are however much faster, compared to analogous deconvolution tools [13, 15].

CNAqc offers two approaches to compute per-mutation CCFs: (i) an entropy model that uses binomial mixtures peaked at VAFs from Eq. (1) that phases mutation multiplicity using the mixture latent variables (capturing uncertainty from the latents), (ii) a rough model that uses the mixture, but does not model uncertainty. The former model, if there is too much uncertainty on the multiplicity of a mutation, can leave it undetermined (as its CCF), and return NA; this is how uncertainty is reported in CNAqc. The rough method, instead, will always assign a multiplicity $m\in \{\mathrm{1,2}\}$. The final QC status of some segments (e.g., all 2:1) is determined from the proportion of mutations with available CCF, with the idea to pass QC only if the number of assignable CCFs exceeds a user-defined threshold (default 10%). Therefore, the latter computation method will always pass QC because it does not implement uncertainty.

We first detail the rough approach; we describe the case of 2:0, 2:1, and 2:2, the others being trivial. To initialise a mixture:

1. We build two binomial densities from the theoretical expectations of the VAF peaks, i.e., ${v}_{1}$ and ${v}_{2}$, depending on the copy state, as defined in Eq. (1). This will create, for instance, one binomial with parameter $p=0.33$ and one with $p=0.66$ for a pure ($\pi =1$) tumor and 2:1;
2. We fix the number of binomial trials to the median coverage of the mutations, and compute the 1 and 99% quantiles of the data distributions to obtain a VAF range around each peak.
3. Finally, we count mutations that, according to VAF, map to either one or the other computed range. The number of mutations ${n}_{1}$ and${n}_{2}$, associated to multiplicity $m=1$ and$m=2$, is then used to obtain the mixing proportions ${\pi }_{1}={n}_{1}/({n}_{1}+{n}_{2})$ and ${\pi }_{2}=1-{\pi }_{1}$ to complete the model.

With these parameters, denoting by $Bin(x|{v}_{m})$ the binomial likelihood for mutation $x$ with multiplicity $m$, we can compute the mixture likelihood

2.1 $$f(\mathsf{X}|{v}_{m})={\prod }_{x\in \mathsf{X}}{\sum }_{m}{\pi }_{m}Bin(x|{v}_{m})$$

In a mixture model, we have latent variables $z$ as a matrix of mutations by clusters, for which we define, the probability of assigning read counts data for mutation $n$ to component $i\in \{\mathrm{1,2}\}$

2.2 $${\mathsf{z}}_{n,i}=f(X|{v}_{i})/[f(X|{v}_{1})+f(X|{v}_{2})]$$

With these latents, every row of matrix $z$ is a categorical random variable reporting the probability of assigning $m=1$ or $m=2$ to a mutation, for which we define the entropy

2.3 $$H({\mathsf{z}}_{n})={-\mathsf{z}}_{n,1}log({\mathsf{z}}_{n,1})-{\mathsf{z}}_{n,2}log({\mathsf{z}}_{n,2})$$

The entropy is maximal if ${\mathsf{z}}_{n,1}{=\mathsf{z}}_{n,2}$, i.e., the mutation is equally likely in single and double copy, and is therefore uncertain to be assigned. As opposite, the entropy is minimal if ${\mathsf{z}}_{n,1}=1$ and ${\mathsf{z}}_{n,2}=0$, or vice versa. If the entropy is low, the mutation is then difficult to phase to single- or double-copy mutations, using VAFs. The shape of the entropy resembles—by construction—a growing curve with a central spike, which we use to create a simple criterion to discriminate high from low entropy. The geometric intuition of this criterion is that at the crossing of binomial densities peaked at ${m}_{1}$ and at ${m}_{2}$, if the $H({\mathsf{z}}_{n})$ is high we cannot confidently phase mutation multiplicities. The amount of binomial overlap depends on coverage and purity, which is the technical reason CCFs are more “uncertain” for low-resolution data.

CNAqc uses a simple peak detection heuristic (similar to the one for QC) to inspect $H({\mathsf{z}}_{n})$ and determine peaks $\{{h}_{1},{h}_{2}\}$ around the spike. Every mutation in the range

2.4 $${I}_{NA}=[{h}_{1},{h}_{2}]$$

cannot be confidently assigned multiplicity values and are therefore undetermined using the entropy method. Their per-mutation CCFs is also reported as an NA value (Not assigned).

The rough approach works as opposite, as it determines the midpoint $o={v}_{1}+ ({v}_{2}-{v}_{1}){\pi }_{1}$ between the two expected theoretical VAF peaks ${v}_{1}$ and ${v}_{2}$, given the mixing proportion ${\pi }_{1}$ of the first mixture component. The midpoint is computed by weighting each of the two peaks proportionally to the number of mutations that appear underneath each peak, which we compute like with the entropy method. The midpoint is a cut: $x<o$ are phased to a single copy, values above to two copies. This procedure requires data with good general quality because it assumes that all mutations can be phased correctly by a hard VAF split, a fact that depends largely on coverage and purity.

When multiplicities have been determined, per-mutation CCFs are computed by using Eq. (2).

### Other features

#### Genome fragmentation detection

Some recently identified patterns of somatic CNAs can be attributed to the presence of highly fragmented tumor genomes, termed chromothripsis and chromoplexy, or localized hypermutation patterns, termed kataegis [60]. While these can be identified using dedicated tools, CNAqc offers a simple statistical test to detect the presence of potential over-fragmentation in a region of interest, a prerequisite that could point to the presence of such patterns. CNAqc analysis does not substitute dedicated tools, but provides preliminary information to determine what parts of the genome might be run with ad hoc methods.

At the level of chromosome arms (1p, 1q, 2p, 2q, etc., or subsets), CNAqc uses the length CNA segments to classify “long” and “short” fragments with a cut parameter $\mu >0$ (default 0.2), and a segment longer than a fraction $\mu$ (rescaled to 100) of the arm is considered long. Recent evidence from large pan-cancer studies can be used to calibrate this parameter to cancer-specific values [5].

Then, a null hypothesis is used to compute a *p*-value using a binomial test based on $k$, the number of trials given by the total segments in the arm, and the observed number of short segments $s$. The binomial distribution for ${H}_{0}$ is defined by $\mu$, and the null is the probability of observing at least $s$ short segments. CNAqc defines a one-tailed test for whether the observations are biased towards short segments, adjusting the *p*-value for family-wise error rate by Bonferroni, i.e., dividing the desired $\alpha$-value by the number of tests. This test is applied to a subset of chromosome arms with a minimum number of segments and that “jump” in ploidy by a minimum amount (empirical default values estimated from trial data). The arm-level jump is determined as the sum of the difference between the ploidy of two consecutive DNA segments. These covariates are similar to those used to infer CNA signatures from single-cell low-pass WGS [11].

#### Segment smoothing

Smoothing is an operation that can be carried out, at the level of clonal segments, before testing for over-fragmentation. This operation does not affect the ploidy profile of the calls, but reduces the amount of breakpoints that otherwise inflate the *p*-value of the binomial over-fragmentation test in CNAqc.

We implemented this operation to reduce the number of segments reported by a caller, because we observed that in real data several callers break contiguous segments even without actual copy number changes (i.e., the same numbers for the major and minor alleles are reported, but a breakpoint is present to break a segment). These types of behaviors are arguably linked to the segmentation algorithm of the caller, and its ability to call segments over a certain genome length. Therefore, in CNAqc, by smoothing we merge two contiguous clonal segments if they have exactly the same allele-specific profile. The smoothing procedure is controlled by a distance parameter with 1 megabase as default value, which avoids merging segments that are above that distance apart.

#### Chromosome-level analyses

CNAqc can perform QC-based analysis at the chromosome level, namely for each chromosome separately. This functionality can be useful to spot samples where the estimate of the bulk purity is correct, but there are large segments with miscalled allele-specific segments (e.g., large sections of the genome that are called triploid while they should be diploid).

We explain the advantage of this functionality by modifying the calls for the PCAWG hepatocellular carcinoma ca5ded1c-c622-11e3-bf01-24c6515278c0. First, we retrieved SNVs mapped to diploid (1:1) and triploid (2:1) segments; genome-wide allele-specific consensus CNAs are characterized by ploidy 2 and a purity of ~ 85%. We then simulated the unlikely case in which a copy number caller fails to call the diploid regions as such, and instead assigns them a triploid 2:1 state (Additional file 1: Fig. 2a-c).

Genome-level peak analysis computes a quality score for pooled 2:1 segments, failing the whole-triploid solution at the correct purity, and proposing a purity correction of ~ 7% (Additional file 1: Fig. 2c). For this weird case, however, the chromosome-level CNAqc analysis can identify the source of error (Additional file 1: Fig. 2d). In this case, we easily verify that CNAqc fails the triploid solution for each chromosome that contains mostly non-triploid segments—chromosomes 2, 3, 4, 6, 7, 10, 11, 13, 14,15, 16, 18, 19, 20, and 21—while CNAqc passes the ones containing a significantly large triploid region—chromosomes 1, 5, 8, and 17. While these types of errors in the data are unlikely because a copy number should detect different input depth ratios and B-allele frequencies, this type of analysis can be helpful to inspect, at narrower resolution, the quality of the input segments.

### Simulations, validation, and comparison to deconvolution tools

#### Peak detection (base simulations)

We tested CNAqc on a synthetic dataset of ~ 20,000 tumors, generated to mimic data that we observed in real patient tumors. We first simulated synthetic VAFs from clonal CNAs generated with breakpoint distributions following Poissons (6 segments per chromosome, on average, and a Dirichlet copy state concentration 1 for 1:0, 1 for 2:0, 6 for 1:1, 2 for 2:1, and 1 for 2:2). Then we simulated Poisson-distributed coverage with median depth 30 × , 60 × , 90 × , and 120 × , and set purity to 0.4, 0.6, 0.8, or 0.95. The idea of this test was to simulate a tumor with purity ${\pi }$ and run CNAqc with an input purity that contained a positive or negative error $\varphi$, i.e., we imputed CNAqc purity ${\pi }+ \varphi$. Then, for different values of the input tolerance $\epsilon$, i.e., the maximum purity error we want to tolerate in CNAqc, we run the tool with default peak-matching parameters and perform quality control. Ideally, when the input error $\varphi$ is lower than tolerance $\epsilon$, CNAqc should pass the sample.

We performed QC applying an error on the purity in range [0; 0.2] with intervals of length 0.02, setting a tolerance on the purity error ranging in [0.01; 0.05] with intervals of length 0.004. We tested CNAqc on 100 simulated tumors for any combination of all the parameters and consistently observed that, as the purity error $\varphi$ exceeds tolerance $\epsilon$, the proportion of failures approaches 100% (Additional file 1: Fig. S6). For instance, setting a tolerance parameter of 2%, we can accept a purity error of 5% at most. Over this threshold, the proportion of failed samples reached maximum at ~ 7%. One can check this behavior for the samples of purity 0.95 and coverage 90 × : for a tolerance of ~ 2%, the proportion of rejected samples is close to 0% when the purity error is smaller than 5%, it increases to 70–75% for a purity error of ~ 5/6%, while for a purity error of ~ 10% the fail proportion is 100%. From the test, we also observed that the ability of CNAqc to detect samples with incorrect purity improves consistently as we increase coverage, with this effect more evident for samples with high purity. For the same tumors, we also computed CCFs and the proportion of mutations for which CNAqc could not phase multiplicity (for 2:0, 2:1, 2:2). We see the percentage of unassignable mutations (Additional file 1: Fig. S7) to decrease as we increase coverage and purity, meaning that the computation of CCFs and multiplicities depends on these parameters. The observed trend was expected, since at low coverage and purity we have the overlaps between clonal clusters which makes it harder to phase multiplicity from VAFs.

#### Validation with single-cell copy number data

We validated the methodologies implemented in CNAqc by adopting complementary single-cell copy number data. We collected low-pass single-cell data using the Direct Library Preparation (DLP +) protocol from an ovarian cancer cell line [31]. DLP + is an amplification-free library preparation protocol to generate high-resolution single-cell WGS data suitable for cell-level calling of both CNAs and SNVs. We used this type of data to assemble monoclonal and polyclonal pseudo-bulk populations and validate all the functionalities of CNAqc.

We first clustered cells with similar allele-specific CNAs which we computed using SIGNALS [33]. These clusters correspond to monoclonal populations composed of 100% tumor cells and are characterized by specific CNAs (Additional file 1: Fig. S9). Then we obtained SNVs per cell, generated also in [31]. From cluster assignments and read counts per cell, we generated a pileup of read counts per clone (sum of both reference and alternative allele counts for all cells in a cluster/clone) mimicking a WGS assay for each tumor clones. We then selected clusters G (111 cells), H (77 cells), and I (177 cells) because among the larger clusters they are the ones with the least noisy VAFs and the most common monosomy and tetrasomy segments. We used CNAqc to QC the expected purity of 100% (purity by assembly) per clone (Additional file 1: Fig. S10). As expected, our model assigned a pass score to all datasets, for both simple and complex CNAs (3:0 and 4:0).

Then, we tested how accurate the predictions of CNAqc are in terms of purity correction estimates, if one uses a wrong input purity. From the cases above (true purity 100%), we imputed a purity in the form $1-\varphi$, where $\varphi$ models the error, and measured the ${R}^{2}$ correlation between $\varphi$ and $\lambda \in \mathfrak{R}$, the purity adjustment returned by CNAqc (Additional file 1: Fig. S11). Peak analysis was run multiple times decreasing input purity from 100 to 90% (1% step, 15 repetitions per point). The tested samples from Additional file 1: Fig. S9 using (a) a pileup of clonal CNAs common to clusters G, H, and I (Additional file 1: Fig. S12), in 1:0 and 2:0 regions, (b) cluster G restricted to 1:0 segments, (c) cluster H restricted to 1:0 segments, and (d) cluster I restricted to 1:0 and 2:0 segments. In every case, we measured that the proposed correction is in perfect agreement with the input mismatch (correlation coefficient 0.88 < ${R}^{2}$  < 0.99, *p*-value *p* < 2.2e − 16), therefore showing that purity correction estimates in CNAqc are precise.

Finally, we measured accuracy to QC subclonal CNAs (Additional file 1: Fig. S10) by merging some clusters from previous tests into larger clones with more mutations; this was necessary since single clusters were quite small and had few SNVs mapping on subclonal CNAs. We pooled cells from clusters G, H, and I in Additional file 1: Fig. S9 and retained clonal CNAs common to all clusters, plus subclonal segments where clusters H and I have the same CNA and differ from cluster G. Then, we mixed all the cells (111 for G, 77 for H and 177 for I), obtaining a mixture with ~ 70% cells from merged cluster H + I, and ~ 30% from cluster G. CNAqc could easily validate clonal CNAs as in the previous tests. In this sample we performed QC of 2 subclonal CNAs on chromosomes 4 and 11, harboring 323 and 271 SNVs each (Additional file 1: Fig. S10). CNAqc detected the expected peaks, therefore supporting the presence of subclonal CNAs: we could validate a mixture of 1:1/2:2 populations, and a mixture of 1:1/2:1 populations. In both cases, peaks from the linear and branching evolution models were observed, making it hard to decide precisely what evolutionary model explains best the origin of these populations. In order to stress test the evolutionary modelling underneath our QC procedure for subclonal CNAs, we also took further data from [32] and generated allele-specific CNAs for these cells using SIGNALS [33]. This time we also phased alleles (Additional file 1: Fig. S11), which allowed us to identify a sample with clear subclonal CNAs and allelic imbalance, consistent with the original publication. In particular, we found a set of cells with monoclonal 2:1 chromosome 1, and polyclonal chromosomes 2–4 (2 clones). On chromosomes 3 and 4, the populations were found to be triploid and tetraploid, while on chromosome 2 they were found to be triploid with mirrored allelic imbalance, as reported in the original publication. In practice, on chromosome 2, one population (58% of cells) had genotype AAB and the other (42% of cells) was ABB, where A and B are the alleles of the unobserved ancestral diploid population (or, in our notation, they were 2:1 and 1:2). CNAqc could validate the subclonal CNAs in all the chromosomes (Additional file 1: Fig. S12). The case of chromosome 2 was particularly interesting, because the tool compared the evolutionary models.

1. A1B1 $\to$ A1A2B1 | A1A2B1 (branching with imbalance)
2. A1B1 $\to$ A1A2B1 | A1B1B2 (branching with mirrored imbalance)
3. A1B1 $\to$ A1A2B1 $\to$ A1A2B1 (linear with imbalance)

Models 1 and 3 predict the same peaks, with shared mutations peaking at 0.667 and 0.333, and private mutations peaking at 0.192 and 0.141. Model 2 instead predicted shared mutations (in 3 alleles out 6 due to imbalance) with peaks (2*0.58 + 1*0.42)/(0.58*3 + 0.42*3) = 0.57 and (1*0.58 + 2*0.42)/(0.58*3 + 0.42*3) = 0.47. The data distribution showed a clear peak at VAF around 50%, and CNAqc was therefore able to validate the subclonal segment and identify the correct branching evolution model A1B1 $\to$ A1A2B1 | A1B1B2 with mirrored allelic imbalance.

Finally, we sought to use single-cell data also to validate mutation multiplicity phasing in CNAqc, while accounting for uncertainty in the estimate. One limitation of the data at hand was that the multiplicity of input mutations at the single-cell level is unknown. Therefore, we opted to validate CNAqc computations by checking if mutations flagged as uncertain from the pseudo-bulk are also difficult to phase at the single-cell level, which seemed a reasonable test for these data. Using single-cell data for cluster A (Additional file 1: Fig. S9), we computed per-cell VAFs for mutations in 2:1 regions with good mappability and quality scores. Second, we computed per-cell multiplicity per mutation: as with bulk, we assigned one copy ($m=1$) if the single-cell VAF was closer to 0.33 than to 0.66, and two copies ($m=2$) for the opposite case (closer to 0.66 than to 0.33). We used a majority score from all cells to vote for multiplicity and resist noisy VAFs from single cells (caused by low coverage per cell < 5 reads, not shown). We also registered the proportion of cells that vote for single or double copy. Then, we computed VAFs from the pseudo-bulk of these segments (Additional file 1: Fig. S15a) and identified mutations which CNAqc phased as uncertain in terms of multiplicity. We compared CNAqc assignments from bulk to majority voting with single cells, after classifying consensus in three ranges: > 85% (high), > 50% (intermediate), and < 50% (low). Results (Additional file 1: Fig. S15b) show that CNAqc assignments of multiplicity from pseudo-bulk match assignments from single cells for 83% of mutations, and for 98% of mutations with consensus > 85% among single cells, while CNAq considers uncertain the phasing of a set containing 13% of the mutations (40% of mutations with low single-cell consensus). Notably, only for 3% of the mutations CNAqc phasing fails to match consensus-based phasing. Finally, we computed the histograms of voting values split by CNAqc mutation assignment (one copy, two copies, and uncertain, Additional file 1: Fig. S15c), and we observed mean voting support 70% for mutations assigned one copy, 61% for two copies and 43% for uncertain mutations. Overall, these analyses show that the mutations flagged as uncertain by CNAqc are largely the same for which single-cell multiplicities are difficult to estimate.

####  Automatic -calibration via false positive rate curves

The result of sample-level QC (pass or fail) depends on the maximum purity error $\epsilon >0$ specified by the user. CNAqc offers a function to automatically determine what purity error parameter should be used, for a particular combination of coverage and tumor purity, in order to minimize the false positive rate (FPR) of the tool, as determined by simulations.

To calibrate this functionality (Additional file 1: Fig. S8a), we sampled 100 distinct tumor genome segmentations, spanning sample purity $0.15\le \pi \le 0.9$ and median coverage $20\le cov\le 120$. For each tumor and each purity/coverage value, we tested the input purity error $0.01\le \epsilon \le 0.1$ (1–10%) discretized by 1%, and for each of these values we sampled 10 datasets mimicking a CNAqc input run with tumor purity of the form $\pi + \epsilon +\varphi$ where $\varphi \sim U[0, 0.03]$. So, we have imputed to the tool a purity that is close to the true one ($\pi$) modulo the tolerance ($\pi +\epsilon$), but positioning the tool in the scenario in which the actual input should be failed because $\varphi >0$. Note that since the error margin is 3%, this borderline scenario represents a configuration in which CNAqc should fail a sample, but the task is difficult because the input is “close” to the cut point where the sample could be passed (theoretically). In this way, we could compute the FPR for each value of purity/coverage as a function of $\epsilon$, which were used to fit a generalized linear model (GLM) for every purity/coverage value (Additional file 1: Fig. S8a). Results from these tests give the expected results; in particular, we clearly observe gradients for both purity and coverage, and the slope of the regression correlates with data quality (higher resolution data allows lower FPR for broad values of $\epsilon$). For instance, with coverage 20 and purity 0.15, the lowest FPR is still 30% at $\epsilon =0.01$—because data quality impacts peak matching—and peaks at 60% at $\epsilon =0.1$. For better quality (e.g., coverage 120, purity 0.9), instead, FPR is well below 10% even with very stringent $\epsilon =0.01$, and remains substantially low for larger $\epsilon$.

The algorithm to determine which $\epsilon$ minimizes FPR for a particular combination of coverage and tumor purity, works as follows. First, one selects the maximum FPR accepted $\mu >0$ (default 10%); the GLM fits of the training set are used to invert the FPR and determines the largest $\epsilon$ with desired FPR below $\mu$ (Additional file 1: Fig. S8b,d). If the regressed $\epsilon$ exceeds some input range of values (determined by the user), the regressed value is capped. All the regressed values are then interpolated with a 2D Akima non-smoothing spline that gives good fits to curves with a second derivative that changes rapidly [61]; the required point estimate for $\epsilon$ is determined from the interpolation of the spline values (Additional file 1: Fig. S8c,e). The interpolation is carried out only in the range of values of the training set, and constraints on $\epsilon$ required by the user are finally enforced (e.g., so that one can determine the $\epsilon$ which associates with FPR $\mu <0.7$ while requiring no purity errors below 5%).

#### Comparison to deconvolution methods

Some of the functioning of CNAqc is inspired by the design of subclonal deconvolution methods [13, 15, 17, 27, 30, 51, 59, 62]. Therefore, we sought to compare CCFs by CNAqc with the one obtained by Ccube (default parameters), a CCF computation method developed by the PCAWG Evolution and Heterogeneity Working Group [34].

In Additional file 1: Fig. S16 (panel a), we show the correlation among the CCF values computed by Ccube and CNAqc (entropy method) in PCAWG. In the plot, we annotate the proportion of cases, split by copy state and mutation multiplicity, where the estimates are different after rounding to the second digit. We observe that the tools report the same CCF for ~ 99% of the analyzed mutations, whenever CNAqc identifies a reliable CCF value. We remark that a feature of CNAqc is reporting the percentage of mutations where the CCF cannot be unequivocally determined. In the above statistics, the CCF values are therefore computed only for mutations where the uncertainty is not present in CNAqc. The information regarding uncertainty is however very helpful to integrate CNAqc with other tools for CCF computations, as we show with two examples from our test. In Additional file 1: Fig. S16 (panels b–g), we report an example PCAWG case where the CCFs are in perfect agreement (1 out of 307 mutations in 2:2 segments with different CCF). In Additional file 1: Fig. S17, instead, we show a case where CNAqc detects uncertainty in 14% of input triploid mutations, informing of potential challenges in using CCFs for those mutations. In that case, the uncertainty is explained by the intermixing between two clonal picks in triploid 2:1 segments. Ccube assigns multiplicity 2 to a group of clonal SNVs at the right tail of the lowest clonal pick. The consequent CCF distribution breaks the expected clonal peak around ~ 1, alluding to the presence of two close CCF clusters. This is due to Ccube assigning some single-copy mutations $m=2$ and vice versa. The entropy-based method by CNAqc highlights 14% of 2:1 mutations as uncertain, including the ones mistaken by Ccube. In turn, CNAqc assigns a FAIL status to these mutations with default values (cutoff > 10%). Notably, the CCF distribution returned by CNAqc, which uses 86% of total mutations once the 14% unassignable are removed, is correctly peaked at ~ 1.

Errors in CCFs can affect downstream subclonal deconvolution, which in turn inflates evolutionary statistics (e.g., number of subclonal clusters, clonal complexity). In this example, miscalled multiplicities generate a spurious cluster in the CCF distribution fit by Ccube, which leads to subclonal cluster 2 (panel g, Additional file 1: Fig. S17). Even after removal of 14% CCFs flagged as uncertain by CNAqc, Ccube still assigns the wrong mutation multiplicity to a significant number of variants and infers the spurious CCF cluster (panel h, Additional file 1: Fig. S17). For this reason, reporting a FAIL status in CNAqc informs that multiplicity computation in this sample is highly confounded by intermixing of VAFs, cautioning the interpretation of downstream deconvolution analyses.

#### Wall-time performance against deconvolution methods

In order to understand how performance scales with sample size, we compared the wall-clock time of CNAqc against common deconvolution tools. We chose SciClone [14], Ccube [34], and Pyclone-vi [58] to represent a diverse set of popular algorithms for deconvolution. To build the dataset, we subsetted all the mutations in diploid regions from a melanoma sample of the PCAWG cohort (patient id DO220877) leading to a total of 207,508 mutations. This is the PCAWG sample with highest mutational burden in the cohort. Then, we sampled $N=\{\mathrm{500,1000,25000,5000,1000,25000,50000}\}$ SNVs; this process was repeated 10 times to have 10 replicates for each N. The CNAqc analysis for peak detection was run with default parameters. Similarly, default parameters were also used for SciClone (default one-dimensional deconvolution) and Ccube (but with numOfRepeat = 1); Pyclone-vi was run with beta-binomial likelihood, number of clusters from 1 to 10 and 30 repetitions (Additional file 1: Fig. S5).

CNAqc was the fastest tool, capable of processing up to 500,000 mutations in ~ 60 s, while tools based on variational inference were about an order of magnitude slower. The latter two algorithms ranged from being 4 to 16 times slower than CNAqc for our range of tests (consider the log-scale in the plot *y*-axis), and the performance gap increased with larger N. Notably, SciClone took an average of 2 h to process 50,000 mutations, which is 128 times slower than CNAqc as suggested by a log difference of 5. In all tests, CNAqc, Ccube, and Pyclone-vi scaled approximately exponentially, while SciClone showed a jump from 25,000 to 50,000 mutations. All simulations were performed on a machine with 36 Intel(R) Xeon(R) Gold 6140 CPUs @ 2.30 GHz and 220 GB of RAM (Ubuntu 20.04 LTS, Python 3.8.2 and R 4.1.0).

### Analysis of patient data

#### WGS data at PCAWG

We performed QC of the entire Pan-Cancer Analysis of Whole Genomes (PCAWG) cohort [28]. First, we collected the sample list (file pcawg_sample_sheet.tsv) at [63] with 2955 identifiers from PCAWG (2834 unique donors), and removed samples for which consensus CNAs were unavailable, identifying 2778 (2658 unique donors) WGS samples from 20 primary sites in 48 distinct projects. Then, we filtered samples that did not carry at least 20 mutations on each of the called copy number configurations, considering simple clonal segments, to compile our final list of working samples. We note that among the samples we did analyze and that passed QC, there were 75 samples that, for distinct reasons, were originally graylisted by the consortium. We report all the samples that we included, excluded a priori (no consensus copy number calls available) and filtered (less than 20 mutations) in Supplementary Table S1.

Results for the analysis for simple and complex CNAs are shown in Fig. 5 and Additional file 1: Fig. S28. Tumor types with higher prevalence of complex CNAs are esophageal adenocarcinoma (ESAD), liver cancer (LIRI), melanoma (MELA), ovarian cancer (OV), pancreatic cancer (PACA), and breast cancer (BRCA); liver and pancreatic cancers account for the majority of the samples of the cohort. Among these CNAs, those with ploidy beyond 6 are rare and more frequent in those tumor types with a higher number of CNAs. One may argue that this pattern might be linked to a general tendency of those tumor types to acquire these kinds of anomalies. The mean matched peaks for segments with ploidy $\le 6$ is above 50% in most cases and tends to be lower for segments with higher ploidy (increasing again in the final part of the spectrum), a phenomenon that could be due to the lack of an evolution-based QC for complex CNAs in our tool.

Results for subclonal CNAs are shown in Additional file 1: Fig. S19 and S20. The top 5 tumor types carrying the most subclonal CNAs are esophageal adenocarcinoma (ESAD), liver cancer (LIRI), melanoma (MELA), pancreatic cancer (PACA), and breast cancer (BRCA). Four out of five of these tumor types also carry clonal complex copy numbers, supporting the hypothesis of some biological mechanism involved in CN instability in these types of tumors. To match peaks in subclonal CNAs, we computed all possible evolutionary models from a 1:1 starting state. We determined which model (linear or branching) best explains the data from the highest percentage of matched peaks; in case of a tie an “ambiguous” flag was assigned and, if no model could match at least 50% of peaks, “none” was assigned. By imposing this restriction, we were able to assign > 87% of segments to a model. The first thing we notice from Additional file 1: Fig. S21 is that the most frequent subclonal CNA is either the loss or the acquisition of a single allele, corresponding to 1:0–1:1 and 1:1–2:1. This is not surprising as these events are explainable with a deletion of a single allele in one cell duplication. Other CNA combinations must instead be explained by at least two independent losses or duplication events, making them less likely. The less frequent combination is 1:0–2:0, possibly due to the fact that a duplication and a deletion event occurring on the same allele seem unlikely, unless the tumor has a high instability and predisposition to achieve CNLOH. The same pattern repeats across tumor types, with tumors tending to have more simple subclonal events also showing a higher number of complex events, supporting the hypothesis that some tumor types are more prone to develop CNAs [5, 17].

In most cases, a linear modelling of the dynamics of the formation of the subclones (meaning that the second subclone arises from the first) seem to better explain the data with respect to a branching modelling in which both subclones independently originate from a diploid ancestor. There are two exceptions to this statement; first, for subclones with 1:0–1:1 karyotypes (i.e., a 1:0 subclone and a 1:1 subclone), where there is no difference in the peaks expected by the linear and branching models; therefore, the models are indistinguishable. This is evident from Additional file 1: Fig. S21b and S21c, where in all tumors the number of matched peaks for this combination of subclonal karyotypes is equivalent. The second exception is for 2:0–2:1 subclones, in which the branching model seems to explain the data better. The reason for this might be that, while both models require at least three steps for the two subclones to develop the karyotype 2:0–2:1 from a diploid 1:1 ancestor, the branching model can take into account both the path in which the major allele is the same in both clones, or is the major in one clone is the minor in the other. The linear model’s shortest path, followed by assumption in CNAqc, can only include the first of the two scenarios.

#### WGS data at Genomics England

We further validated CNAqc by performing control of 235 samples obtained from the Genomics England Consortium [19] using the Illumina DRAGEN™ pipeline [64]. We started from data released in [35], using 301 patients for which we had complete DRAGEN™ calls available and removed those belonging to tumor types with less than 10 samples associated. Since by default the tool can identify putatively heterogeneous regions but does not give an estimate of the number and prevalence of subclonal populations we had to derive those quantities using some heuristics. We developed a simple procedure to estimate subclone proportions by assuming the presence of two populations, following the same heuristics implemented in the popular tool BATTENBERG [15]. Without loss of generality, we will define the integer solution of DRAGEN™ in terms of minor and total copy number as subclone 1.

We started from the floating point estimates for:

- The minor allele (mCNF);
- The total copy number (CNF);
- Minor allele frequency (MAF);

and determined:

- Integer minor and total allele copy number for subclone 1 ($mC{N}_{1}$ and $C{N}_{1}$);
- Integer minor and total allele copy number for subclone 2 ($mC{N}_{2}$ and $C{N}_{2}$)

We proceed as follows:

1. Set a grid of values for $mC{N}_{2}$, default 0 to 10;
2. Estimate subclonal proportion of the segment using the formula
  $${\rho }_{1}=\frac{mCNF-mC{N}_{1}}{mC{N}_{2}-mC{N}_{1}}$$
3. Estimate $$C{N}_{2}$$ by
  $$C{N}_{2}=\frac{CNF-(1-{\rho }_{1})*C{N}_{1}}{{\rho }_{1}}$$
4. Calculate the MAF error, given ${\eta }_{1}=\frac{mC{N}_{1}}{{CN}_{1}}$ and ${\eta }_{2}=\frac{mC{N}_{2}}{{CN}_{2}}$
  $$MA{F}_{err}=|MAF-\pi [{\rho }_{1}*{\eta }_{1}+(1-{\rho }_{1})*{\eta }_{2}]-(1-\pi )*0.5|$$
5. Choose the $mC{N}_{2}$ with the lowest MAF error $MA{F}_{err}$
6. Filtered negative solutions where $C{N}_{2}<2mC{N}_{2}$ as well as solutions where $MA{F}_{err}<\epsilon$, with $\epsilon =0.1$ (default threshold)

The intuition of this procedure is as follows. Given the assumption of a two subclone population and a maximum $mC{N}_{2}$ of 10, we need just $mC{N}_{1}$ to determine ${\rho }_{1}$ using the formula at point 2, together with the $mCNF$. To provide an example, let us assume a segment with $mC{N}_{1}$ value of 1, a $mCNF$ of 1.8, a $C{N}_{1}= 3$, a $C{NF = 4.2}$ and, $MAF =0.4$ we will test $mC{N}_{2}=[\mathrm{2,3},\mathrm{4,5},6]$ and assume purity $\pi =1$ for simplicity (note that we skipped 1 as it implies a division by zero). For the 5 values of $mC{N}_{2}$ we get respectively ${\rho }_{1}$ values of [0.8,0.4,0.26, 0.2, 0.16], by applying the formula at step 3 we estimate $C{N}_{2}$, which in this case is [5, 7–9, 11]. We can clearly see how some solutions do not make sense (2 $mCN > CN$) and get filtered in step 6. The estimated MAF errors in this case are [0.05, 0.01, 0.05, 0.11, 0.12], so we will choose the one with the lowest error, namely $mC{N}_{2}$= 3 and 7, this setting corresponds to two subclones one at frequency 80% with karyotype 1:2 and one at 20% with karyotype 3:4. As the absolute error is lower than 0.1, we accept the solution.

Upon converting DRAGEN™ continuous estimates into clone-level CNAs, we set all the parameters as in the analysis of PCAWG to allow for a fair comparison among Genomics England and PCAWG cohorts.

#### WES data at TCGA

We collected WES data from $n=48$ lung adenocarcinoma samples available in TCGA LUAD [29], selecting the 24 ones with top and bottom consensus purity estimate (CPE) by TCGA. We report example cases in Additional file 1: Fig. S23, where QC values are obtained by using somatic SNVs, CPE purity, and default CNAqc parameters. The case in panel (a), sample TCGA-53–7624-01A, is 84% pure and the inferred ploidy is correct, but purity is slightly overestimated. The case in panel (b) is 82% pure, but with a similar pattern. The case in panel (c) is an example of a VAF distribution that is low resolution because the sample has 30% purity, and in this case, it is difficult to assess if the small peak matched by CNAqc is a noise artifact. The case in panel (d) is 83% pure, with good calls and the one in panel (e) is 32% pure and passed because most of the tetraploid mutations are correct, but it contains a poorly peaked VAF distribution in triploid states (2:1, 47% of the mutational burden). In this last case, CNAqc struggles to detect peaks from VAF; this is another example of low-resolution VAF distribution.

We used CNAqc to select among multiple purity estimates provided by different TCGA callers, focusing on the LUAD case (a) from Additional file 1: Fig. S23. In TCGA, we obtain purity estimates from CPE, which is the consensus among ABSOLUTE, ESTIMATE, IHC, and LUMP. For this sample, ESTIMATE, IHC, and LUMP provide similar purity and determine the value for CPE. However, we fail that estimate with CNAqc and instead pass only ABSOLUTE (69% purity, Additional file 1: Fig. S24). We extended this test to 1464 TCGA samples from 10 distinct tumor types (Additional file 1: Fig. S25 and S28) with suitable data for CNAqc (i.e., at least 200 somatic mutations). We obtained samples from the cohorts Bladder Urothelial Carcinoma (BLCA), Breast Invasive Carcinoma (BRCA), Colorectal Adenocarcinoma (COAD), Glioblastoma (GBM), Head-Neck Squamous Cell Carcinoma (HNSC), Kidney Renal Clear Cell Carcinoma (KIRC), Lung Squamous Cell Carcinoma (LUSC), Rectum Adenocarcinoma (READ), and Uterine Corpus Endometrial Carcinoma (UCEC).

First, we computed QC with CNAqc with maximum tolerated purity error 5% ($\epsilon =0.05$) for all possible purity values for the ABSOLUTE, ESTIMATE, IHC, and LUMP tools, as well as for consensus CPE purity. We report in panel (a) of Additional file 1: Fig. S25 cases split by QC status (maximum tolerated purity error 5%) as determined from the run with TCGA consensus purity. Strikingly, as in Additional file 1: Fig. S24, we immediately observe a number of cases in which, while CNAqc fails the CPE estimate, there are at least $\ge 1$ method different from CPE that proposes an acceptable purity. Notably, for 901 cases where the CPE purity is failed by CNAqc (60% of 1464), upon splitting the status by tool in panel (b), we note for instance that ABSOLUTE often provides a purity estimate that would pass the sample, similarly to the case shown in Additional file 1: Fig. S24. This is particularly true for samples from the HNSC, LUAD, COAD, LUSC, and BRCA cohorts. Indeed, it is worth noticing that ABSOLUTE gives the user the opportunity to include information coming from the VAF of SNVs to support the CNA inference. This feature is probably the reason why its estimates often are more accurate and consistent with the QC-based approach of CNAqc. Conversely, methods such as ESTIMATE very often provide failed purity estimates for the samples (especially from the HNSC, LUAD, LUSC, and BRCA cohorts). If we were to rank and select the best purity as determined by CNAqc instead of using consensus estimates reported by TCGA, 785 out of 901 cases (~ 88%) would be rescued, avoiding using a consensus purity that contains an error that is larger than 5%. Overall, this shows that CNAqc can be used to select among multiple purity estimates even from WES, avoiding at least in principle the need of consensus calling.

#### WGS multi-region colorectal cancer

A common design of modern cancer genomics studies requires collecting multiple, spatially separated, samples from the same tumor. We have sought out to test CNAqc on previously published WGS multi-region data of primary colorectal adenocarcinomas [30, 54]. We gathered data for 2 patients, for a total of 10 samples with median coverage ~ 80 × , purity ~ 80% (Fig. 6); for these samples, we generated mutations and CNAs with Platypus [54] and Sequenza [22]. Since Sequenza can return multiple solutions to the CNA inference problem, we tested if CNAqc could select the best tumor segmentation as compared to published calls obtained by the CloneHD [26] algorithm.

Sequenza with default parameters returned a main solution close to CloneHD, proposing an alternative tetraploid solution with halved purity. We used it to re-run Sequenza, and also generated another low-purity alternative solution. We used CNAq to compare these 3 runs; for sample Set7_57 from patient Set7 (Fig. 6a) CNAqc selected the correct diploid solution with 80% purity, suggesting only a small purity adjustment that did not change the quality of the QC (Fig. 6b, 6c). Interestingly, peak detection scores from CNAqc invariably failed both the tetraploid and low-purity solutions (Fig. 6d, e), showing how CNAqc can be used to select among alternative solutions proposed by a copy number caller. An equivalent result was also obtained for 6 WGS samples of patient Set_6 (Additional file 1: Fig. S30) and, in general, CNA profiles seemed rather homogenous across all samples.

Summarizing, multi-region data is of particular interest to understand the spatial heterogeneity of a tumor, because the evolutionary history across samples will be shared, but there will be sample-specific variations (e.g., multiple non-normal CNA states). At the moment, CNAqc can only treat every sample as independent, as we show in this example, and in the future it would be interesting to extend the framework to model explicitly multi-region datasets. 

### Supplementary Information

**Additional file 1: Figure S1.** CNA segments in PCAWG.** Figure S2.** Chromosome-level analysis of a PCAWG sample. **Figure S3.** Subclonal CNA modelling.** Figure S4.** Peak detection algorithm.** Figure S5.** Wall-clock time. **Figure S6.** Synthetic tests for coverage and purity values: fails proportion. **Figure S7.** Synthetic tests for coverage and purity values: CCFs. **Figure S8.** Automatic calibration testing. **Figure S9.** Single-cell low-pass DNA copy numbers. **Figure S10.** Peak analysis from single-cell pseudobulk. **Figure S11.** Single-cell correction errors by CNAqc. **Figure S12.** Single-cell pseudo-bulk clonal and subclonal CNAs. **Figure S13.** Single-cell subclonal mirrored allelic imbalance. **Figure S14.** Single-cell subclonal mirrored allelic imbalance: analysis. **Figure S15.** Single-cell multiplicity phasing. **Figure S16.** CCF comparison with Ccube: overall results. **Figure S17.** CCF comparison with Ccube: example differences. **Figure S18.** PCAWG low-mutational burden sample. **Figure S19.** PCAWG high purity sample 1/2. **Figure S20.** PCAWG high purity sample 2/2. **Figure S21.** PCAWG evolution model for subclonal CNAs. **Figure S22.** PCAWG evolution model for subclonal CNAs: overall counts. **Figure S23.** Example TCGA cases. **Figure S24.** Example TCGA cases: multiple purity comparison. **Figure S25.** Overall TCGA analysis statistics. **Figure S26.** Overall TCGA analysis statistics: purity scatter. **Figure S27.** Sequenza-CNAqc pipeline for CNA calling. **Figure S28.** PCAWG samples with complex CNAs. **Figure S29.** Colorectal multi-region data analysis. **Figure S30.** Colorectal multi-region data analysis: peaks.

**Additional file 2. **Review history.

## Data Availability

PCAWG calls are publicly available at the ICGC Data Portal  [63] ; we used the following files: • Somatic consensus SNVs and indels (simple_somatic_mutation.aggregated.vcf). • Somatic allele-specific CNAs (consensus.20170119.somatic.cna.annotated.tar.gz). • Purity and ploidy cohort table (consensus.20170217.purity.ploidy.txt.gz). TCGA calls are publicly available at the GDC Data Portal  [65]. CNAqc outputs from PCAWG and TCGA analyses are available at Zenodo  [66]. Multi-region colorectal cancer are available at GitHub as VCF and TSV files  [67]. Raw data is deposited in EGA under accession number EGAS00001003066. Data from Genomics England  [35] were analyzed within the Genomics England Research Environment secure data portal, under Research Registry project “Quality Control of somatic calls from whole-genome sequencing” (RR-878). These are made available in the project folder of the Research Environment, under root “/published_data_archive/”. The Genomics England data set can be accessed by joining the community of academic and clinical scientists via the Genomics England Clinical Interpretation Partnership, following the instructions available at https://www.genomicsengland.co.uk/about-gecip/. CNAqc is an open source R package hosted at https://caravagnalab.github.io/CNAqc/. The tool webpage contains vignettes and manuals, and the release used in this manuscript is available at  [68] along with the source code used to perform the analysis and produce the figures described in the text.
