# Supplementary material for: Computational validation of clonal and subclonal copy number alterations from bulk tumor sequencing using CNAqc
Source: Genome Biol. 2024 Jan 31;25:38. doi: 10.1186/s13059-024-03170-5 (PMC10832148; doi:10.1186/s13059-024-03170-5)
Supplement: Supplementary file 1 — Additional file 1: Figure S1. CNA segments in PCAWG. Figure S2. Chromosome-level analysis of a PCAWG sample. Figure S3. Subclonal CNA modelling. Figure S4. Peak detection algorithm. Figure S5. Wall-clock time. Figure S6. Synthetic tests for coverage and purity values: fails proportion. Figure S7. Synthetic tests for coverage and purity values: CCFs. Figure S8. Automatic calibration testing. Figure S9. Single-cell low-pass DNA copy numbers. Figure S10. Peak analysis from single-cell pseudobulk. Figure S11. Single-cell correction errors by CNAqc. Figure S12. Single-cell pseudo-bulk clonal and subclonal CNAs. Figure S13. Single-cell subclonal mirrored allelic imbalance. Figure S14. Single-cell subclonal mirrored allelic imbalance: analysis. Figure S15. Single-cell multiplicity phasing. Figure S16. CCF comparison with Ccube: overall results. Figure S17. CCF comparison with Ccube: example differences. Figure S18. PCAWG low-mutational burden sample. Figure S19. PCAWG high purity sample 1/2. Figure S20. PCAWG high purity sample 2/2. Figure S21. PCAWG evolution model for subclonal CNAs. Figure S22. PCAWG evolution model for subclonal CNAs: overall counts. Figure S23. Example TCGA cases. Figure S24. Example TCGA cases: multiple purity comparison. Figure S25. Overall TCGA analysis statistics. Figure S26. Overall TCGA analysis statistics: purity scatter. Figure S27. Sequenza-CNAqc pipeline for CNA calling. Figure S28. PCAWG samples with complex CNAs. Figure S29. Colorectal multi-region data analysis. Figure S30. Colorectal multi-region data analysis: peaks. [file 13059_2024_3170_MOESM1_ESM.pdf]

Computational validation of clonal and subclonal copy number alterations from bulk tumour sequencing using CNAqc

Alice Antonello, Riccardo Bergamin, Nicola Calonaci, Jacob Househam, Salvatore Milite, Marc J Williams, Fabio Anselmi, Alberto d'Onofrio, Vasavi Sundaram, Alona Sosinsky, William CH Cross, Giulio Caravagna

Additional File 1: Supplementary Figures

- Figure S1. *CNA segments in PCAWG.*
- Figure S2. *Chromosome-level analysis of a PCAWG sample.*
- Figure S3. *Subclonal CNA modelling.*
- Figure S4. *Peak detection algorithm.*
- Figure S5. *Wall-clock time.*
- Figure S6. *Synthetic tests for coverage and purity values: fails proportion.*
- Figure S7. *Synthetic tests for coverage and purity values: CCFs.*
- Figure S8. *Automatic calibration testing.*
- Figure S9. *Single-cell low-pass DNA copy numbers.*
- Figure S10. *Peak analysis from single-cell pseudobulk.*
- Figure S11. *Single-cell correction errors by CNAqc.*
- Figure S12. *Single-cell pseudo-bulk complex and subclonal CNAs.*
- Figure S13. *Single-cell mirrored subclonal imbalance.*
- Figure S14. *Single-cell mirrored subclonal imbalance: analysis.*
- Figure S15. *Single-cell multiplicity phasing.*
- Figure S16. *CCF comparison with Ccube: overall results.*
- Figure S17. *Figure S16. CCF comparison with Ccube: example differences.*
- Figure S18. *PCAWG low-mutational burden sample.*
- Figure S19. *PCAWG high purity sample 1/2.*
- Figure S20. *PCAWG high purity sample 2/2.*
- Figure S21. *PCAWG evolution model for subclonal CNAs.*
- Figure S22. *PCAWG evolution model for subclonal CNAs: overall counts.*
- Figure S23. *Example TCGA cases.*
- Figure S24. *Example TCGA cases: multiple purity comparison.*
- Figure S25. *Overall TCGA analysis statistics.*
- Figure S26. *Overall TCGA analysis statistics: purity scatter.*
- Figure S27. *Sequenza-CNAqc pipeline for CNA calling.*
- Figure S28. *PCAWG samples with complex CNAs.*
- Figure S29. *Colorectal multi-region data analysis.*
- Figure S30. *Colorectal multi-region data analysis: peaks.*

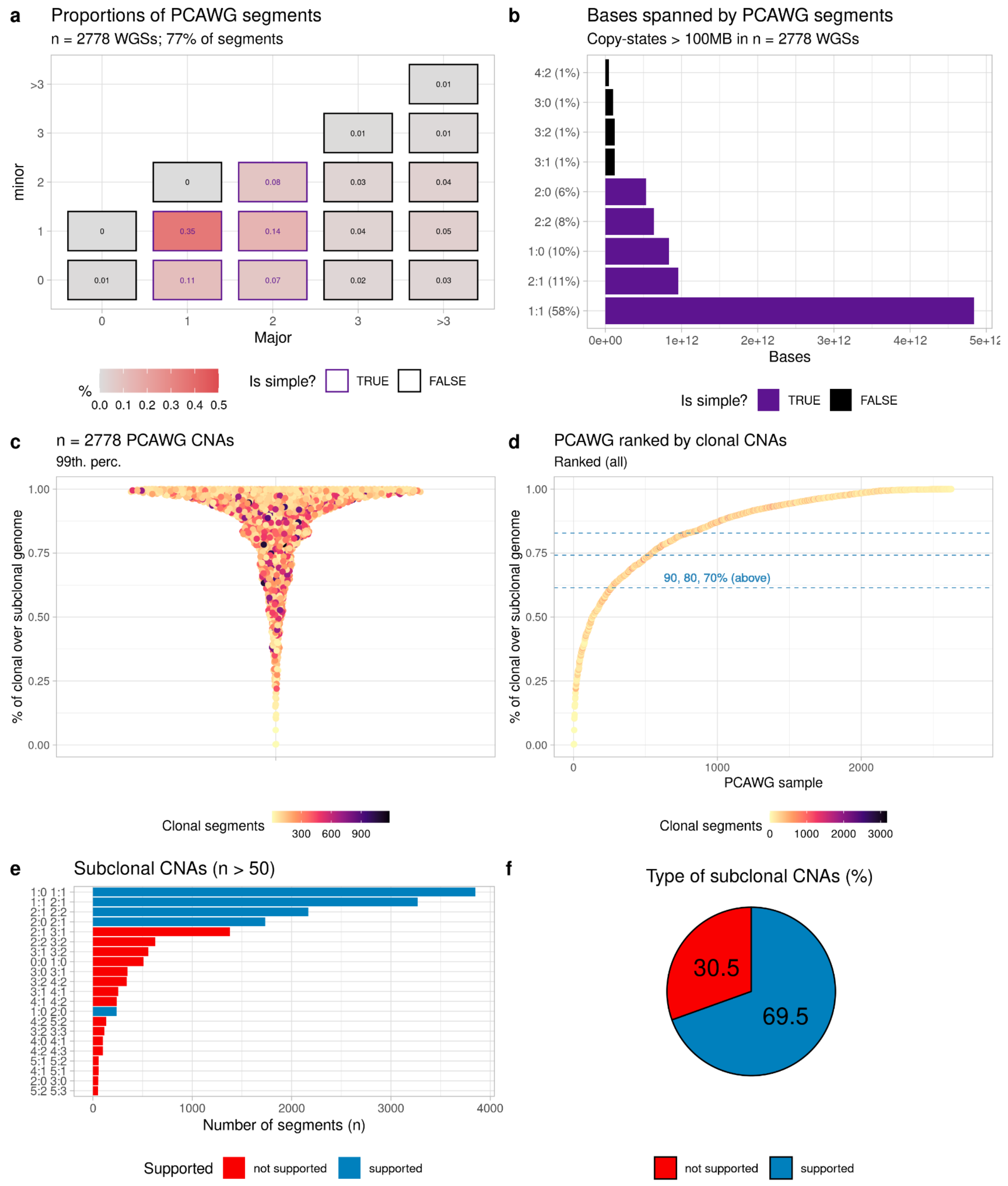

Figure S1. CNA segments in PCAWG. a. Proportion of PCAWG CNA segments split by copy state, obtained by consensus calling across multiple callers with $n = 2778$ WGS samples of primary tumours. The matrix reports major and minor alleles, the colour and number reflects the proportion of CNA segments with that copy state across total (e.g., 36% of segments are diploid heterozygous, 1:1). CNA segments used by CNAqc are coloured in purple; in total, 78% of the overall set of segments (>600,000) can be processed by our method (36% of segments are 1:1, 15% are 2:1, 11% are 1:0, 8% are 2:2 and 8% are 2:0). **b.** Number of bases covered, and proportions relative to the total genome spanned by all the PCAWG segments in panel (a). Diploid heterozygous segments cover over a thousand billion bases($> 10^{12}$), accounting for 58% of the genome covered by these segments. The segments supported by CNAqc are the top-5 most common segments reported across all PCAWG, covering 93% of all bases sequenced in this cohort. **c.** Battenberg clonal and subclonal CNAs available in PCAWG. To simplify the visualisation we remove outliers exceeding the 99-th

quantile of the data distribution. Every dot is the percentage of the tumour genome spanned by clonal segments, coloured by the number of segments per sample. So if a sample has >50% of clonal segments it is above the horizontal dashed line. **d.** We rank by sorting the percentages shown in panel (c) to note that only $n = 124$ PCAWG samples (vertical dashed red line) have more subclonal than clonal CNAs. **e.** Number of CNA segments covered by subclonal copy number events, divided by karyotype. The plot shows how the most common subclonal events involving “simple” karyotypes are supported by CNAqc. As in panels (c and d), we consider a segment subclonal if the CCF provided by Battenberg is lower than 1, and clonal otherwise. As such subclonal structure is limited to 2 clones **f.** Percentage of subclonal segments supported by CNAqc. The number calculation is done on the number shown in panel (e).

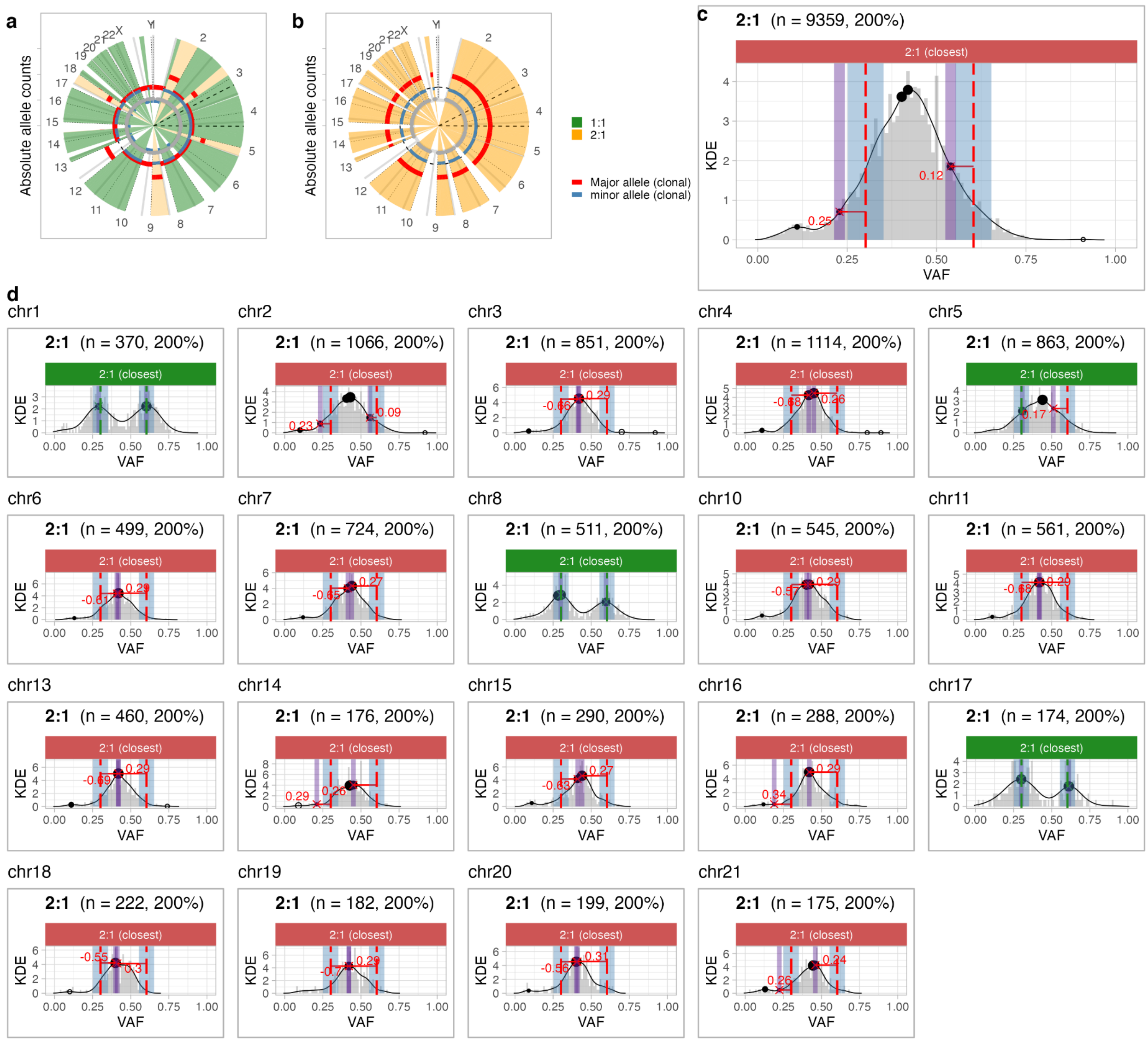

Figure S2. Chromosome-level analysis of a PCAWG sample. a-b. Chromosome-level analysis of an artificially miscalled sample, obtained from the PCAWG hepatocellular carcinoma sample ca5ded1c-c622-11e3-bf01-24c6515278c0. From (a) the true copy-number profile called on this sample we simulate (b) a wrong profile in which the whole genome is called triploid. **c.** The incorrect calls are detected by the peak analysis performed by CNAqc on the whole genome, which correctly fails the test and suggests a correction on the sample purity estimate. **d.** By peak analyses at the level of individual chromosomes it is easy to identify those portions of the genome whose copy state has been incorrectly called triploid.

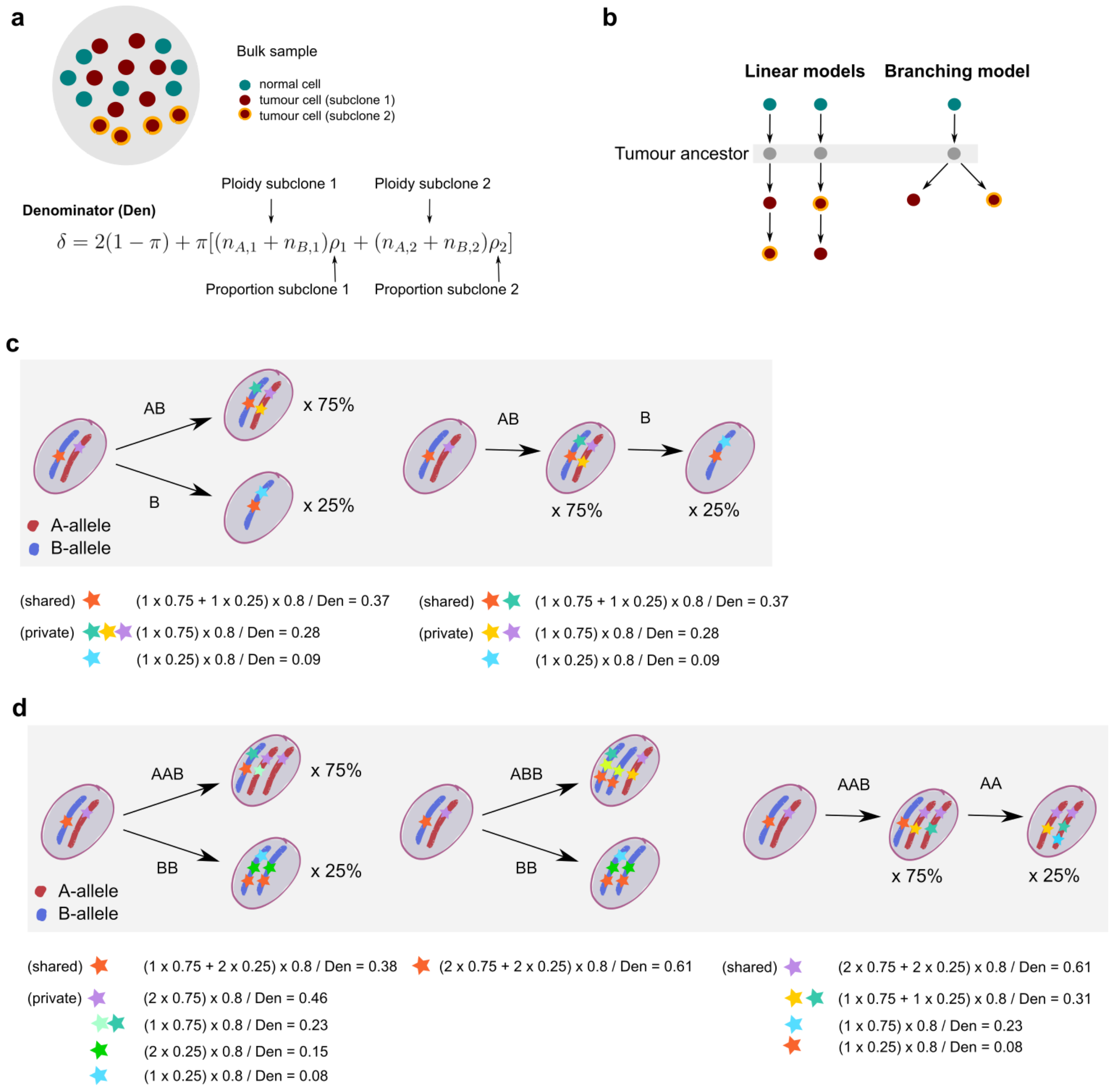

Figure S3. Subclonal CNA modelling. a. Tumour bulk sample composed of both normal and tumour cells belonging to two distinct subclones. In order to compute all possible expected VAF peaks, one needs to account for the multiplicities of the variants and weight them according to the proportion of normal reads $(2(1-\pi))$ and reads coming from the first and the second subclone $\pi[(n_{A1} + n_{Bi})\rho_1 + (n_{A1} + n_{B2})\rho_2]$. **b.** Linear and branching models representing the possible evolutionary paths explaining the formation of the observed subclones. **c.** Linear and branching model for subclones 1:0 and 1:1 with ancestral diploid state, and computation of the expected VAF peaks. Although the variant groups are different between the two evolutionary paths, it is not possible to distinguish them from data as the expected VAF peaks are the same. **d.** Like in panel (c) but for subclones 2:0 and 2:1. In this case, both the number of peaks and their expected position change according to the sequence of events that led to the formation of the subclones. Note the difference in the VAF peak for shared mutations in two possible branching models (AAB | BB and ABB | BB).

<u>Algorithm:</u> *Peak detection in CNAqc*

Input: Mutations, allele-specific CNA segments, purity,
Parameters: purity error tolerance $\epsilon > 0$ and VAF tolerance $\sigma > 0$

- set $K = \{1:0,\ 1:1,\ 2:0,\ 2:1,\ 2:2\}$, where $n_A : n_B$ are the copies of the Major/ minor alleles;

# Peak detection for every copy state

- for every copy state $k \in K$ and multiplicity $m \in \{1, 2\}$:
  - retain mutations M_k mapping to segments with copy state k;
  - compute v_m with equation (1);
  - compute Δv_m with equation (1.2);
  - determine peaks $d_1, ..., d_n$ from the VAF distribution of M_k;
  - match d_*^m to v_m by either closest or rightmost hit;
  - define interval I_m from v_m and Δv_m with equation (1.6);
  - define I_m^{VAF} from d_*^m and σ with equation (1.6);
  - define PASS for k and m if $|I_m^{VAF} \cap I_m| > 0$, FAIL otherwise;
  - determine the number n_m of mutations mapping below VAF peak d_*^m;
  - if $k \in \{2:0,\ 2:1,\ 2:2\}$, compare n_m across peaks and define the status for k from the largest n_m, otherwise use the only available peak;

# Sample level quality control status

- for all copy states $k \in K$, define w_k by normalising the number of mutations mapped to the copy state, and rescale w_k by 2 if $k \in \{2:0,\ 2:1,\ 2:2\}$;
- for every copy state $k \in K$ define $\lambda_{k,}^{PASS}$ and $\lambda_{k,}^{FAIL}$ with equations (7) and (8);
- define the sample score λ with equation (9), and evaluate the sample status from the sum of $\lambda_{k,}^{PASS}$ and $\lambda_{k,}^{FAIL}$ for all $k \in K$, taking the largest.

Figure S4. Peak detection algorithm. Pseudocode of the peak detection algorithm and quality control strategy in CNAqc (Online Methods) that apply to simple clonal CNAs in order to determine purity adjustments for a copy number caller, and sample level QC metrics.

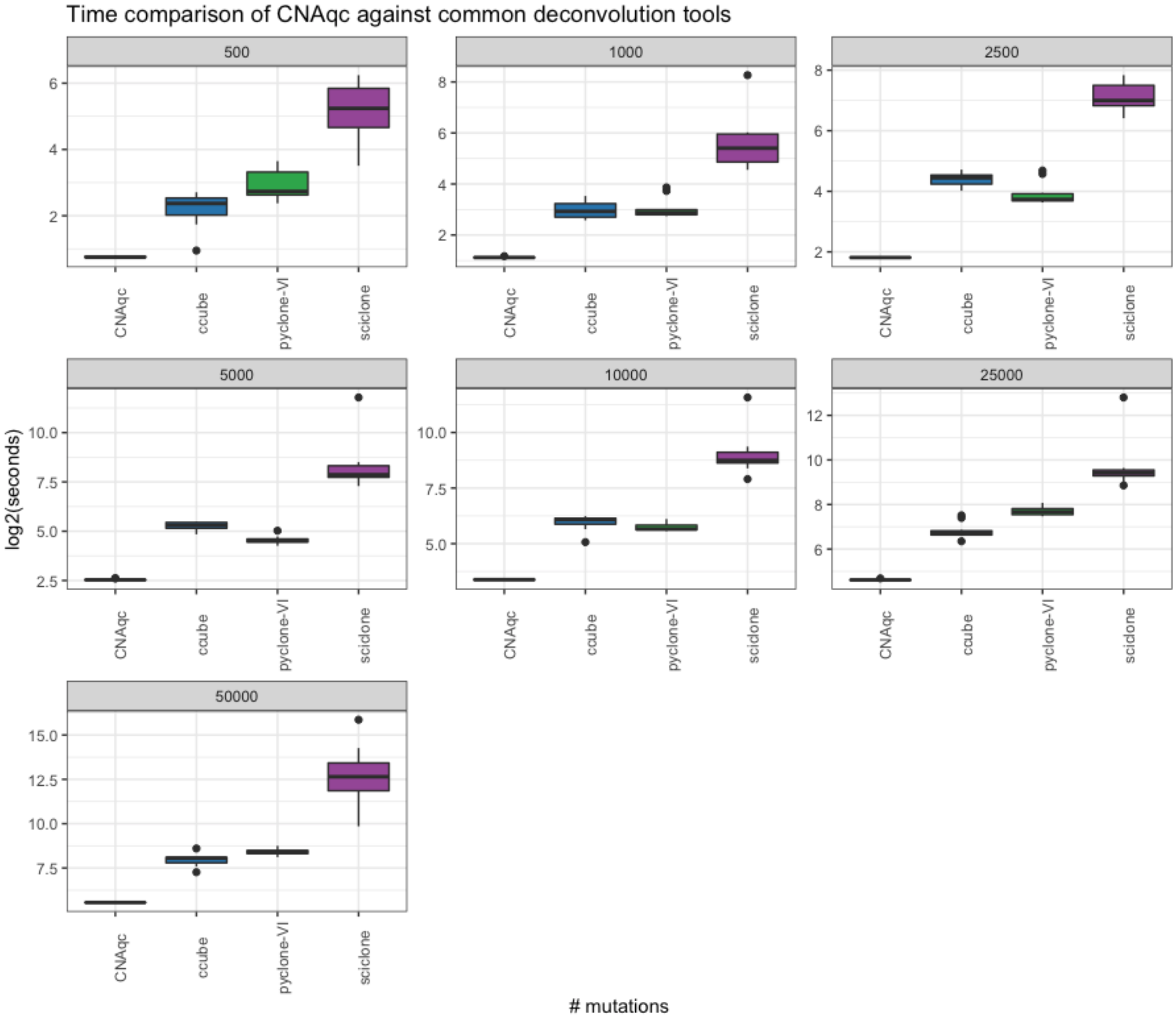

Figure S5. Wall-clock time. Computational time of CNAqc, compared with common subclonal deconvolution tools (Ccube, Pyclone-VI and Sciclone) on datasets with 500, 1000, 2500, 5000, 10000, 25000 rr 50000 mutations. The CNAqc peak detection algorithm is extremely fast and preprocesses even 50000 mutations in less than a minute (~47 seconds). The fastest deconvolution tools are Pyclone-VI and Ccube, both implemented using Variational Inference; Sciclone drops rapidly in performance as the number of SNVs increases. Time is reported in log2(seconds).

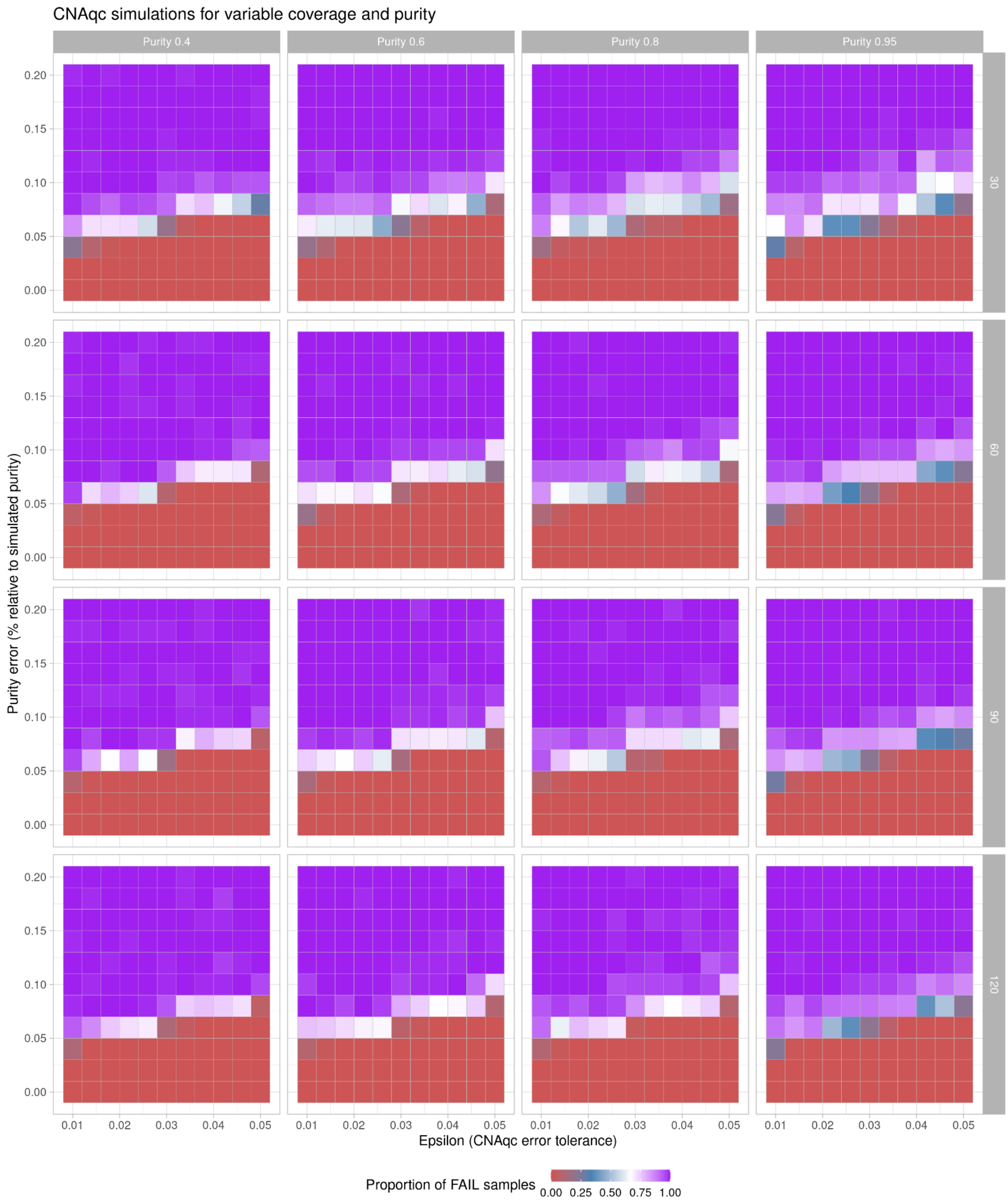

Figure S6. Synthetic tests for coverage and purity values: fails proportion. CNAqc tests on synthetic tumours generated with different coverage and purity. We report the proportion of rejected samples running the tools with an error on the simulated purity (y-axis), and a tolerance to match peaks (x-axis).

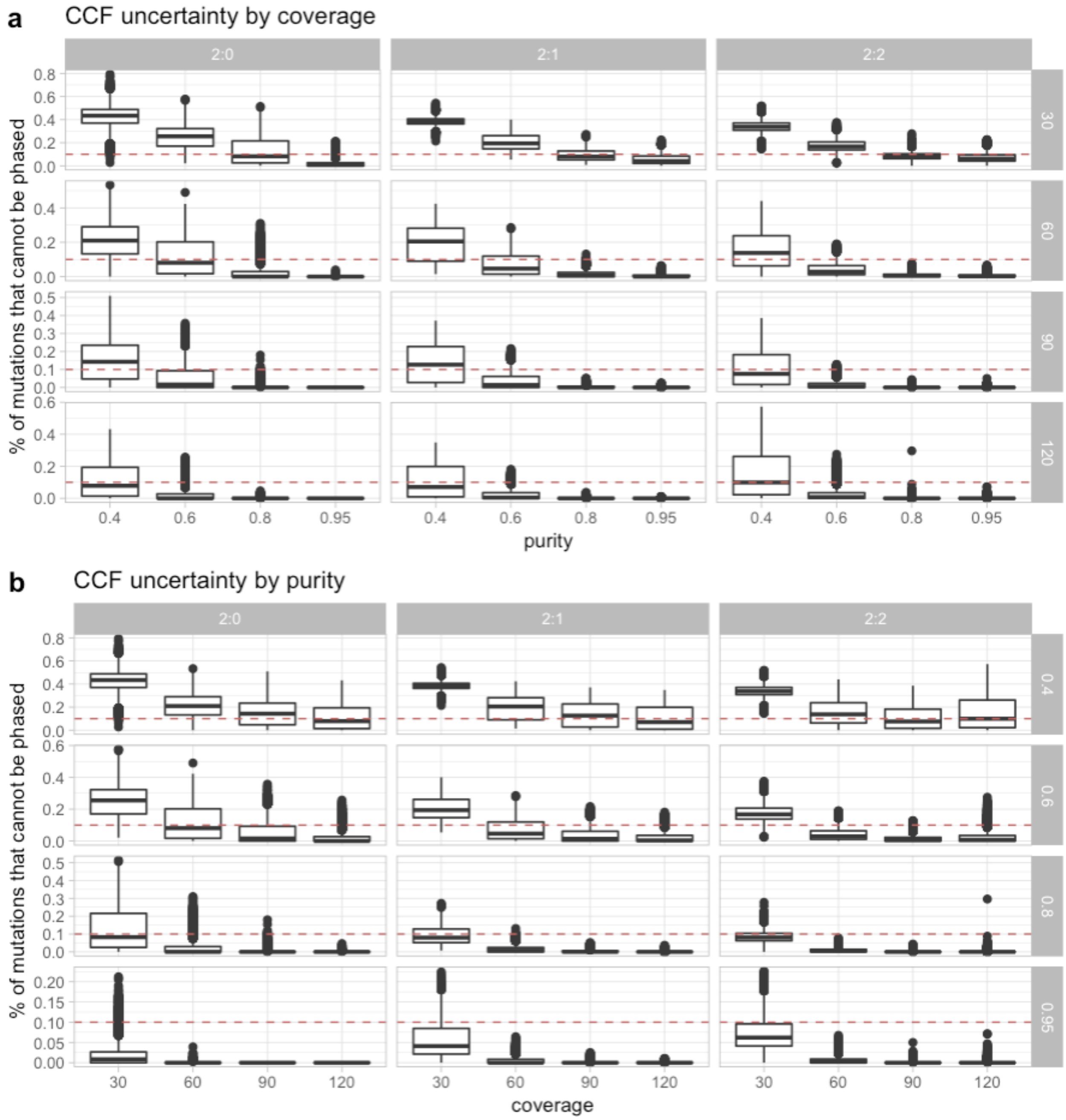

Figure S7. Synthetic tests for coverage and purity values: CCFs. a. For the simulated tumours in Supplementary Figure S8, we report the proportion of mutations for which CNAqc does assign a CCF (uncertain in phasing multiplicities), as a function of purity at fixed coverage values. The dashed line at 10% is the default parameter value to determine the final PASS or FAIL status per copy state. **b.** As in panel (a), but fixing purity.

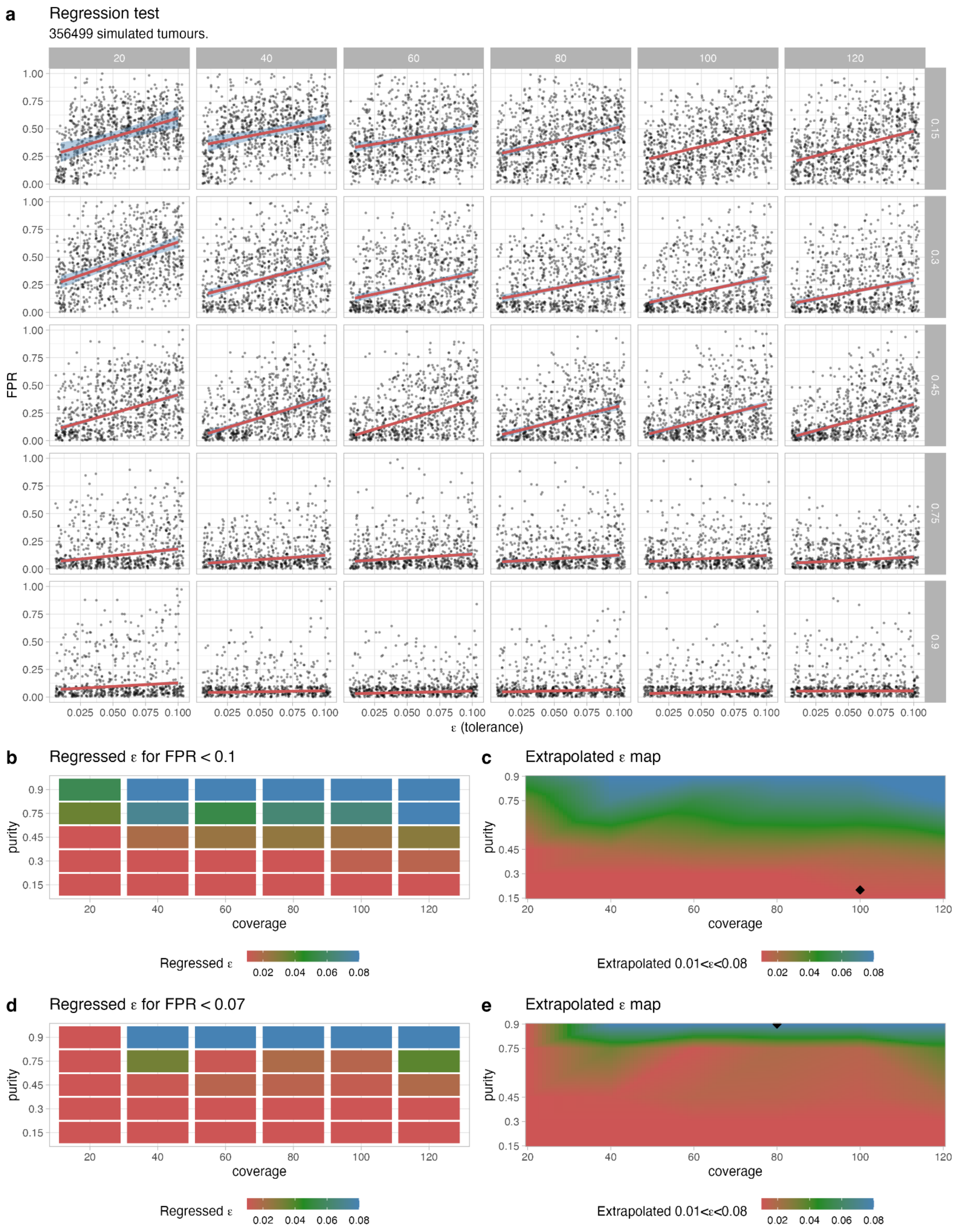

Figure S8. Automatic calibration testing. a. Calibration test for CNAqc, trying to determine the best input ϵ as a function of purity and coverage with 100 distinct tumour segmentations, where for each ϵ in each panel we generate 10 datasets. To every dataset we apply a random uniform noise in the range $[\epsilon; \epsilon + 3\%]$, i.e., around the boundary of acceptability; we seek to determine if the tool, with ϵ in input, can flag the sample as FAIL. The panels show false positives rates (FPR) from PASS samples that should be instead failed, and regressed performance (red line). As expected, for low

purity/coverage we observe higher FPR. **b,c.** For every input value of purity (passed by the user) and coverage (obtained from data), we invert the regression fit in panel (a) for a given value of FPR (target 10% in this panel). At this point, we can extrapolate ϵ to the full range of trained values; for a 100x sample with 20% purity one should run CNAqc with $\epsilon = 0.01$ in order to keep the FPR below 10%. This makes sense since at low purity noise in peak detection can confound the tool which would pass peaks that should not be passed. **d, e.** Same as in panels (b,c), with different parameters (FPR < 0.07; coverage 80x, purity 90%).

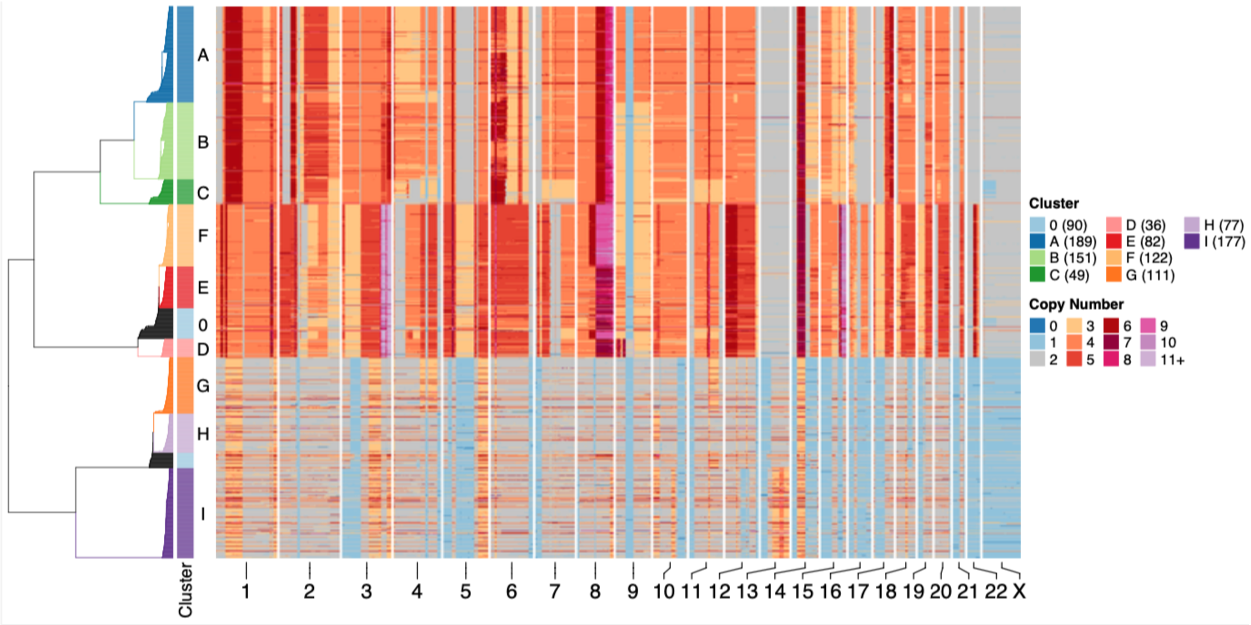

Figure S9. Single-cell low-pass DNA copy numbers. Single-cell UMAP [61] clusters obtained from low-pass single-cell data of 1084 cells from an ovarian cancer assay. These data are generated by using the DLP+ library preparation protocol [31]. Upon calling of cell-level allele-specific CNAs, cells are clustered into several groups that define CNA-level clones.

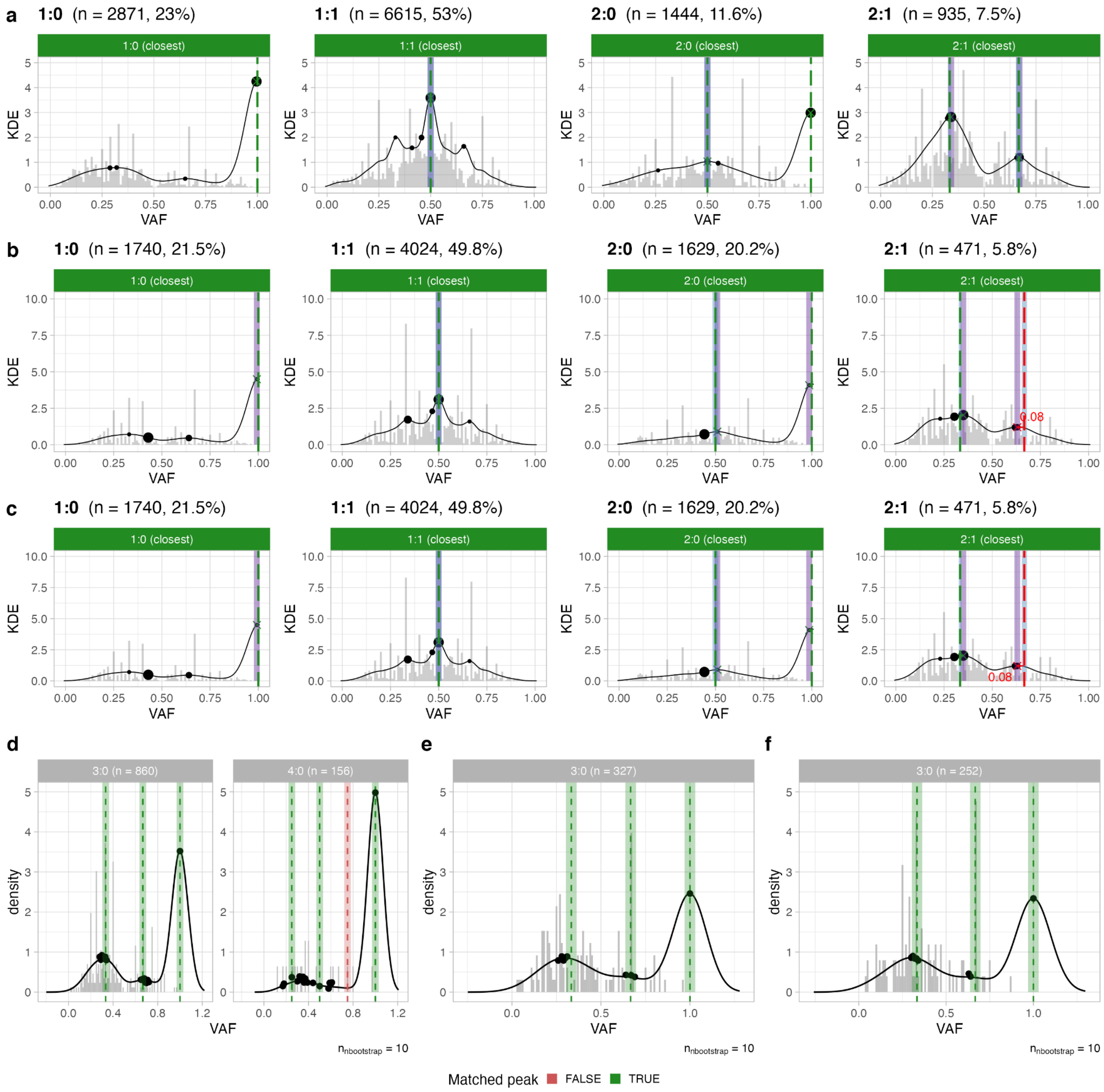

Figure S10. Peak analysis from single-cell pseudobulk. a-c. Analysis of simple CNAs obtained from pseudobulk data for clusters I (177 cells) , G (111 cells) and H (77 cells) from Supplementary Figure S7. These tumours are 100%-pure populations by construction, and CNAqc can validate the generated calls at both the level of somatic mutations and copy numbers, despite the small number of mutations and noisy VAF profile from single-cell data. **d-f.** CNAqc peak analysis of more rare CNAs from the clones in panels (a-c); LOH calls with 3 or 4 total alleles are matched by CNAqc, therefore validating the peak detection algorithms in the tool.

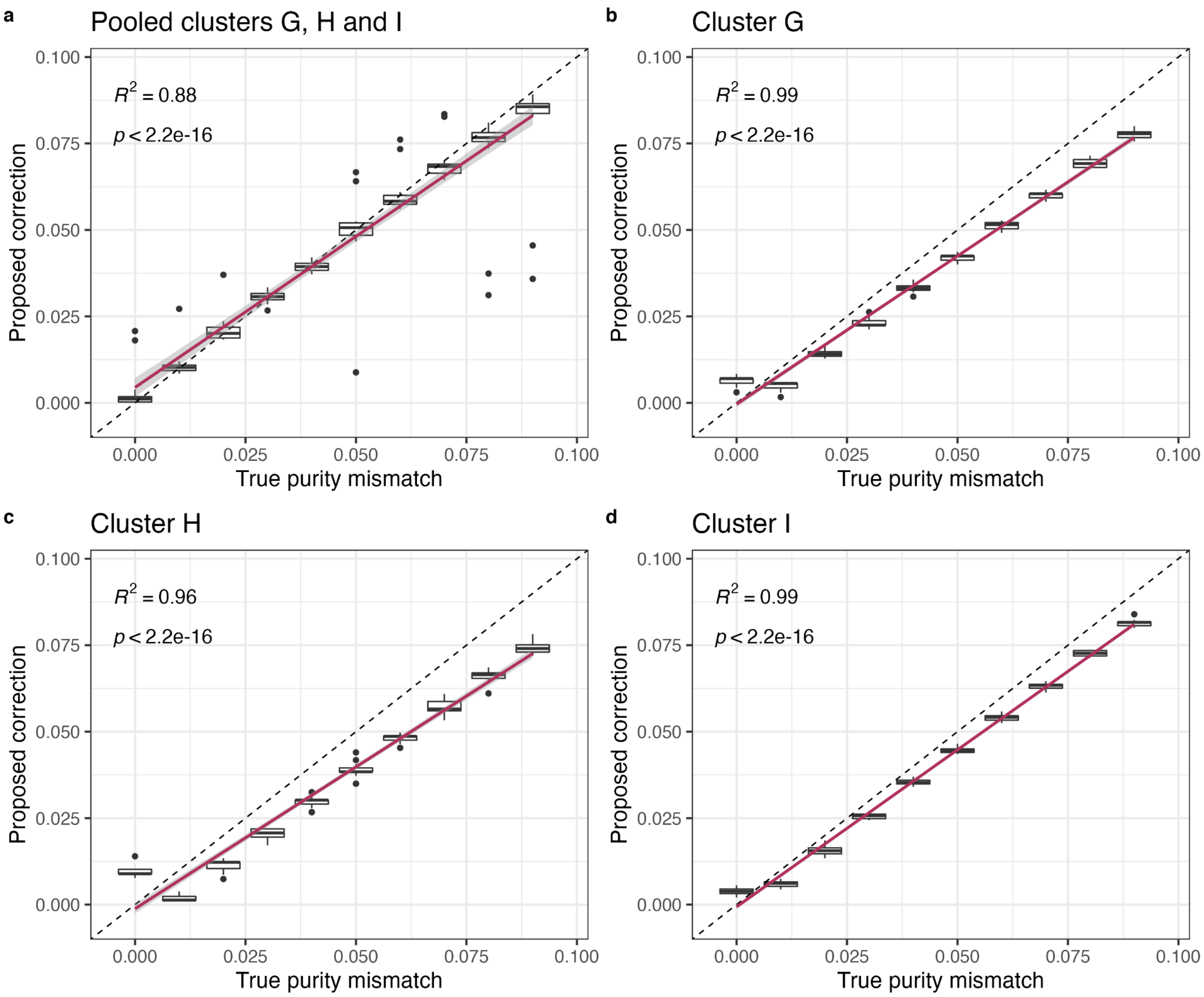

Figure S11. Single-cell correction errors by CNAqc. a-d. Validating the accuracy of the correction on sample purity proposed by CNAqc using low-pass single cell data. The tests are performed from monoclonal pseudo-clones generated from clusters of single-cells with the same clonal copy number profiles, and restricted to genome regions with loss of heterozygosity, where the VAF profile is less noisy and each sample is 100% pure by construction. Peak analysis is run multiple times for each cluster, upon decreasing input sample purity from 100% to 90% with 1% step and 15 repetitions per point. The tested samples from Supplementary Figure S7 are (a) a pileup of clonal CNAs common to clusters G, H and I (also used in Supplementary Figure S10), in 1:0 and 2:0 regions, (b) cluster G restricted to 1:0 segments, (c) cluster H restricted to 1:0 segments and (d) cluster I restricted to 1:0 and 2:0 segments. In every case the proposed correction is in agreement with the input mismatch, the correlation coefficient is $0.88 < R^2 < 0.99$ with p-value $p < 2.2e\text{-}16$.

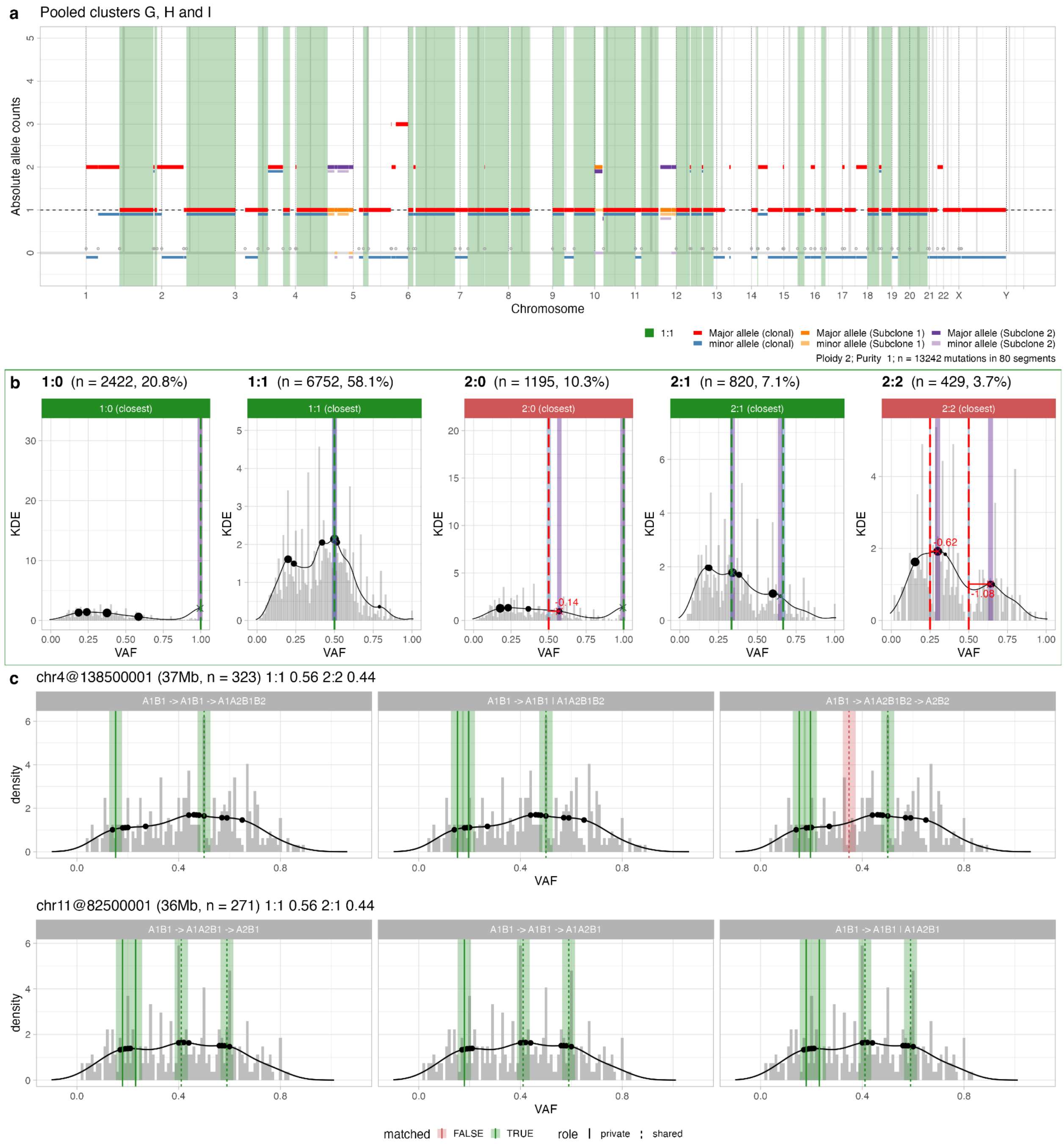

Figure S12. Single-cell pseudo-bulk complex and subclonal CNAs. a. Pseudo-clone generated by merging low-pass sequencing data for cells from clusters G, H and I in Supplementary Figure S7. Here, we retained clonal CNAs common to clusters G, H and I, and identified segments where clusters H and I have a CNA that differs from cluster G. Then, we mixed all the cells (111 for G, 77 for H and 177 for I), obtaining a mixture with ~70% cells from merged cluster H+I, and ~30% from cluster G. **b.** QC of the clonal CNAs for the sample, using purity 100%; CNAqc passess the sample. **c.** QC of 2 subclonal CNAs for the sample, harbouring >250 SNVs each. CNAqc validates the required VAF peaks, therefore supporting the presence of subclonal CNAs in these data. The model cannot distinguish branching versus linear dynamics because, in these VAFs, peaks are found - and matched - at very similar frequencies. For example the bottom panel A1B1→A1A2B1 | A2B1 denotes a branching ("|") model with 2:1 (A1A2B1) and 1:1 (A2B1) siblings, while A1B1→A1B1→A1A2B1 denotes a linear ("→") model with 1:1 (A1B1) ancestral to 2:1 (A1A2B1).

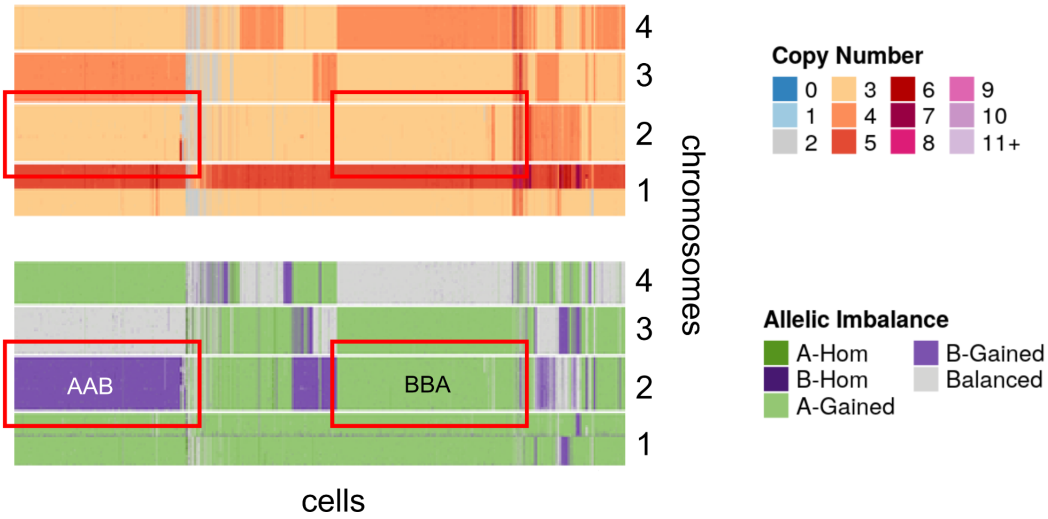

Figure S13. Single-cell mirrored subclonal imbalance. Low-pass copy number data obtained with SIGNAL, from data released in [33]. The heatmaps show total copy number for chromosomes 1 to 4, as well as phased alleles. Note that chromosome 1 is monoclonal, whereas 2-4 are defined by 2 clones. On chromosomes 3 and 4 the populations are triploid and tetraploid; on chromosome 2 they are triploid with mirrored allelic imbalance (i.e., genotypes AAB and ABB).

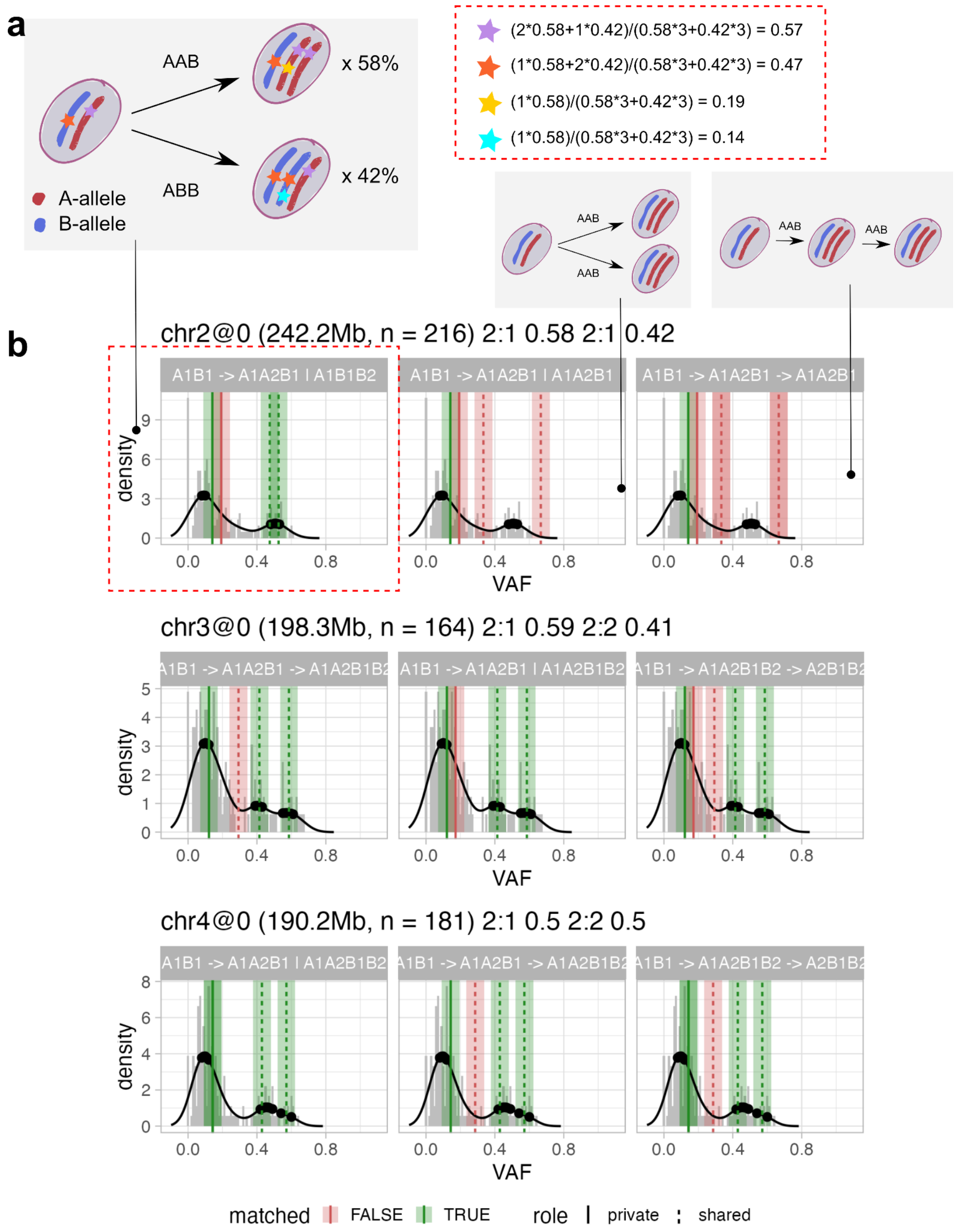

Figure S14. Single-cell mirrored subclonal imbalance: analysis. a. The CNAqc evolutionary model for two 2:1 subclones (Supplementary Figure S11) with genotypes AAB and ABB and proportions 58%/42% predicts two peaks close to 50%, and two below 20%. **c.** From a pileup of data in Supplementary Figure S7 we perform validation of subclonal CNAs with CNAqc. The tool passes most peaks across all all the considered chromosomes, for a variety of possible linear and branching evolutionary models. Note that for chromosome 2 the tool can also identify the true evolutionary model where genotypes AAB and ABB (allelic imbalance), branch from an ancestral heterozygous diploid state (genotype AB, or 1:1).

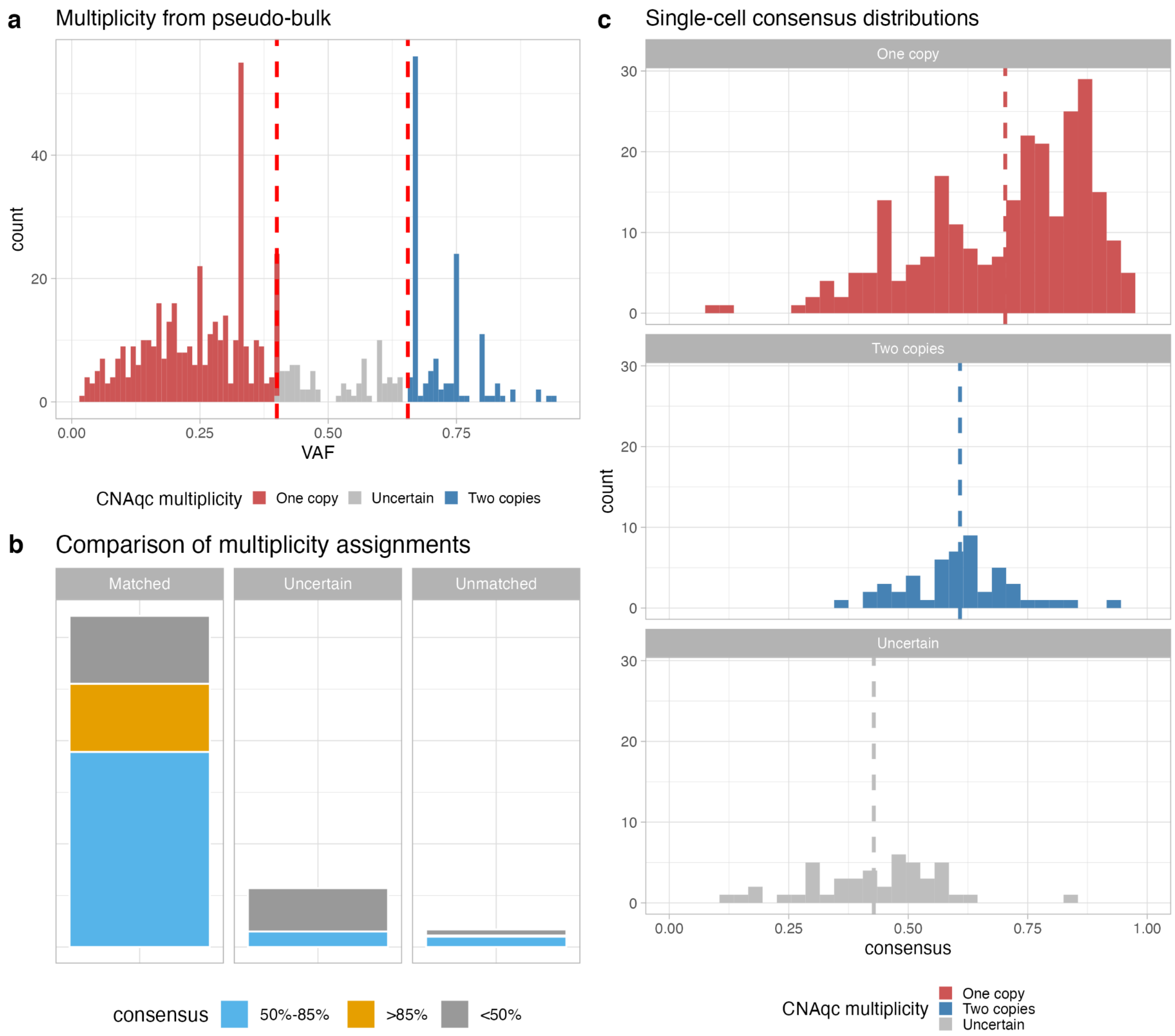

Figure S15. Single-cell multiplicity phasing. a. CNAqc multiplicity assignments from pseudo-bulk VAFs of mutations on 2:1 segments against consensus-based assignments from single cells. Test carried out with cells from cluster A from Supplementary Figure S7. Single-cell multiplicities are set to $m = 1$ when the majority of single cells have VAFs closer to 0.33 rather than to 0.66, and are assigned $m = 2$ otherwise. Here CNAqc identifies mutations for which VAFs are uncertain to phase (central cluster). **b.** Most CNAqc multiplicity assignments from pseudo-bulk match the assignments based on consensus of single-cells, especially when the consensus is > 85% (high); a small part are unmatched but have intermediate consensus < 85% (but above 50%); most mutations with <50% consensus among single cells are flagged as uncertain to phase by CNAqc as expected. **c.** Mutations that CNAqc phases as either in one or two copies have large consensus (mean 70% for $m = 1$ and 61% for 2), while the ones flagged as uncertain to phase have low consensus (mean 43%) among single cells.

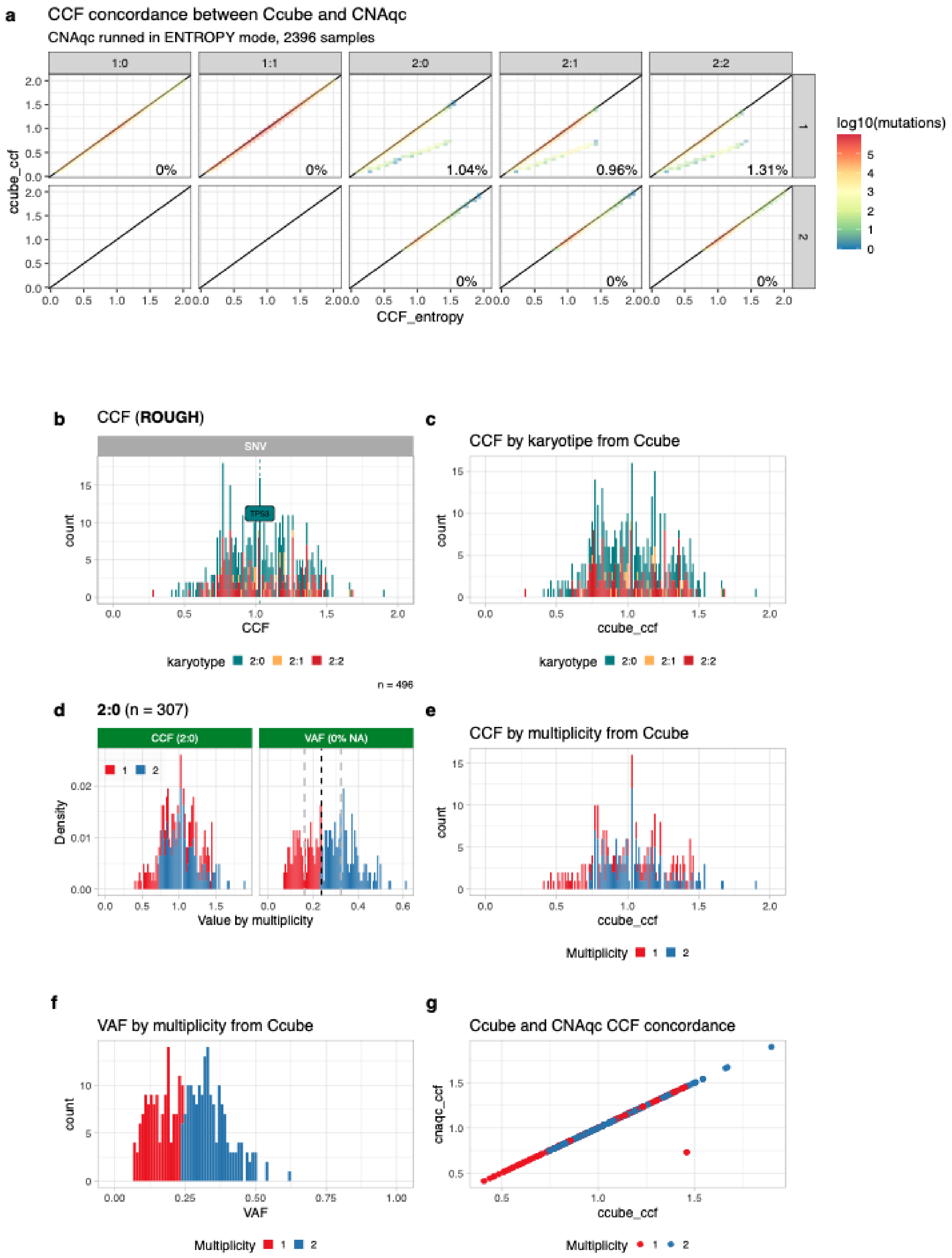

Figure S16. CCF comparison with Ccube: overall results. a. CCF calculated by CNAqc using the "entropy" method against CCF inferred by Ccube on 2396 samples from the PCAWG cohort. Results are divided by karyotype and mutation multiplicity (taking as a reference the one inferred by CNAqc),

mutations off the diagonal are discordant between the two methods. On the bottom left, the percentage of those discordant mutations over the total. **b.** CCF calculated by CNAqc using the “rough” method which assigns the multiplicity by splitting the clonal clusters at VAF level. **c.** CCF inferred by Ccube. **d.** Multiplicity assigned by CNAqc, the dashed black line depicts the splitting point to determine multiplicity. **e.** Multiplicity assigned by Ccube. **f.** VAF split by Ccube for multiplicity assignment. **g.** CCF values between Ccube and CNAqc are in almost perfect agreement (just one sample has different multiplicity). Point colour is based on the multiplicity estimated by Ccube.

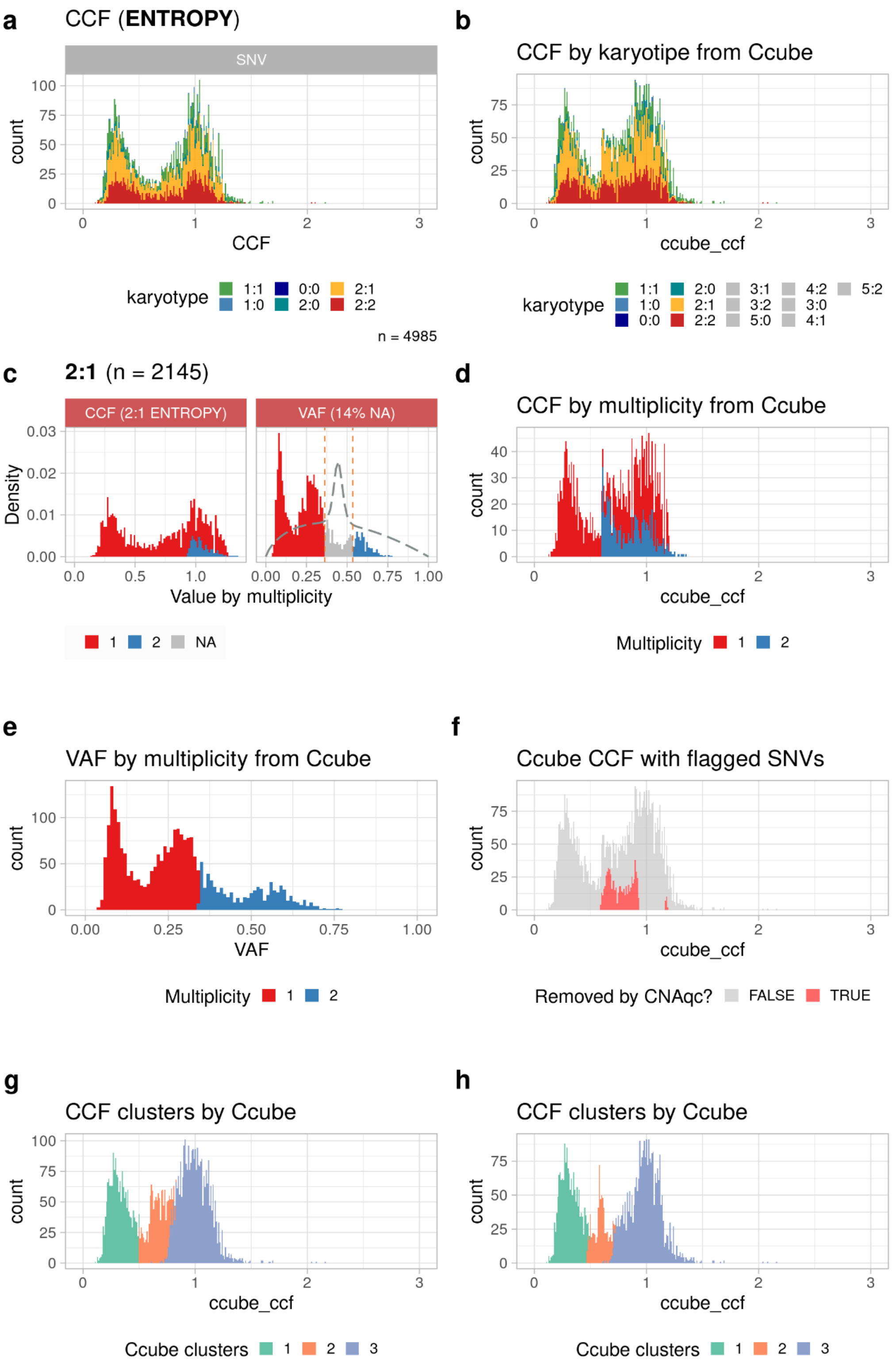

Figure S17. Figure S16. CCF comparison with Ccube: example differences. a. CCF calculated by CNAqc using the "entropy" method which discards mutations with high multiplicity uncertainty. **b.** CCF inferred by Ccube. The main difference between this profile and the one inferred by CNAqc is a bump around CCF 0.6 **c.** Multiplicity assigned by CNAqc, in grey the mutations with non-estimable multiplicity. **d.** Multiplicity assigned by Ccube, it can be noted how Ccube always assigns a definite

multiplicity value to each mutation. **e.** VAF split by Ccube for multiplicity assignment. **f.** High-entropy mutations discarded by CNAqc in the Ccube CCF profile. We clearly see the extra spike in CCF which could confound subclonal deconvolution, splitting the clonal cluster in multiple clones. **g.** Ccube recognizes the spurious peak as a subclonal cluster, as it is not able to accommodate for the overdispersion derived by the errors in multiplicity assignments with just one cluster. **h.** Even after removing the mutations with high entropy from the dataset and rerunning Ccube, we can still see a peak caused by some mutations wrongly assigned to multiplicity 2. This is consistent with the choice of CNAqc to FAIL the available CCFs for this karyotype.

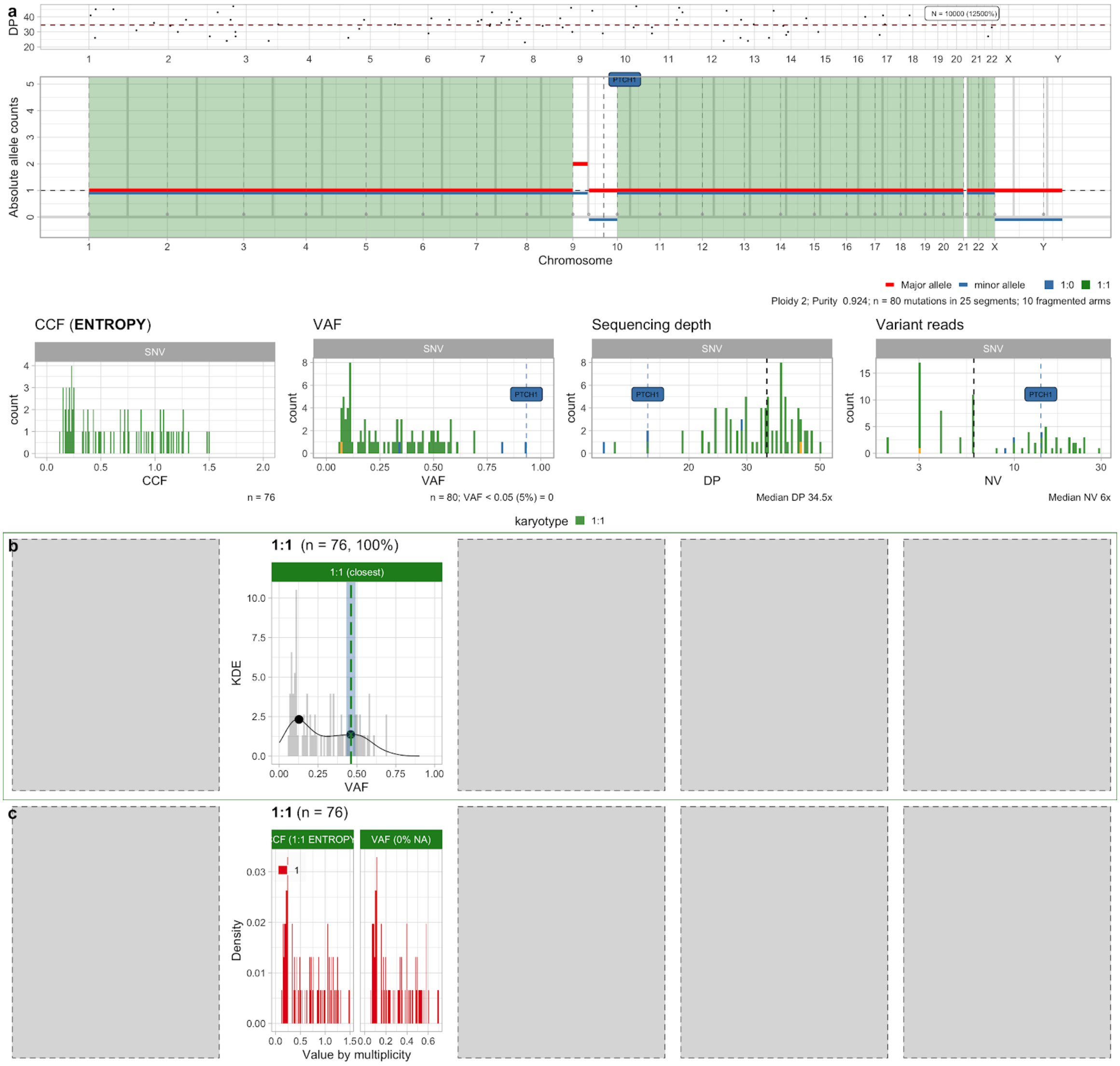

Figure S18. PCAWG low-mutational burden sample. Example PCAWG medulloblastoma sample with low-mutational burden, which passes data QC with CNAqc. **a.** Data for the sample (genome-wide CNA segments, CCF and read counts distribution). Note that this sample has only 76 SNVs in diploid tumour regions, like we observe in whole-exome assays. **b,c.** Peak analysis and CCF computation for diploid SNVs.

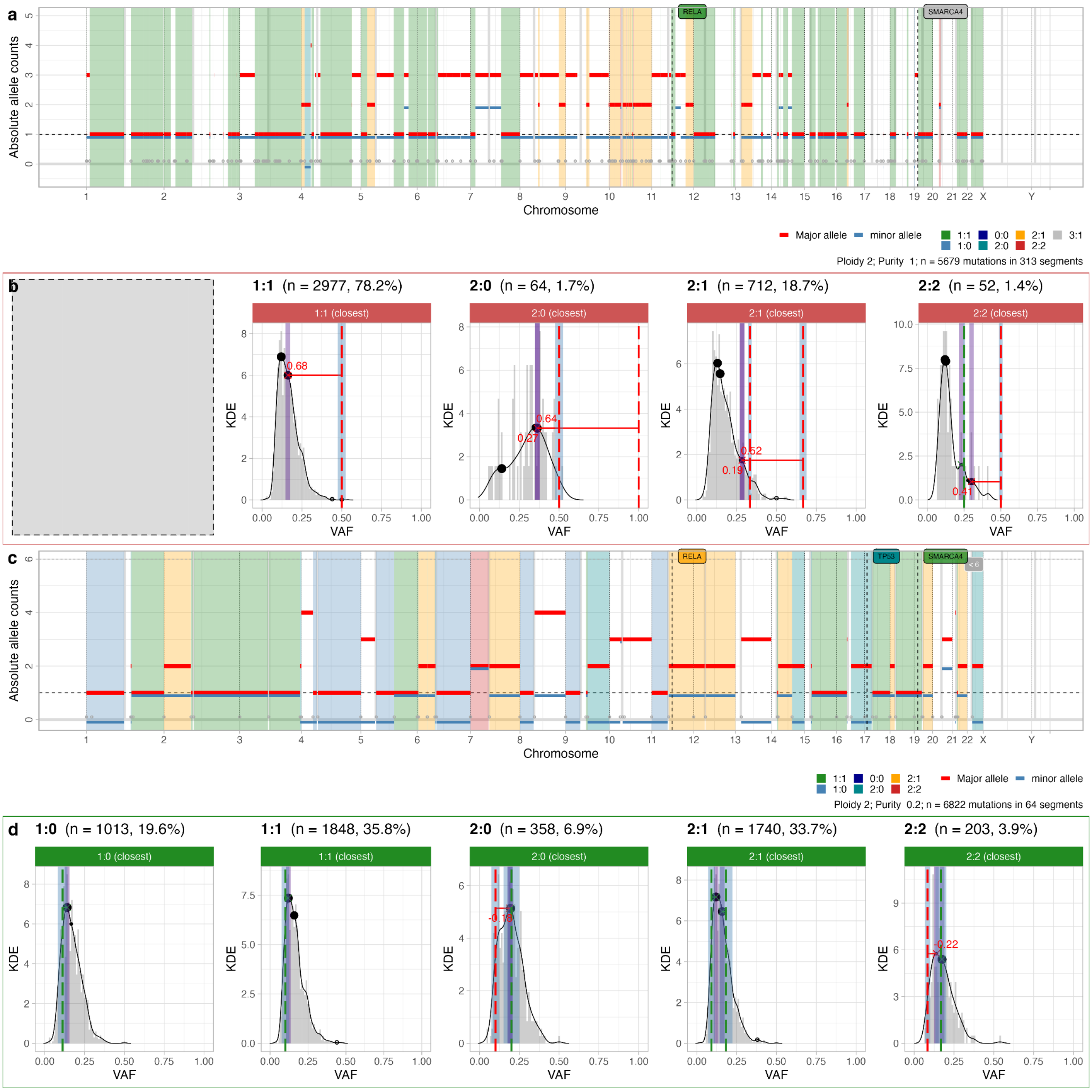

Figure S19. PCAWG high purity sample 1/2. Example PCAWG sample with 100% purity, against an alternative solution with 20% purity. **a.** The PCAWG copy number profile at purity 100% is diploid on average, with subclonal segments on chromosomes 2 and 17 (not used by CNAqc). **b.** Peak analysis with CNAqc for the PCAWG solution in panel (a). For both diploid and non-diploid regions, all peaks are mismatched (FAIL). Note that this solution has around 80% of its SNVs in diploid tumour regions, where the VAF peaks at ~10%, possibly suggesting a purity well below 100%. **c.** Rerun of this sample with the Sequenza-CNAqc pipeline (Supplementary Figure S17) assigns a diploid profile with purity 20%, obtained from one of the alternative solutions of Sequenza. **d.** VAF peaks are matched for both diploid and non-diploid regions, resulting in PASS status for each karyotype, and for the overall quality control procedure.

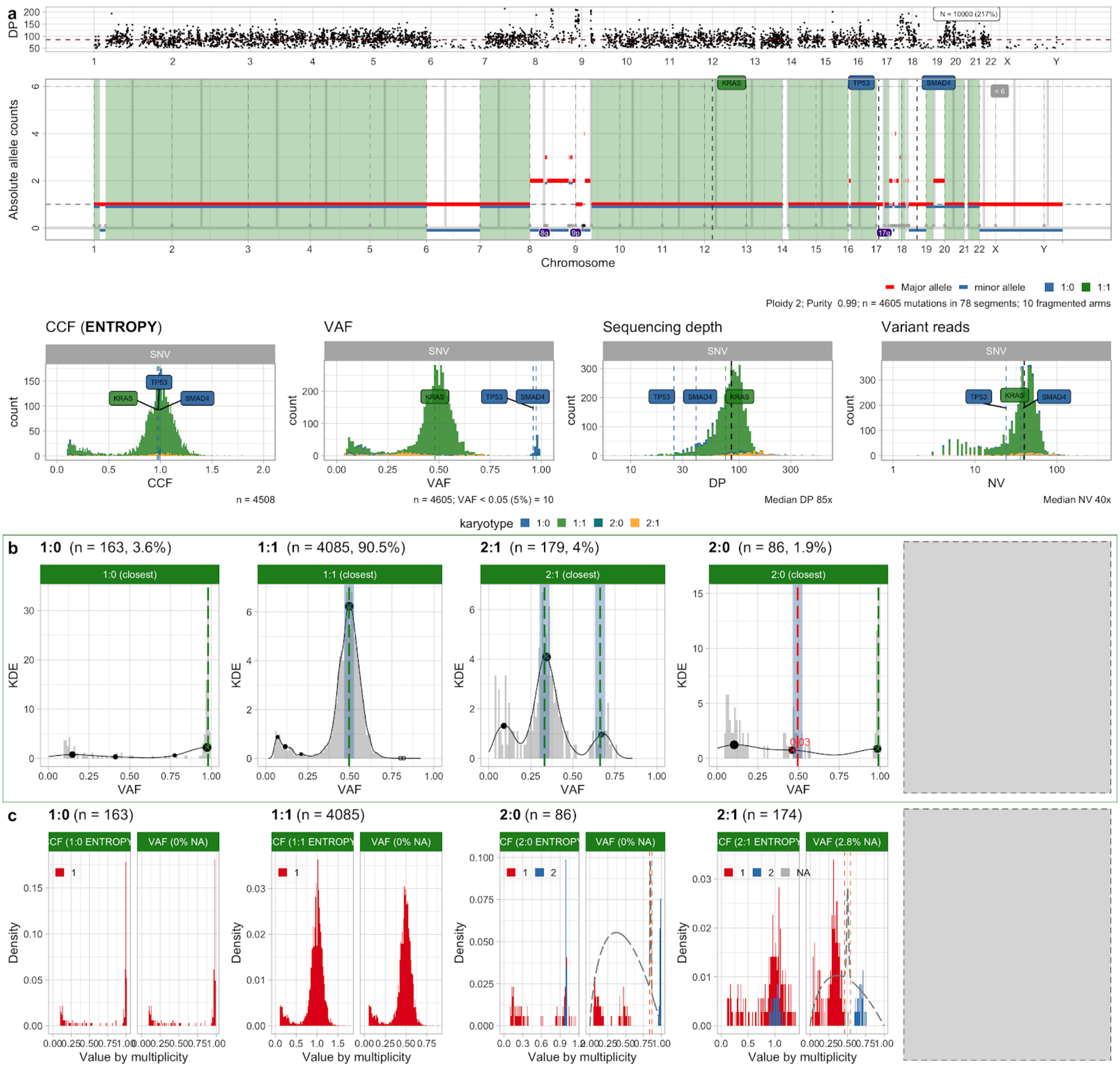

Figure S20. PCAWG high purity sample 2/2. Example PCAWG pancreatic adenocarcinoma with 99% purity (and 3 possible driver SNVs, 2 of them involving tumour suppressor genes in LOH regions). **a.** Data for the sample (genome-wide CNA segments, CCF and read counts distribution). **b.** This sample has 90% of its SNVs in diploid tumour regions, and the others in a variety of distinct CNA segments. From a peak analysis point of view, all the calls are validated. **c.** CCF values for this sample are also good.

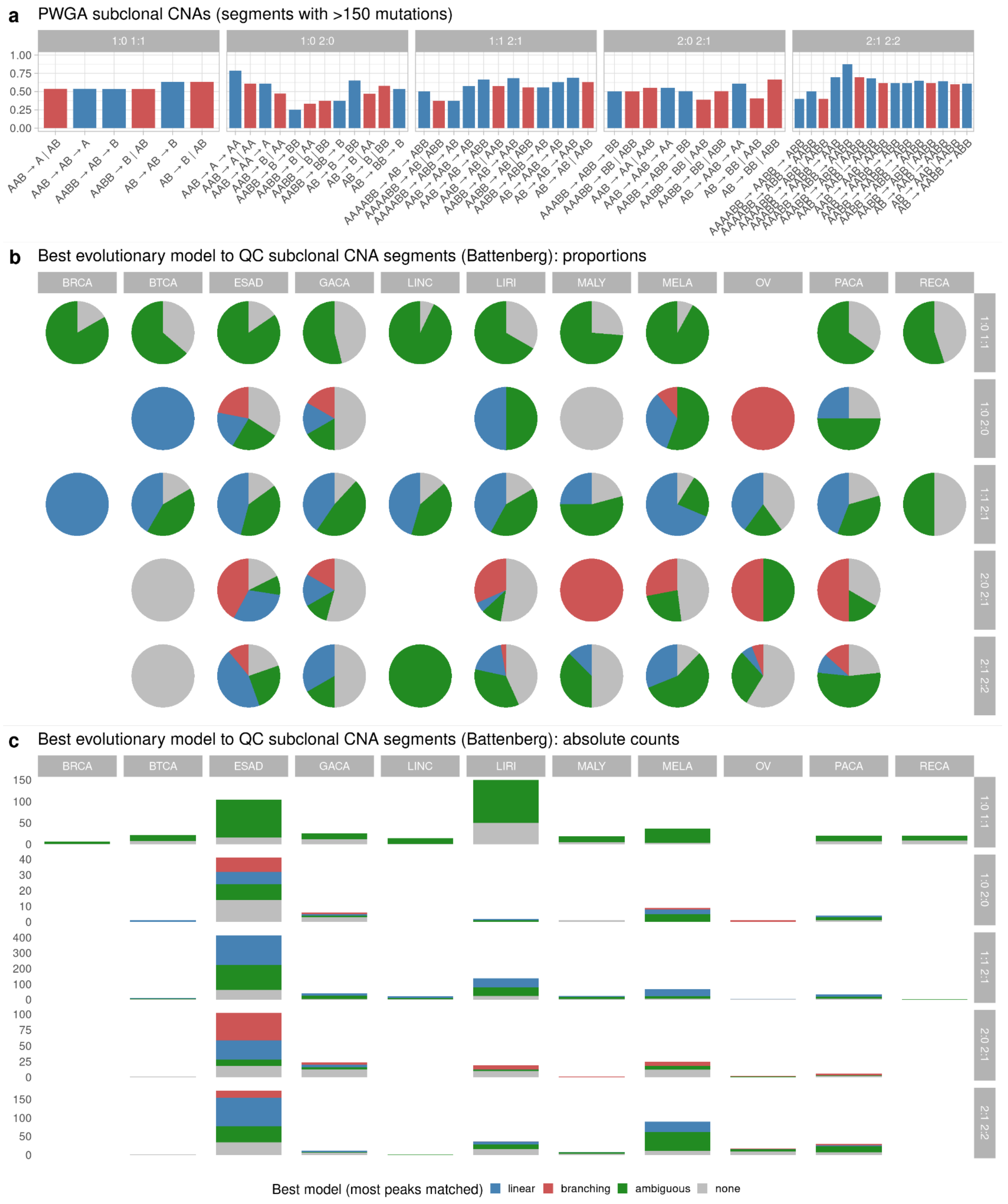

Figure S21. PCAWG evolution model for subclonal CNAs. a. Percentage of matched peaks for each possible model computed for different karyotype combinations. The colour of the bar reflects whether the referred model is branching or linear (red and blue respectively). **b.** Different prevalences of best models across different tumour types and karyotype combinations. Best models are determined according to the percentage of matched peaks. When the number of matched peaks is the same for models belonging to different classes the imputation is uncertain (ambiguous), those cases are coloured in green in the graph. Finally, when no model could be fitted to the data (<50% of the expected peaks matched), the 'none' value is assigned, those cases are coloured in grey. **c.** Absolute counts akin to the pie charts in panel (b).

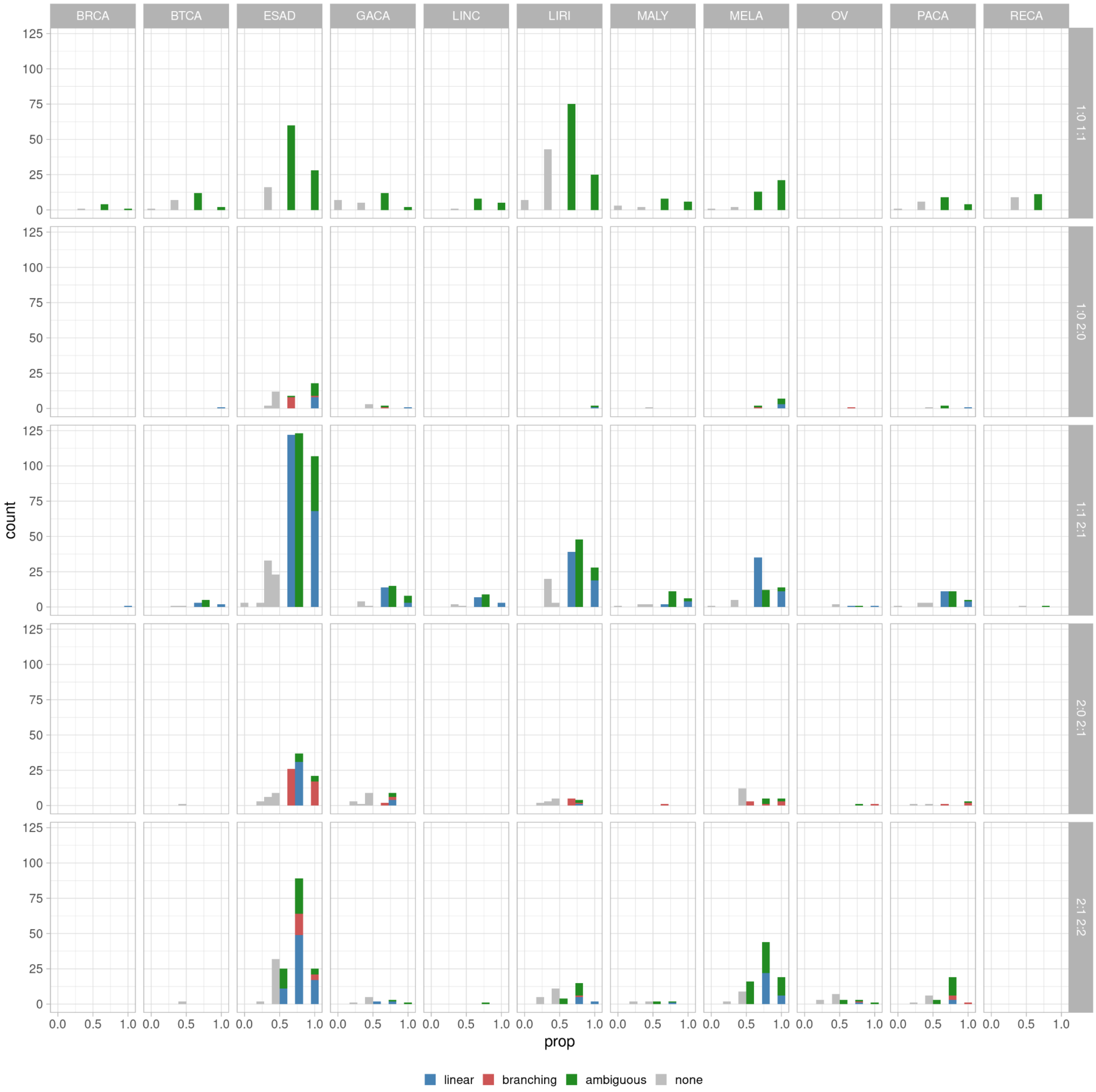

Figure S22. PCAWG evolution model for subclonal CNAs: overall counts. Number of models assigned to segments and peak matching percentages. Counts are divided according to tumour type and subclonal karyotypes. Linear models explain the data better in most cases with the exception of 2:0-2:1 karyotype where the branching model prevails, and the 1:0-1:1 karyotype where it is impossible to distinguish between models. Interestingly, similar patterns are repeated across tumours, both in terms of relative abundance of the different models and of different karyotypes. This property may suggest that the amplification mechanisms are shared across tumours, although some tumour types display a higher tendency to acquire CNAs.

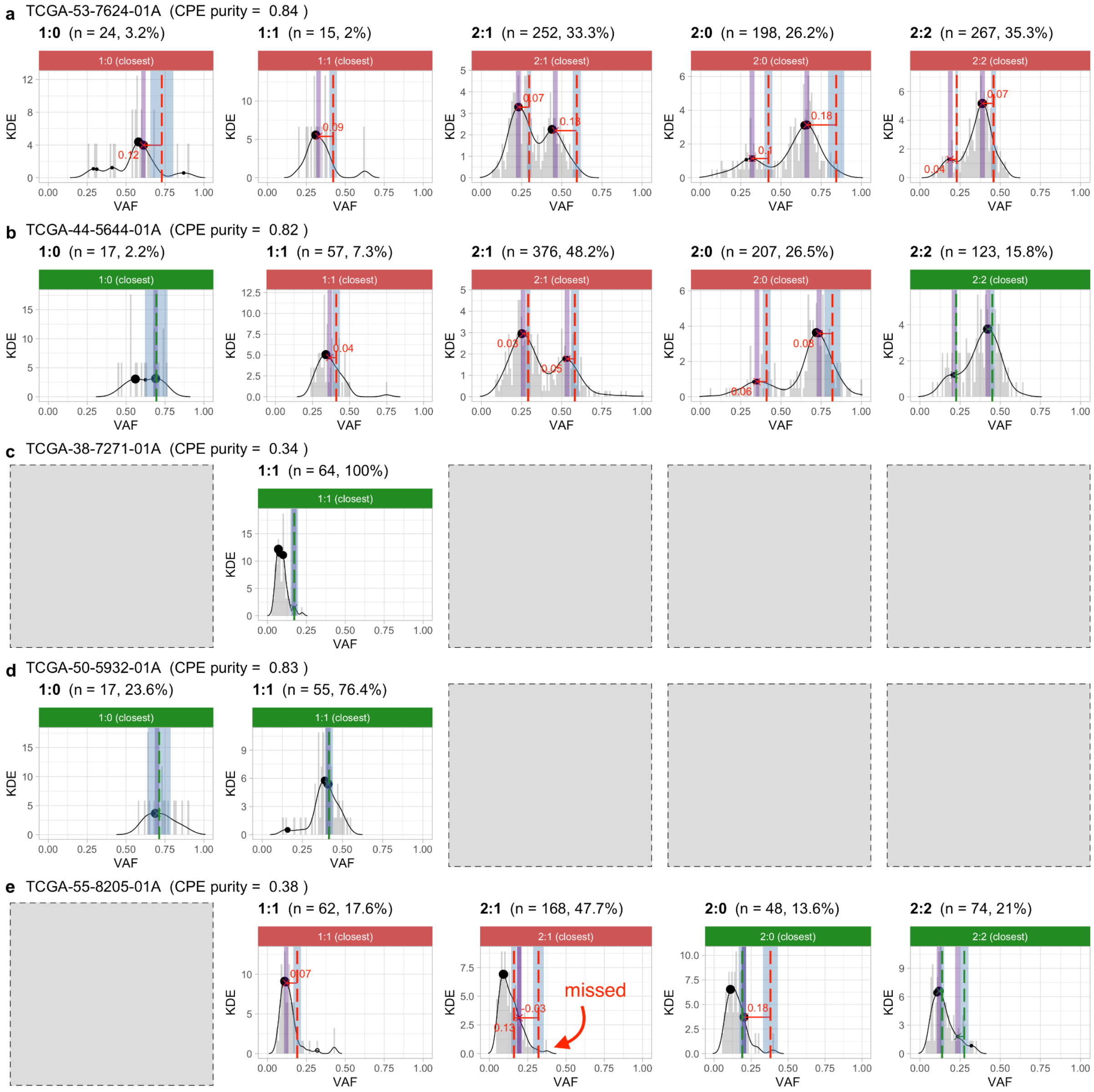

Figure S23. Example TCGA cases. a-e. CNAqc quality control via peak detection on TCGA whole-exome sequencing data of 5 lung adenocarcinomas (LUAD) with different purity values, selected from a cohort of 48 cases available online.

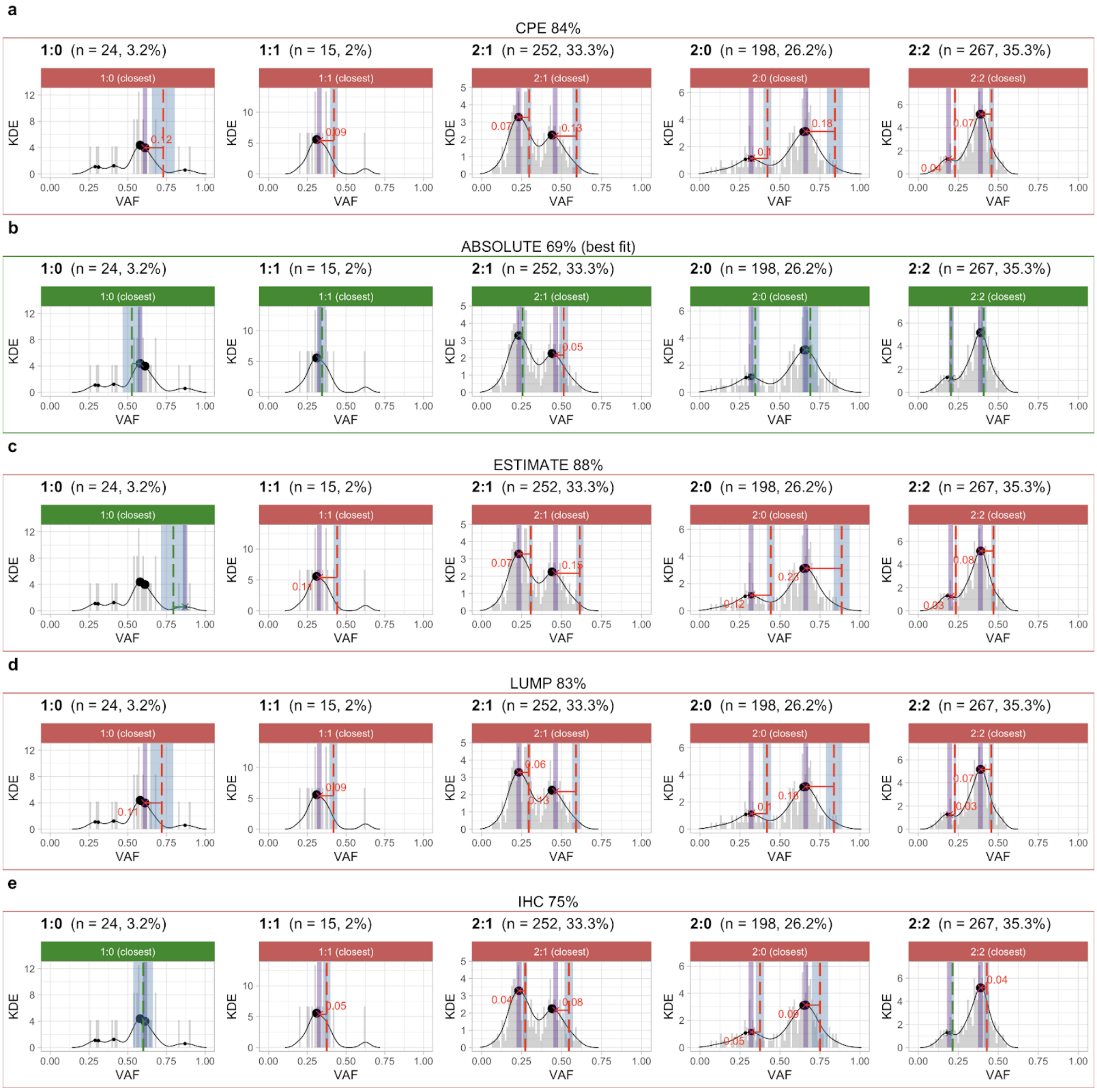

Figure S24. Example TCGA cases: multiple purity comparison. a-e. CNAqc quality control via peak detection for LUAD sample TCGA-53-7624-01A - panel (a) of Supplementary Figure S25 - using purity estimates from CPE (consensus), ABSOLUTE, ESTIMATE, IHC and LUMP. CNAqc determines that, among all callers, only ABSOLUTE detected the true tumour ploidy (69%).

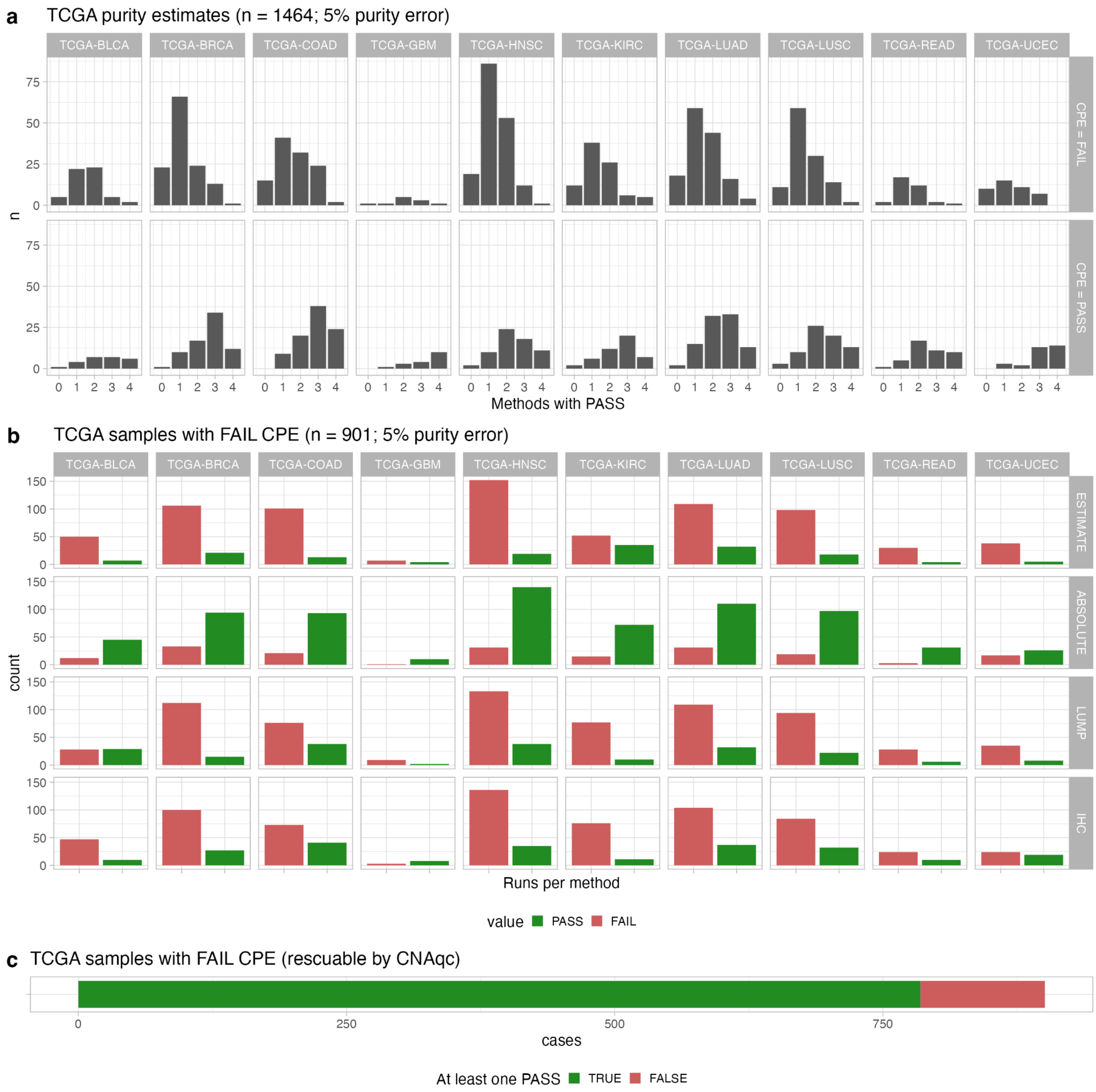

Figure S25. Overall TCGA analysis statistics. a. CNAqc quality control via peak detection for 1464 TCGA samples from 10 distinct tumour types with suitable data for our tool. Plots are split by QC status (maximum tolerated purity error 5%) based on TCGA consensus purity (CPE purity score). The bars report the number of methods whose purity is passed by CNAqc. We used purity estimates from ABSOLUTE, ESTIMATE, IHC and LUMP as in Supplementary Figure S26. **b.** For 901 cases where the CPE purity is failed by CNAqc, we report the number of cases where each method is instead passed. Note for instance that ABSOLUTE often provides a purity estimate that would pass the sample according to CNAqc, similarly to the case shown in Supplementary Figure S26. **c.** 785 out of 901 cases (~88%) would be rescued if we selected at least one of the purity estimates that passes CNAqc analysis, avoiding instead of using the CPE consensus purity which contains an error larger than 5%.

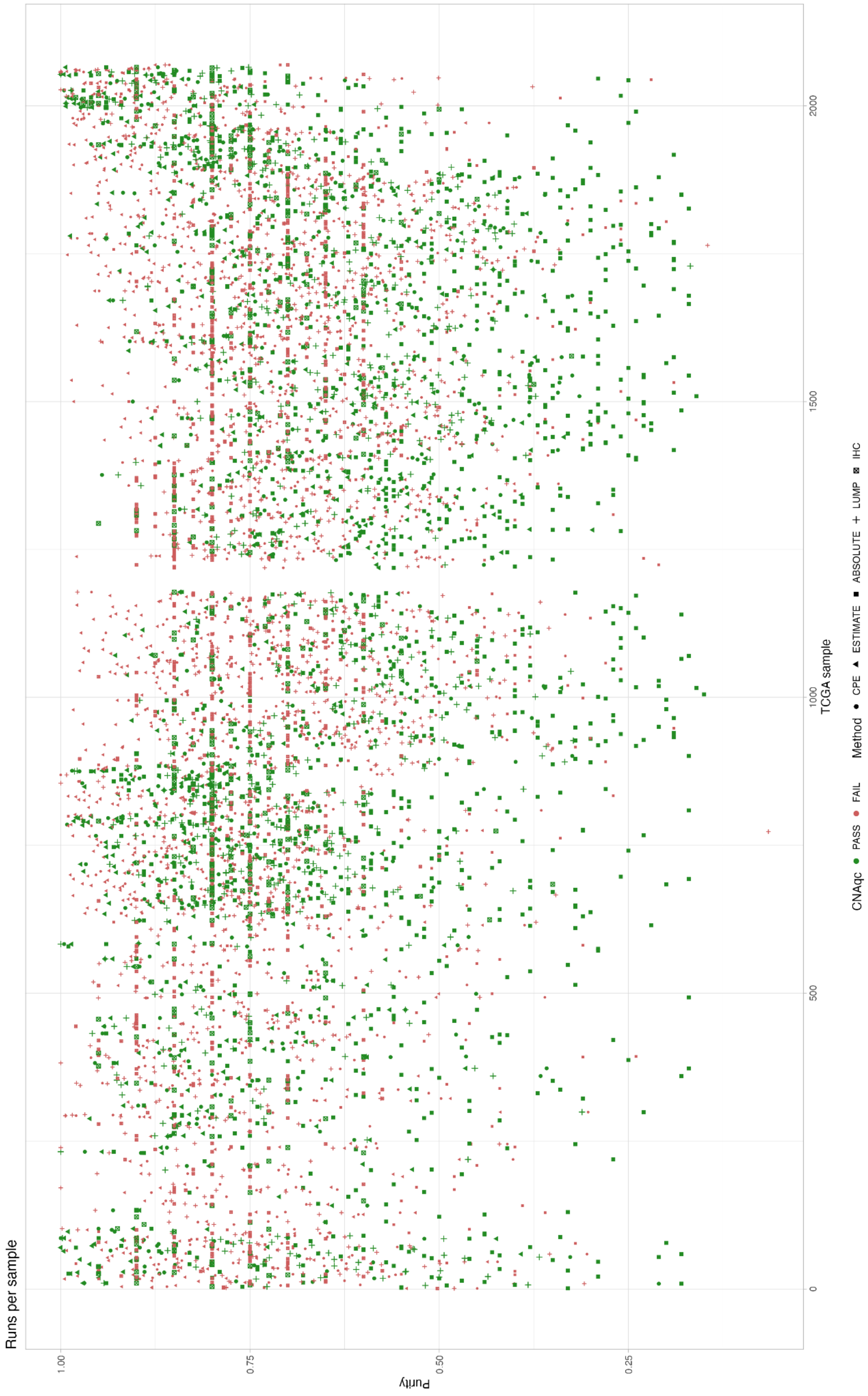

Figure S26. Overall TCGA analysis statistics: purity scatter. TCGA runs from Additional File 1: Fig. S27. On the x-axis samples are sorted by sample id; on the y-axis the purity is reported for each method. Each method has a different point shape; the colour reflects CNAqc pass or fail status. Therefore, cases where green (pass) points are at lower purity compared to red ones, represent TCGA samples where some methods over-estimate purity; in the opposite case we observe insead under-estimation.

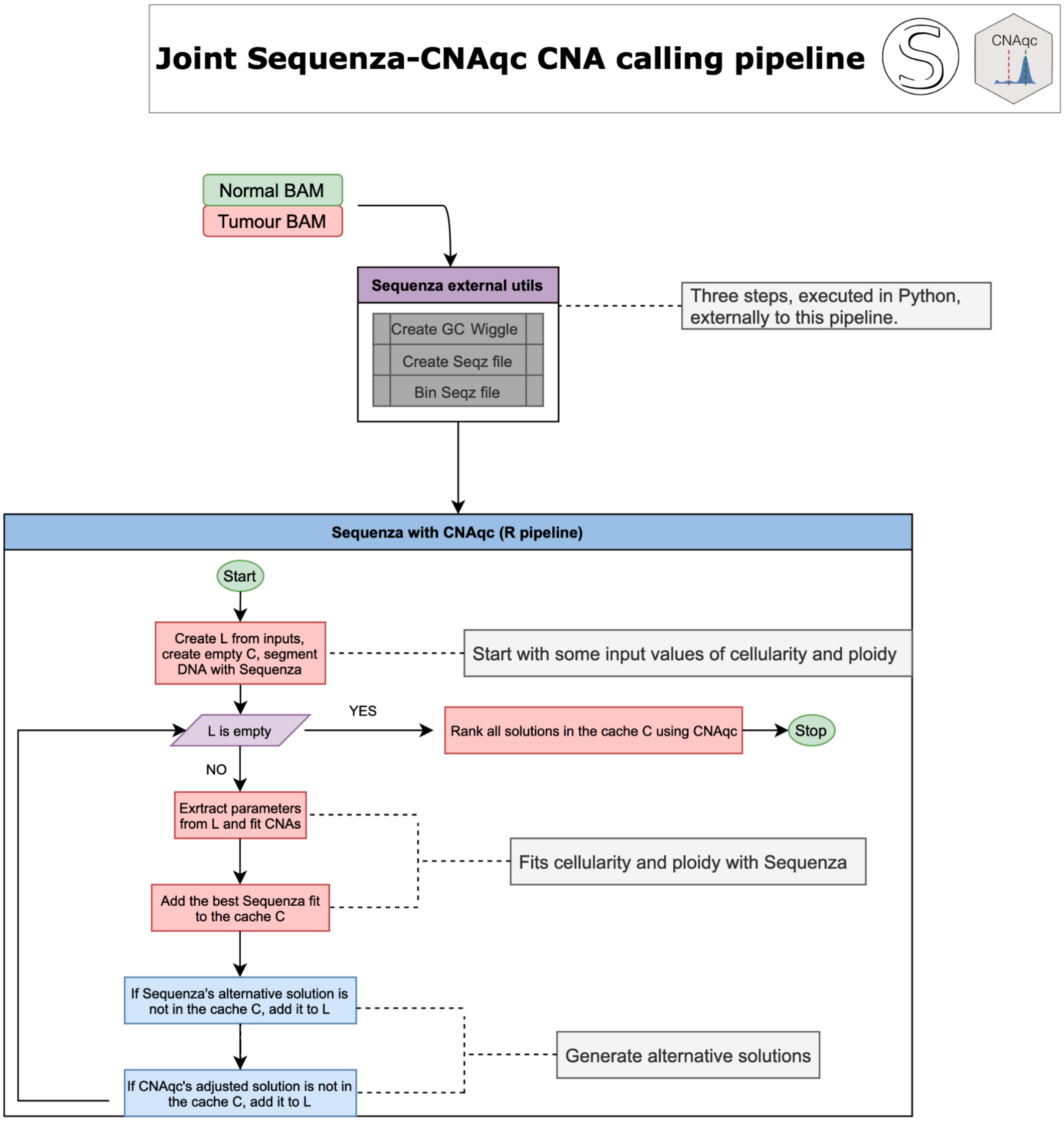

Figure S27. Sequenza-CNAqc pipeline for CNA calling. Flowchart of the joint Sequenza - CNAqc pipeline for CNA calling. Sequenza external utils are first used to generate a binned seqz file. The pipeline then performs segmentation (via Sequenza), and iteratively optimises cellularity (i.e., tumour purity) and ploidy estimation, together with allele-specific CNA segments, using a list of solutions to inspect L, and a cache of already examined solutions C. Starting from the input ranges for cellularity and ploidy, at each step Sequenza is run to fit the data. Alternative solutions are generated via Sequenza and via CNAqc: if the proposed solutions have not been already analysed (i.e., they are not in C), then they are added to the list of solutions to examine (i.e., they are instead in L). At the end, when L is empty, all the cached solutions are examined with CNAqc and ranked to determine the best fit a FAIL/PASS status by peak detection.

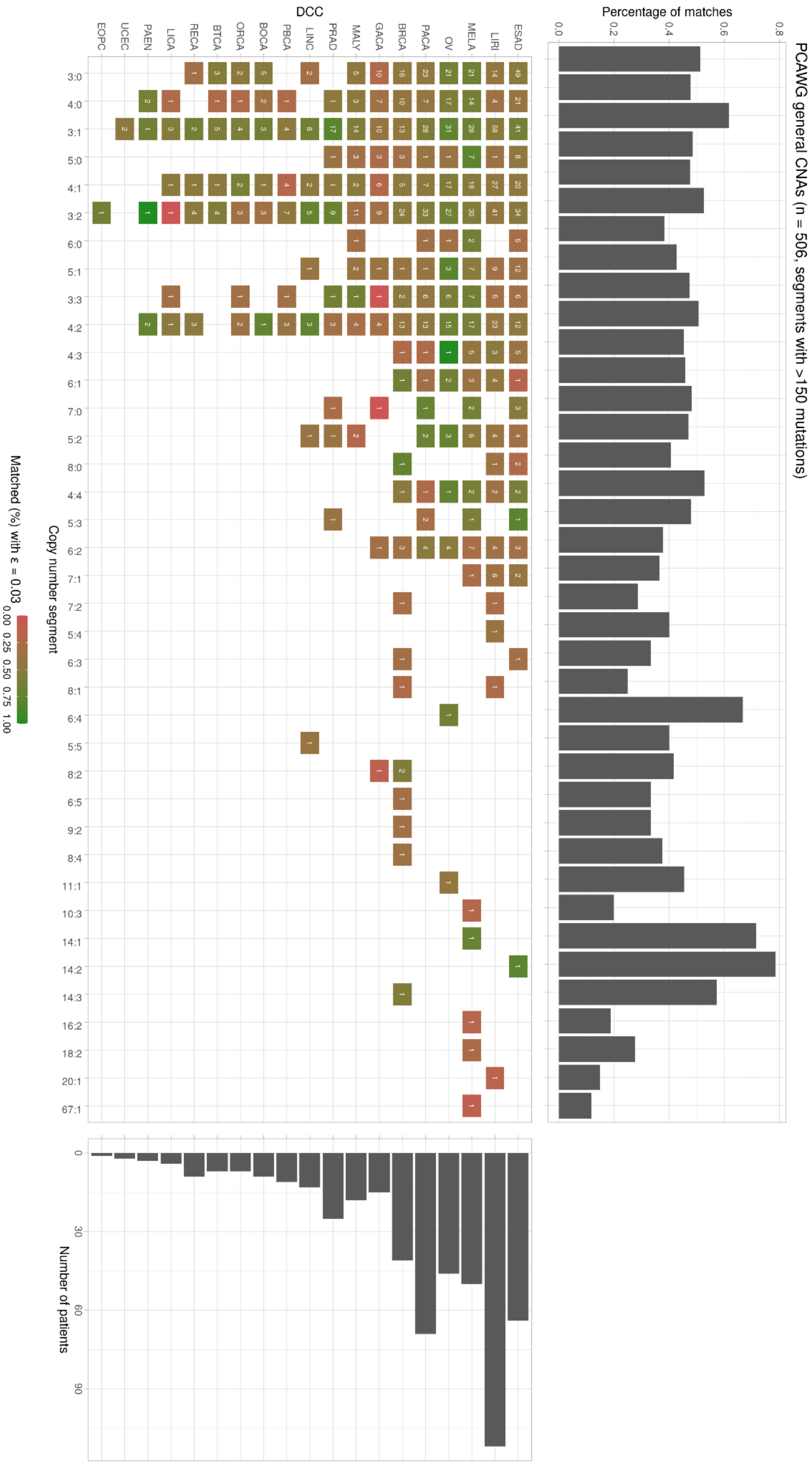

Figure S28. PCAWG samples with complex CNAs. 570 PCAWG samples with complex CNA segments. For this type of peak analysis, the tool reports if any of a set of expected peaks is detected in the data. Since segments with a low mutational burden carry little peak information and are prone to noise, we show only segments with >150 somatic mutations. The marginal right panel shows the percentage of matched peaks for each rare karyotype found in the PCAWG cohort. In the central panel, segments are divided according to their karyotype and tumour type: each square reports the number of segments and its colour encodes the percentage of matched peaks. The marginal bottom panel shows the number of patients for each tumour type.

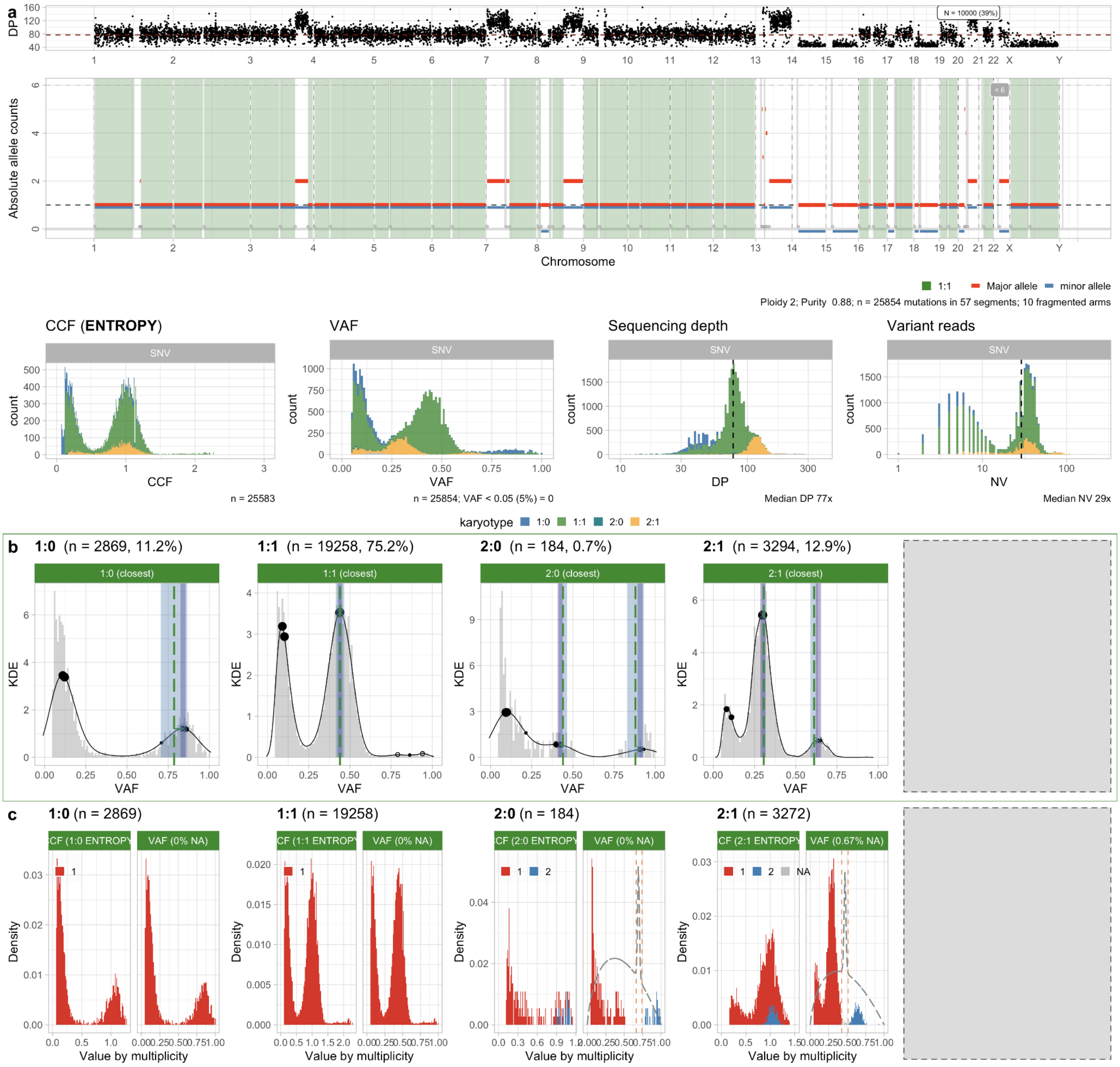

a
DP
Absolute allele counts
Chromosome
N = 10000 (39%)
1:1
Major allele
minor allele
Ploidy 2; Purity 0.88; n = 25854 mutations in 57 segments; 10 fragmented arms
CCF (ENTROPY)
VAF
Sequencing depth
Variant reads
SNV
count
CCF
VAF
DP
NV
n = 25583
n = 25854; VAF < 0.05 (5%) = 0
Median DP 77x
Median NV 29x
karyotype 1:0 1:1 2:0 2:1
b
1:0 (n = 2869, 11.2%)
1:1 (n = 19258, 75.2%)
2:0 (n = 184, 0.7%)
2:1 (n = 3294, 12.9%)
1:0 (closest)
1:1 (closest)
2:0 (closest)
2:1 (closest)
KDE
c
1:0 (n = 2869)
1:1 (n = 19258)
2:0 (n = 184)
2:1 (n = 3272)
VAF (0% NA)
VAF (0.67% NA)
Density
Value by multiplicity

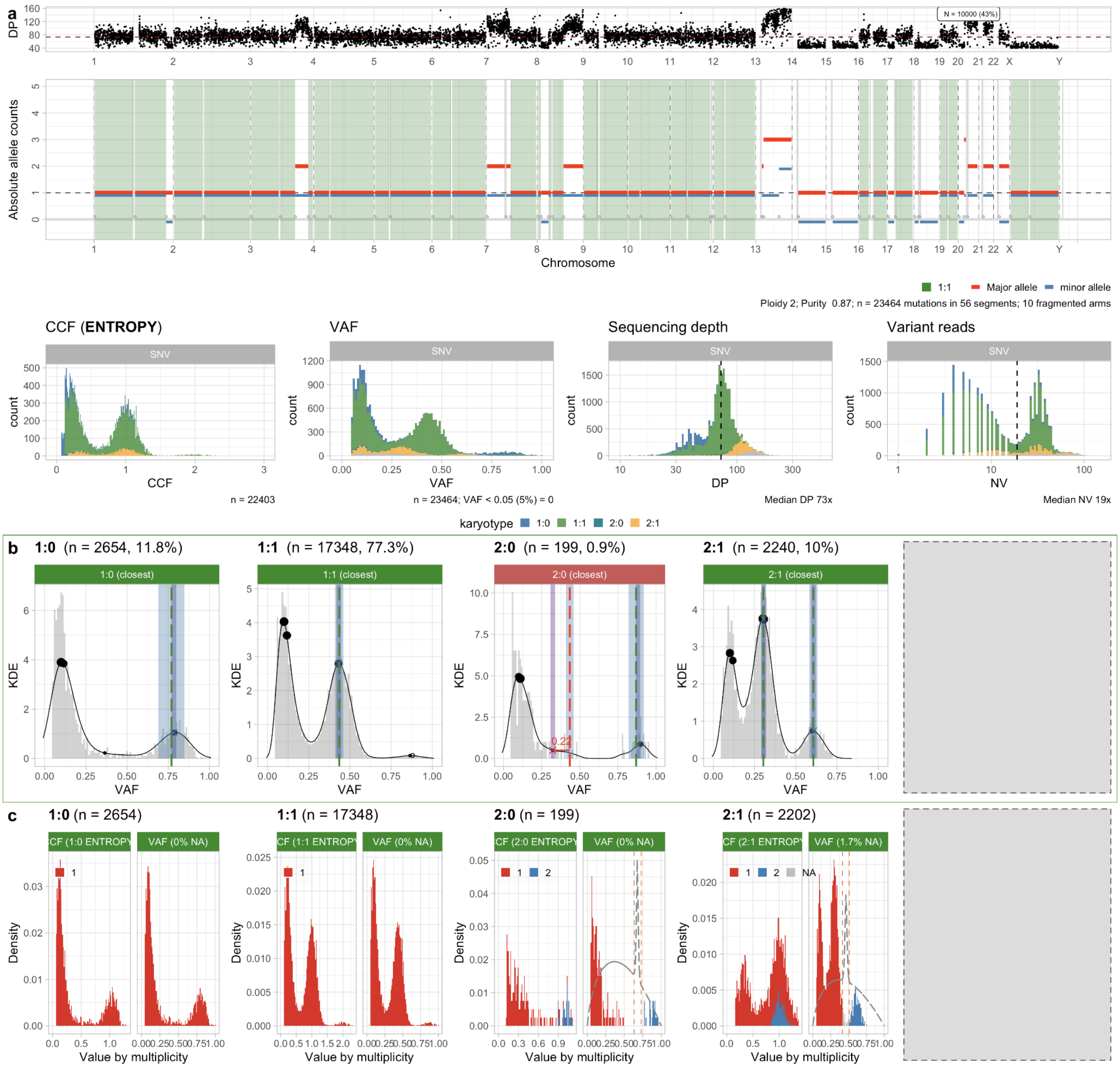
a
DP
Absolute allele counts
Chromosome
1:1 Major allele minor allele
Ploidy 2; Purity 0.87; n = 23464 mutations in 56 segments; 10 fragmented arms
CCF (ENTROPY)
VAF
Sequencing depth
Variant reads
n = 22403
n = 23464; VAF < 0.05 (5%) = 0
Median DP 73x
Median NV 19x
karyotype 1:0 1:1 2:0 2:1
b
1:0 (n = 2654, 11.8%)
1:1 (n = 17348, 77.3%)
2:0 (n = 199, 0.9%)
2:1 (n = 2240, 10%)
c
1:0 (n = 2654)
1:1 (n = 17348)
2:0 (n = 199)
2:1 (n = 2202)
Value by multiplicity

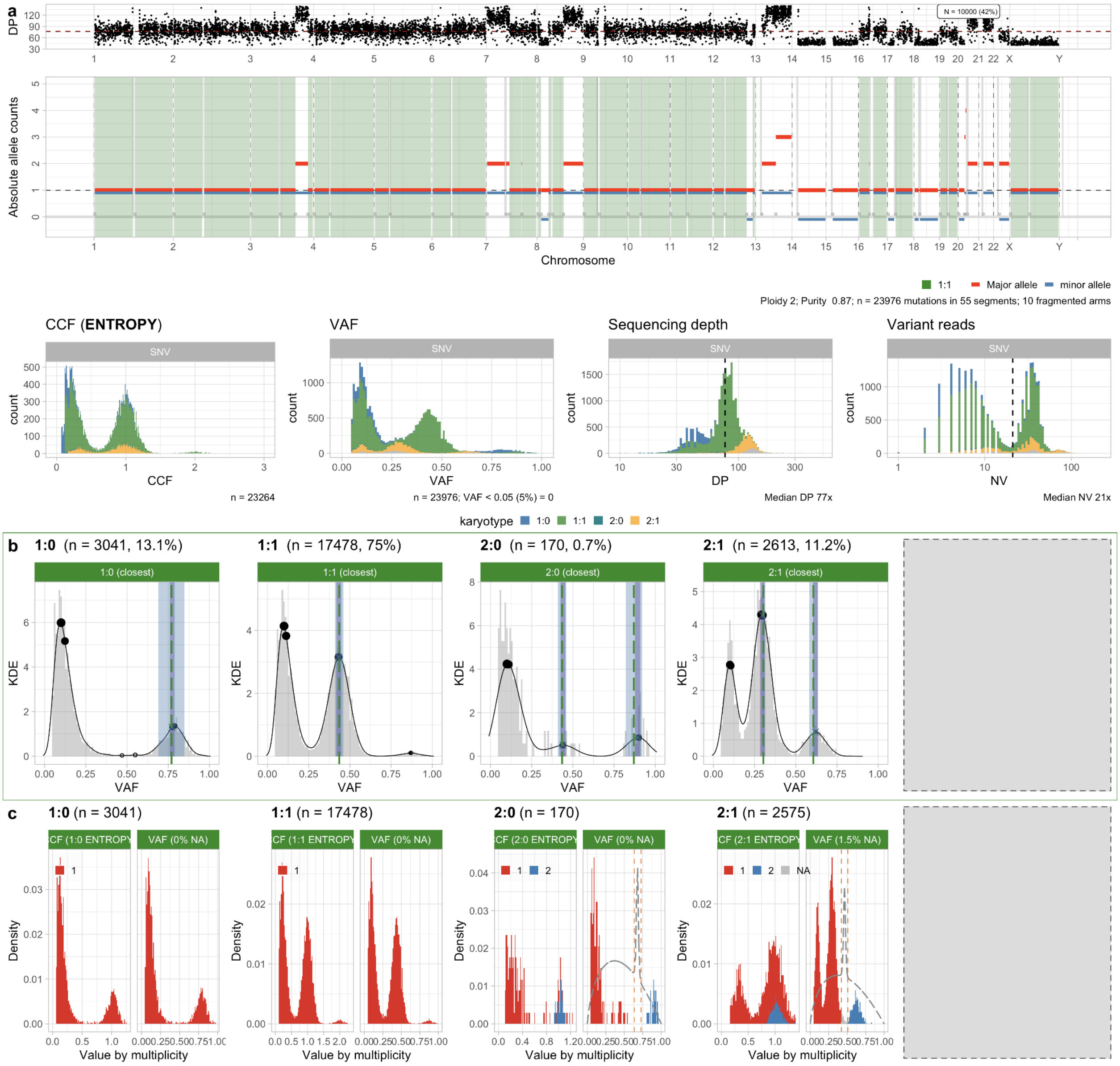
a
DP
N = 10000 (42%)
Absolute allele counts
Chromosome
1:1 Major allele minor allele
Ploidy 2; Purity 0.87; n = 23976 mutations in 55 segments; 10 fragmented arms
CCF (ENTROPY)
VAF
Sequencing depth
Variant reads
SNV
count
CCF
n = 23264
n = 23976; VAF < 0.05 (5%) = 0
DP
Median DP 77x
NV
Median NV 21x
karyotype 1:0 1:1 2:0 2:1
b
1:0 (n = 3041, 13.1%)
1:1 (n = 17478, 75%)
2:0 (n = 170, 0.7%)
2:1 (n = 2613, 11.2%)
1:0 (closest)
1:1 (closest)
2:0 (closest)
2:1 (closest)
KDE
VAF
c
1:0 (n = 3041)
1:1 (n = 17478)
2:0 (n = 170)
2:1 (n = 2575)
CCF (1:0 ENTROPY)
VAF (0% NA)
CCF (1:1 ENTROPY)
CCF (2:0 ENTROPY)
CCF (2:1 ENTROPY)
VAF (1.5% NA)
Density
Value by multiplicity

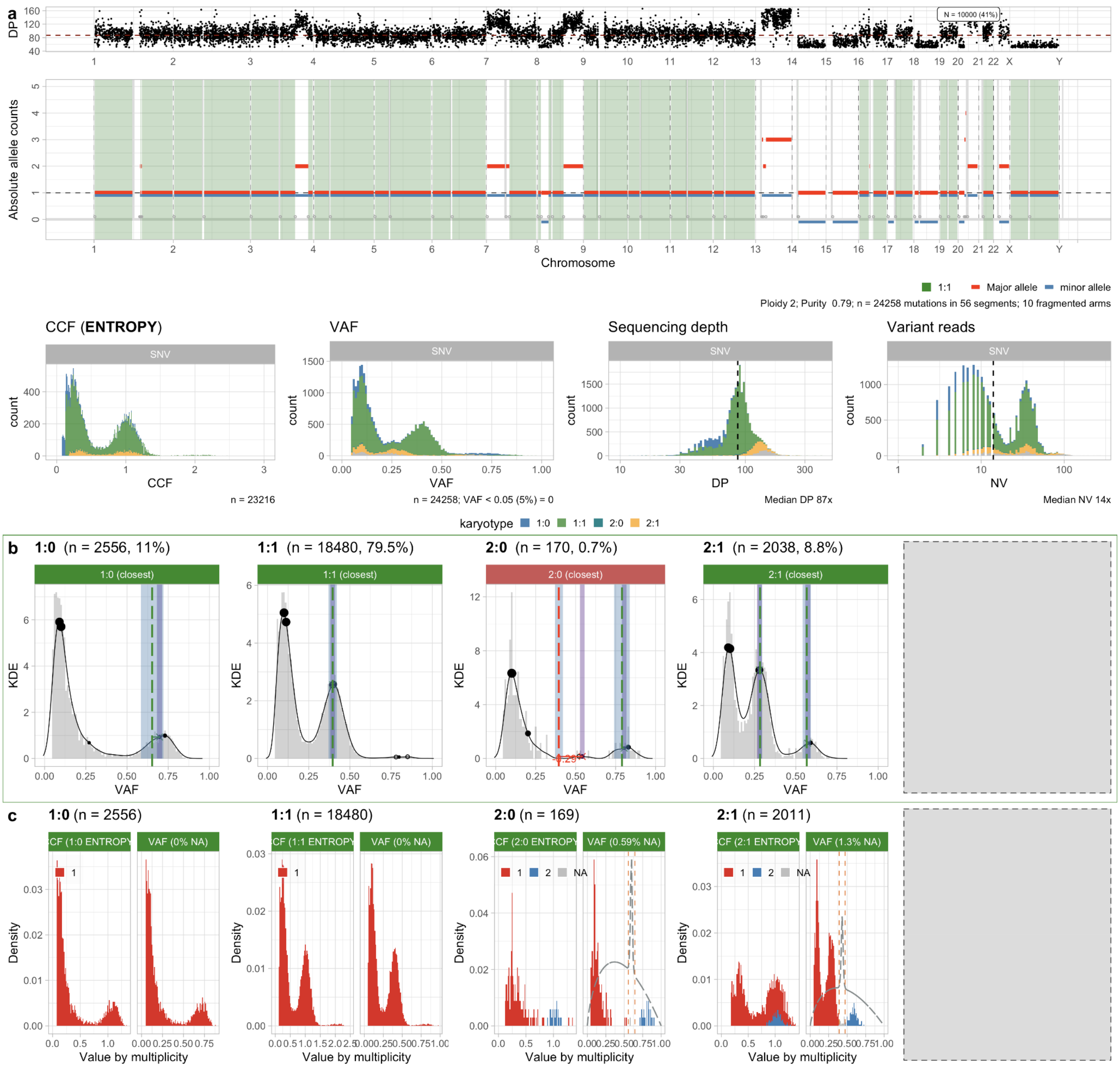

Figure S29. Colorectal multi-region data analysis (multiple pages). Colorectal multi-region samples (one per page): Set7_55, Set7_57, Set7_59 and Set7_62 for patient Set7. **a.** Allele-specific CNAs, and read data distribution (bottom row). **b,c.** Peak analysis and CCF computation for the sample.

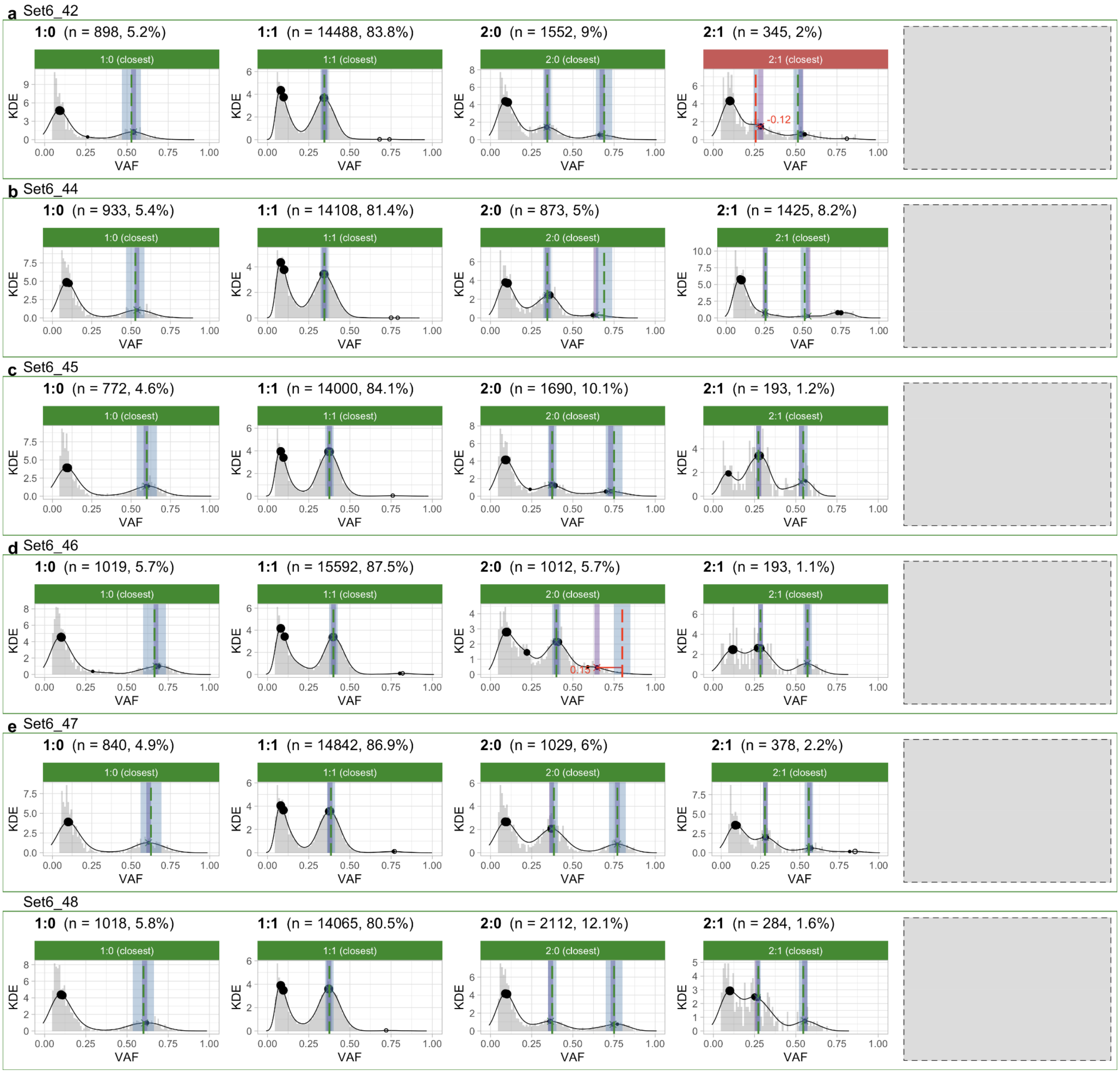

Figure S30. Colorectal multi-region data analysis: peaks. a,b,c,d,e. Peak detection quality control with CNAqc, run with default parameters on colorectal multi-region samples available for patient Set_6. All calls are passed (surrounding green rectangles).
