# Supplementary material for: Computational validation of clonal and subclonal copy number alterations from bulk tumor sequencing using CNAqc
Source: Genome Biol. 2024 Jan 31;25:38. doi: 10.1186/s13059-024-03170-5 (PMC10832148; doi:10.1186/s13059-024-03170-5)
Supplement: Supplementary file 2 — Additional file 2. Review history. [file 13059_2024_3170_MOESM2_ESM.docx]

Review History

**First round of review**

**Reviewer 1**

**Were you able to assess all statistics in the manuscript, including the appropriateness of statistical tests used?** Yes: Appropriate.

**Were you able to directly test the methods?** No.

**Comments to author:**

This paper introduces CNAqc, a method to verify CNA calls of bulk DNA sequencing data via orthogonal SNV measurements. The idea is novel, the experimental evaluation is thorough and the paper is well written. This work will be of interest to the readership of this journal. I have two broader, discussion-related comments and several minor comments.

Major comment

1. Support for multi-sample data

Presently, the manuscript assume a single sample per tumor. I would like to see a discussion on the case of multiple samples per tumor (e.g. multi-region data). While the evolutionary history will be shared, there will be sample-specific purity and clone proportion values. Moreover, there might be more than two non-normal CNA states (when looking across samples). It would be good to comment on this.

2. How to validate methods that infer clone-specific copy-number profiles?

CNAqc considers the clonal (and additionally subclonal) of each segment in isolation. This is in line with the output of many current CNA callers such as Battenberg that produce CNA calls of segment, without attempting to infer genome-wide clone-level CNA profiles. However, there are CNA callers, such as HATCHet, that infer clone-specific copy-number profiles, where each clone has a prevalence in the sample. Is there an opportunity to assess goodness of fit of clone-level proportions for this kind of data?

Minor comments

- Page 7, line 36: It looks like you are assuming that n_A >= n_B. It would be good to explicitly state this.

- Page 30, line 13: 0 < c < 1 should be 0 < c <= 1.

- Page 31, line 36: arg_i min => arg min_i (and min should be non-italic).

- Page 32, line 28: replace semicolon with comma in first interval.

- Page 36, paragraph starting at line 12: restate which purity and clone proportion values are considered for the example.

- Page 36, line 44: there is an unmatched '[' -- also should there be parentheses around (m * pi) in the denominator?

- Page 37, line 11: typo: algorithsm

**Reviewer 2**

**Were you able to assess all statistics in the manuscript, including the appropriateness of statistical tests used?** Yes: none.

**Were you able to directly test the methods?** No.

**Comments to author:**

The authors propose a new method for performing quality control of allele specific copy number calls from bulk genome sequencing data of cancers. The core challenge in this field is selecting between numerous equally plausible ploidy and tumour content solutions, as these two variables are confounded in most models of copy number analysis. The key innovation the authors propose is to use single nucleotide variants (SNVs) as an orthogonal feature to score different proposed solutions. The authors primarily focus on the analysis of "simple" copy number regions i.e. those which would most parsimoniously be explained by a single evolutionary event. They derive an equation which links the expected peaks in the variant allele frequency distribution that one would expect to observe given clonal (cancer cell fraction of 1.0) mutations in these regions. By applying peak detection algorithms and comparing the inferred peaks to theoretical values they obtain a method for identifying ploidy/purity solutions compatible with the SNVs. The authors further explore the use of their tool in "complex" CNV regions, to determine cancer cell fraction and to identify branched vs linear evolution. The method is tested using a large set of synthetic tumours as well as in-silico mixtures of single cell whole genome sequencing data. The utility of the method is demonstrated by analysing three large cancer sequencing datasets.

Overall, the method is well thought out and solves an important problem when inferring copy number from bulk sequencing data. The approach is not completely novel, as the core equation that powers the approach is well established in both the copy number inference (using SNPs in place of SNVs) and the clonal analysis literature. However, the use of SNVs to select between solutions for copy number callers is novel, at least as far as published tools are concerned. Adhoc versions of the procedure have likely been used in various studies over the years. ABSOLUTE also used very similar principles to estimate per mutation CCFs. Those caveats about novelty aside, I am not aware of any easily runnable tool that performs the task of copy number quality control proposed in this work. As such, I see it as a useful contribution.

The tool has been reasonably tested using a combination of synthetic and real single cell data. Application to real world datasets was extensive, with some compelling examples shown. I do have some concerns about the selection of ABSOLUTE as the best inference procedure in a few examples which I will discuss below. Overall though, the testing was well done.

My primary concern with the manuscript in the current form is that the methods are difficult to follow. There are a few factors at play. First, notation is not established and consistently used. Second, several sections interleave interpretation of results into the methods making it difficult to follow the exact design choices. Third, there are several typos and overloaded notations which are challenging. I provide a few examples in my detailed notes below.

Detailed notes:

-There were no page numbers so pointing to specific sections is tricky.

- I am somewhat concerned that there is circularity in selecting ABSOLUTE, as it also performs similar CCF and peak finding for SNVs. There have been several updates to the software since it was published and it is unclear whether SNVs contribute to the selected CNV solutions. It would be helpful for the authors to clarify this.

- I am unclear on the use case for computing CCFs with this tool. The authors suggest that it could be helpful to QC SNVs which are poor fits. But these could just be from sampling noise, which is already modelled by tools such as PyClone-VI.

- Taxonomy section. Are sex chromosomes considered? Presumably the definition of simple and complex will differ for males.

- Simple CNA section. It is suggested whole genome duplications can be detected with SNVs. I see that this would induce extra peaks, but it might be worth providing an example for the reader.

- Simple CNA section. The equation 1 <= m <= n_b seems superfluous given the constraint 1 <= m <= n_a and by definition n_a >= n_b.

- Simple CNA section. $v_2(2)=0.5$ does not make sense as it implies a CCF of 2.

- Simple CNA section. Indels are mentioned as being compatible with the method. However, the VAF of indels is usually noisier due to alignment bias. Can the authors comment?

- Conversion among VAF … section. The notation is heavily overloaded here. For example $v_m(\pi)$ suggests purity is being used where previously CCF was the argument. There are also several epsilons, perhaps different well defined symbols would be clearer.

- Sample level score section. Why are the sample sample metrics allowed to be positive or negative. Is there not a risk of different values cancelling, leading to erroneously low values. I would have thought an absolute value in equation 1.5 would make more sense.

- Same section. The subscript on the sum of $\lambda_k^FAIL$ is $P_k$ and should be $F_k$ I believe. This affects equation 1.6.

- I had some confusion when understanding CCF of clones. In some cases it refers to the SNVs and in others the CNV segments. A clear notation to distinguish the two and clear delineation in the text would help.

- I have some concerns about peak detection. In cases of closely spaced peaks, stochastic sampling could provide the elusion of a single peak, albeit with a longer tail. This is a common feature in mixture models. Is the method robust to this or will it miss these peaks?

- Figure 5. A capital D is used in the caption which does not match the rest of the panel labels.

- Supp Figure 23. Panel e is not labelled or mentioned in the caption.

**Authors Response**

**Point-by-point responses to the reviewers’ comments:**

**Reviewer #1**

This paper introduces CNAqc, a method to verify CNA calls of bulk DNA sequencing data via orthogonal SNV measurements. The idea is novel, the experimental evaluation is thorough and the paper is well written. This work will be of interest to the readership of this journal. I have two broader, discussion-related comments and several minor comments.

We thank the reviewer for the positive assessment of our work, and for the notion that it would be of interest to the readership of Genome Biology.

**Major comment**

1. Support for multi-sample data

Presently, the manuscript assumes a single sample per tumour. I would like to see a discussion on the case of multiple samples per tumour (e.g. multi-region data). While the evolutionary history will be shared, there will be sample-specific purity and clone proportion values. Moreover, there might be more than two non-normal CNA states (when looking across samples). It would be good to comment on this.

The reviewer is correct to observe that, at the moment, CNAqc cannot jointly analyse multiple samples and infer a unique evolutionary history for subclonal CNAs. In the paper, we apply CNAqc to multi-region data as discussed in Section *Multi-region colorectal cancer WGS* (Online methods), with results presented in Supplementary Figures S29 and S30. However, in that case study, we focused on clonal segments, consistently with the availability of ground truth data.

As the reviewer points out, the evolutionary inference from multi-region data would require accounting for multiple possible scenarios, depending on the clonality (clonal vs subclonal) of each tumour clone across samples. We do believe that dedicated algorithms that are not yet available in our tool would be required to approach this problem in a systematic way, and a future extension of CNAqc could focus on this and other aspects.

We now mentioned this in the Discussion and in the Section *“Multi-region colorectal cancer WGS”.*

2. How to validate methods that infer clone-specific copy-number profiles?

CNAqc considers the clonal (and additionally subclonal) of each segment in isolation. This is in line with the output of many current CNA callers such as Battenberg that produce CNA calls of segment, without attempting to infer genome-wide clone-level CNA profiles. However, there are CNA callers, such as HATCHet, that infer clone-specific copy-number profiles, where each clone has a prevalence in the sample. Is there an opportunity to assess goodness of fit of clone-level proportions for this kind of data?

It is theoretically possible, provided that the subclonal segments of interest have only two CNA states, to validate the results of HATCHet by comparing the concordance of the VAF spectrum with the calls for each segment made by HATCHet. In fact, we have tested this in a parallel project (reported below) on unpublished data generated in our laboratory.

In this context, the segment-wise analysis implemented in CNAqc was useful to double-check HATCHet results, which were sometimes minimally inaccurate (reported below). In general, the CNAqc framework is quite flexible and extensible, and one for instance could derive a clone-level goodness-of-fit by looking at each subclonal segment of the target clone and verify that they all pass the QC control (e.g., a majority voting). However, for the moment, there is no automatic procedure to do it, but we plan to investigate this at a  later stage (possibly in collaboration with the Raphael group that has developed HATCHet).

To keep the manuscript focused, we report example analyses for the reviewer just below, using samples from a private cohort of colorectal organoids we are developing.

***a.*** *BAF and DR segmentation results from HATCHet, on data from an independent project where we process patient-derived organoids. Segments are coloured according to the cluster they belong to in BAF/DR space.* ***b.*** *HATCHet finds* *clone-specific copy number profiles and subclone proportions by clustering SNPs in BAF/DR space. Each cluster corresponds to a different copy number state. On the centroid is reported the allele specific copy number state enclosed in brackets: when a single bracket is reported the state is clonal, when two brackets are present, the state is subclonal.* ***c.*** *Segment-specific quality control performed by CNAqc for subclonal segments: on the left are shown three segments for which HATCHet predictions are in good agreement with VAF spectrum peaks expectations, on the right, instead, HATCHet predictions with less support from CNAqc.*

**Minor comments**

- Page 7, line 36: It looks like you are assuming that n_A >= n_B. It would be good to explicitly state this.

Done

- Page 30, line 13: 0 < c < 1 should be 0 < c <= 1.

Done

- Page 31, line 36: arg_i min => arg min_i (and min should be non-italic).

Done

- Page 32, line 28: replace semicolon with comma in first interval.

Done

- Page 36, paragraph starting at line 12: restate which purity and clone proportion values are considered for the example.

Done

- Page 36, line 44: there is an unmatched '[' -- also should there be parentheses around (m * pi) in the denominator?

Done

- Page 37, line 11: typo: algorithsm

Done

**Reviewer #2**

The authors propose a new method for performing quality control of allele specific copy number calls from bulk genome sequencing data of cancers. The core challenge in this field is selecting between numerous equally plausible ploidy and tumour content solutions, as these two variables are confounded in most models of copy number analysis. The key innovation the authors propose is to use single nucleotide variants (SNVs) as an orthogonal feature to score different proposed solutions. The authors primarily focus on the analysis of "simple" copy number regions i.e. those which would most parsimoniously be explained by a single evolutionary event. They derive an equation which links the expected peaks in the variant allele frequency distribution that one would expect to observe given clonal (cancer cell fraction of 1.0) mutations in these regions. By applying peak detection algorithms and comparing the inferred peaks to theoretical values they obtain a method for identifying ploidy/purity solutions compatible with the SNVs. The authors further explore the use of their tool in "complex" CNV regions, to determine cancer cell fraction and to identify branched vs linear evolution. The method is tested using a large set of synthetic tumours as well as in-silico mixtures of single cell whole genome sequencing data. The utility of the method is demonstrated by analysing three large cancer sequencing datasets.

Overall, the method is well thought out and solves an important problem when inferring copy number from bulk sequencing data. The approach is not completely novel, as the core equation that powers the approach is well established in both the copy number inference (using SNPs in place of SNVs) and the clonal analysis literature. However, the use of SNVs to select between solutions for copy number callers is novel, at least as far as published tools are concerned.

We thank the reviewer for the accurate summary of our contribution, and for noting the novelty and importance of the problem we try to approach. It is true that the combinatorial framework used to defined expected VAFs in CNAqc is introduced, with SNPs instead of SNVs, in the ASCAT paper, as we explicitly refer in Section “Simple clonal CNAs” (sub-section “Expected clonal VAF peaks”, second paragraph) and in the bibliography (reference 21). However, we agree with the reviewer that it is the first time that SNVs are used to define QC metrics for clonal and subclonal copy number detection, which is the main novelty of this work.

Adhoc versions of the procedure have likely been used in various studies over the years. ABSOLUTE also used very similar principles to estimate per mutation CCFs. Those caveats about novelty aside, I am not aware of any easily runnable tool that performs the task of copy number quality control proposed in this work. As such, I see it as a useful contribution.

We thank the reviewer for making us aware that ABSOLUTE can optionally use a similar principle to QC its calls (we comment on it below), and we do agree that no tool exists that can easily  perform the task of copy number QC as proposed in this work.

The tool has been reasonably tested using a combination of synthetic and real single cell data. Application to real world datasets was extensive, with some compelling examples shown. I do have some concerns about the selection of ABSOLUTE as the best inference procedure in a few examples which I will discuss below. Overall though, the testing was well done.

We reply to this below, in a point-by-point manner.

My primary concern with the manuscript in the current form is that the methods are difficult to follow. There are a few factors at play. First, notation is not established and consistently used. Second, several sections interleave interpretation of results into the methods making it difficult to follow the exact design choices. Third, there are several typos and overloaded notations which are challenging. I provide a few examples in my detailed notes below.

Following the suggestion of this reviewer, we have largely re-written the most important part of the key methods in the manuscript (especially the core of Section “Simple clonal CNAs”). We think that we now present a version that is more coherent in terms of intuition and mathematical terminology, and that is easier to grasp and clear to read through.

We thank the review for pointing to several portions of the text that were clearly confusing.

**Detailed notes:**

-There were no page numbers so pointing to specific sections is tricky.

 We added page numbers.

- I am somewhat concerned that there is circularity in selecting ABSOLUTE, as it also performs similar CCF and peak finding for SNVs. There have been several updates to the software since it was published and it is unclear whether SNVs contribute to the selected CNV solutions. It would be helpful for the authors to clarify this.

As the reviewer pointed out, the original formulation of ABSOLUTE allows for the optional inclusion of SNVs to support the inference, along with a pre-computed prior model of copy number substitutions. In our paper we used data released by the PCAWG consortiums where the parameters used to run ABSOLUTE are not reported (see for instance the Dentro et al. paper <https://www.sciencedirect.com/science/article/pii/S0092867421002944?via%3Dihub#app2>). We suspect indeed that the tool has been run with this modality, because the results are very good and different from other methods.

However, we would like to precise that, we were not able to access recent versions of the ABSOLUTE documentation at <https://www.genepattern.org/>, the new Broad Institute website, where the tools seem to have become unsupported since November 2019.

Despite this overlap with ABSOLUTE, which we comment in the Methods Section (TCGA WES data) and in the Main Text, we believe that the QC algorithms of CNAqc can be useful to carry out an orthogonal validation of ABSOLUTE (or other) inference algorithms, as the problem is generally challenging and prone to errors.

- I am unclear on the use case for computing CCFs with this tool. The authors suggest that it could be helpful to QC SNVs which are poor fits. But these could just be from sampling noise, which is already modelled by tools such as PyClone-VI.

We agree with the reviewer that deconvolution-based tools compute better CCFs as they also remove observational noise. In fact, we often use PyClone as well to determine “per-clone CCFs“. However, some tools (e.g., CCube), compute “per-mutation CCFs” which retain the Binomial observational noise (which originates from the VAF one). We also observed that large consortia like PCAWG released mutation data with per-mutation CCFs accompanied.

When analysing these data we observed a number of scenarios where there could be errors or misunderstanding in the computation of these metrics. We already report our considerations in Section “Comparison to deconvolution methods” (“Simulations, validation and comparison to deconvolution tools”) and in Supplementary Figure S16. There, we make explicit reference to a scenario in which Ccube mistakes per-mutation CCFs, and invariably fails downstream subclonal deconvolution.

For this reason, since we wanted to develop a tool that was general  enough to QC data commonly available in the field, we thought of adapting equation (1) to compute CCFs. Our procedure can be used to inspect per-mutation CCF estimates computed by some tools that we think have systematic biases. We do feel that this is a desirable feature for a QC tool.

We now made this more explicit on the opening of the section “CCF estimation”.

- Taxonomy section. Are sex chromosomes considered? Presumably the definition of simple and complex will differ for males.

We thank the reviewer for asking for a clarification regarding how sex chromosomes are treated in CNAqc. In our model the definition of simple and complex CNA for sex chromosomes is the same as for the other ones.

The unique difference is that, in absence of aneuploidy, chromosome X is expected to be called 2:0 for females and 1:1 for males. This difference does not affect the inference because these states are supported by the tool, and therefore can be processed by the standard CNAqc algorithms (the same applied to other chromosomes).

We now report this in Section “CNAqc” of the Supplementary Material.

- Simple CNA section. It is suggested whole genome duplications can be detected with SNVs. I see that this would induce extra peaks, but it might be worth providing an example for the reader.

In Section “Simple clonal CNAs” we have added an example explaining the advantage of VAF analysis with respect to BAF in detecting whole genome duplication events, both above and below formula (1).

- Simple CNA section. The equation 1 <= m <= n_b seems superfluous given the constraint 1 <= m <= n_a and by definition n_a >= n_b.

We agree and opted to replace the two inequalities 1<= m <= n_a and 1<= m <= n_b with a single inequality 1<= m <= n_a <= n_b.

- Simple CNA section. $v_2(2)=0.5$ does not make sense as it implies a CCF of 2.

We thank the reviewer for noticing the mistake in the formula. We fixed the typo and wrote v_2(1) = 0.5, which means that peak of clonal mutations with double multiplicity (m = 2) is expected to be at VAF=0.5 in the considered case of whole-genome duplication segments for pure samples ( = 1).

- Simple CNA section. Indels are mentioned as being compatible with the method. However, the VAF of indels is usually noisier due to alignment bias. Can the authors comment?

Indels are compatible with the method and they can be used together with the SNVs for peak calling as they follow the same theoretical distribution. Nevertheless, we agree that indels are susceptible to alignment problems, and as such their VAFs can be more noisy. In fact, in our best practices in the lab, we often filter them out and in general compare CNAqc runs with SNVs, to runs with SNVs and indels.

We have added a comment on this in the section “Simple Clonal CNAs”, warning the user of possible complications with indels.

- Conversion among VAF … section. The notation is heavily overloaded here. For example $v_m(\pi)$ suggests purity is being used where previously CCF was the argument. There are also several epsilons, perhaps different well defined symbols would be clearer.

We thank the reviewer for noticing an important detail in the notation to be more clearly explained. We added the comment “Here, c  is the cancer cell fraction (CCF). To simplify notation, we will use vm() to represent VAF peaks of clonal mutations”, right after Equation (1).

To avoid confusion regarding the various error terms, we named , v, the expected error on purity, corresponding propagated error in VAF space, and VAF error tolerance. We instead denoted with the imputed error on purity in tests with simulated data.

- Sample level score section. Why are the sample sample metrics allowed to be positive or negative. Is there not a risk of different values cancelling, leading to erroneously low values. I would have thought an absolute value in equation 1.5 would make more sense.

The score is a summation per copy state and, for this reason, it can be distributed around 0 with both positive and negative values. One immediate advantage of this is that the score sign can inform on whether the input purity is lower (negative score), or higher (positive score) than 0. In this sense, a gradient on purity space can be determined by looking at the sign, which would be otherwise undetectable if we used an absolute value. This motivation was strongly driving our choice, as it allowed us to implement a purity-adjustment automatic pipeline based on the sign of the score (<https://caravagnalab.github.io/CNAqc/reference/Sequenza_CNAqc.html> and described in Section “QC-powered automatic copy number calling pipeline”).

As the reviewer points, however, the score is reporting the contribution to the errors of each used copy state, which can be a limitation in some scenarios. If for example a certain configuration fits well to data, and another does not, the summation might yield 0, and confound the result. This is one of the reasons we have implemented the following features in CNAqc:

- We report the score per copy state, which is the single contribution to the score, as shown here (<https://caravagnalab.github.io/CNAqc/reference/analyze_peaks.html>) and reported in this screenshot below. This helps the user determine if any copy state contributes to the result in a decisive way.

- We provide a function to split a whole genome into its chromosomes, which makes it easier to check if particular errors are due to small chunks of the segmented genome (<https://caravagnalab.github.io/CNAqc/reference/split_by_chromosome.html>).

The score proposed by the reviewer would have issues to be considered as well. For instance, we would have to consider that for every copy state – even under perfect data – we would have some variability in the scores and we would never hit a perfect 0. In this case, while for positive/negative scores little fluctuations average out, the absolute sum of many small terms might confound, as the summation of many small variances might yield the illusion of imprecise fits, a situation opposite (yet symmetrical) to the one predicted by the reviewer.

Overall we preferred to include a positive/negative score, coupled with two features that allow the user to avoid decisive confusion regarding the interpretation of our QC score.

*Output of CNAqc, in the red circle we highlight the scores reported per copy state, summed up together to yield the final CNAqc score. The score of each copy state can be negative or positive.*

- Same section. The subscript on the sum of $\lambda_k^FAIL$ is $P_k$ and should be $F_k$ I believe. This affects equation 1.6.

We have corrected both equations.

- I had some confusion when understanding CCF of clones. In some cases it refers to the SNVs and in others the CNV segments. A clear notation to distinguish the two and clear delineation in the text would help.

We fixed the ambiguity in terminology and notation that we found in section Genomics England WGS data in supplementary text. In particular, we used CCF to indicate the fraction of a mutation in a sample and subclonal proportion to indicate the fraction of a copy number subclone in the tumour. The latter concept is explained in the section dedicated to Subclonal CNAs in supplementary text.

- I have some concerns about peak detection. In cases of closely spaced peaks, stochastic sampling could provide the elusion of a single peak, albeit with a longer tail. This is a common feature in mixture models. Is the method robust to this or will it miss these peaks?

We provide a clarification regarding the robustness of the algorithm.

If the purity and coverage are reasonably high, peaks in VAF space are well separated (as of equation 1) and errors in peak detection should not occur. However, in case of low purity and/or low coverage samples we agree with the reviewer that peak detection is more challenging because the space among peaks is more narrow, and detecting peaks that are close is challenging. We reassure the reviewer that to assess the robustness of peak detection we performed tests on simulated datasets for different values of purity and coverage, including scenarios that are challenging (low coverage/low purity). Clearly, errors on peak detection can occur more frequently in low-quality samples, and the performance measures we report in the manuscript (page 35 in Section *Peak detection*) reflect these limitations, even if we observe a good performance of the tool in many reasonable working scenarios.

For the sake of completeness we report below an example of peak detection for challenging scenarios where, in one case, the algorithm works, and in another it fails.

*(top) Simulated tumour with purity 0.4 and coverage 30x. We adopted a tolerance of 3% on purity and error of 3% on the ground truth value. In this example CNAqc correctly passed the sample and was able to recognize the peaks also for karyotypes 2:1 and 2:2 , where the algorithm had to match two close peaks in VAF space. (bottom) Smulated tumour with purity 0.4 and coverage 30x. We adopted a tolerance of 3% on purity and error of 3% on the ground truth value (same parameters of panel a). In this example CNAqc did not identify the correct peaks for karyotypes 2:0 and 2:1 and failed to pass the sample.*

- Figure 5. A capital D is used in the caption which does not match the rest of the panel labels.

We have corrected the typo.

- Supp Figure 23. Panel e is not labelled or mentioned in the caption.

Done.

**Second round of review**

**Reviewer 1**

The authors' response and revisions have satisfactorily addressed my comments.

**Reviewer 2**

The authors have addressed my main concerns and greatly improved the clarity of the manuscript. The only minor point of confusion left for me is the new sentence regarding sex chromosomes.

"Note that for sex chromosomes we expect, in absence of aneuploidy, chromosome X to be 2:0 for females and 1:1 for males."

I would have thought females where heterozygous diploid (AB) i.e. 1:1 and males where just A i.e. 1:0.

Other than that minor comment everything else looks to be addressed.
